# LEMming: A Linear Error Model to Normalize Parallel Quantitative Real-Time PCR (qPCR) Data as an Alternative to Reference Gene Based Methods

**DOI:** 10.1371/journal.pone.0135852

**Published:** 2015-09-01

**Authors:** Ronny Feuer, Sebastian Vlaic, Janine Arlt, Oliver Sawodny, Uta Dahmen, Ulrich M. Zanger, Maria Thomas

**Affiliations:** 1 Institute for System Dynamics, University of Stuttgart, Stuttgart, Germany; 2 Department of General, Visceral and Vascular Surgery, Experimental Transplantation Surgery, Jena University Hospital, Jena, Germany; 3 Leibniz Institute for Natural Product Research and Infection Biology, Hans Knoell Institute, Jena, Germany; 4 Dr. Margarete Fischer-Bosch Institute of Clinical Pharmacology, Stuttgart, and University of Tuebingen, Tuebingen, Germany; Technische Universität Dresden, Medical Faculty, GERMANY

## Abstract

**Background:**

Gene expression analysis is an essential part of biological and medical investigations. Quantitative real-time PCR (qPCR) is characterized with excellent sensitivity, dynamic range, reproducibility and is still regarded to be the *gold standard* for quantifying transcripts abundance. Parallelization of qPCR such as by microfluidic Taqman Fluidigm Biomark Platform enables evaluation of multiple transcripts in samples treated under various conditions. Despite advanced technologies, correct evaluation of the measurements remains challenging. Most widely used methods for evaluating or calculating gene expression data include *geNorm* and ΔΔ*C*_*t*_, respectively. They rely on one or several stable reference genes (RGs) for normalization, thus potentially causing biased results. We therefore applied multivariable regression with a tailored error model to overcome the necessity of stable RGs.

**Results:**

We developed a RG independent data normalization approach based on a tailored linear error model for parallel qPCR data, called LEMming. It uses the assumption that the mean *C*_*t*_ values within samples of similarly treated groups are equal. Performance of LEMming was evaluated in three data sets with different stability patterns of RGs and compared to the results of *geNorm* normalization. Data set 1 showed that both methods gave similar results if stable RGs are available. Data set 2 included RGs which are stable according to *geNorm* criteria, but became differentially expressed in normalized data evaluated by a t-test. *geNorm*-normalized data showed an effect of a shifted mean per gene per condition whereas LEMming-normalized data did not. Comparing the decrease of standard deviation from raw data to *geNorm* and to LEMming, the latter was superior. In data set 3 according to *geNorm* calculated average expression stability and pairwise variation, stable RGs were available, but t-tests of raw data contradicted this. Normalization with RGs resulted in distorted data contradicting literature, while LEMming normalized data did not.

**Conclusions:**

If RGs are coexpressed but are not independent of the experimental conditions the stability criteria based on inter- and intragroup variation fail. The linear error model developed, LEMming, overcomes the dependency of using RGs for parallel qPCR measurements, besides resolving biases of both technical and biological nature in qPCR. However, to distinguish systematic errors per treated group from a global treatment effect an additional measurement is needed. Quantification of total cDNA content per sample helps to identify systematic errors.

## Introduction

Fluorescence-based quantitative real-time PCR (qPCR) is the commonly accepted gold standard to quantitate the amount of mRNA transcripts in biological samples. Benefits of this procedure over conventional methods for measuring RNA include its sensitivity, large dynamic range and its potential for high throughput as well as accurate quantification [1]. To achieve final correctly evaluated results, however, appropriate normalization strategies are required to control the experimental errors introduced during the multistage process from RNA extraction, processing and to final evaluation.

Depending on the number of measured gene transcripts and samples the available normalization strategies can be roughly divided into three groups, (i) knowledge-driven approaches, (ii) data-driven approaches and (iii) modeling approaches. Knowledge-driven approaches are usually applied to small data sets that measure only few gene transcripts in a limited number of samples. In such cases, preselection of internal standards is used for data normalization. Usually, such an internal standard is represented by a number of reference genes (RGs) known to be stably expressed under the different experimental conditions. While a single RG is sufficient for data normalization under ideal assumptions, according to MIQE guidlines [2] it is best practice to rely on the information of multiple RGs for calculation of a sample specific normalization factor. To this end, a number of methods have been proposed for the identification of stable reference genes. Three of the most prominent ones are *geNorm* [3], *NormFinder* [4] and *BestKeeper* [5]. While these methods differ in detail, the basic principle remains the observation and evaluation of inner- and inter-sample/group variation among the preselected RGs. Thus, it is presumed that the selected RGs are not regulated under the observed experimental conditions and most of all, that they are independent from each other.

The second group of approaches encloses the data-driven normalization methods such as quantile normalization and rank-invariant set normalization. These methods where initially developed for the normalization of high-throughput gene expression data such as microarrays. In their study, Mar et al. [6] proposed that these data-driven normalization methods are applied to data sets with a minimum of 50 primer pairs per sample. Similar to the knowledge-driven approaches, there is a number of presumptions that are made when using data-driven normalization methods. Quantile normalization for example assumes that on average the distribution of the expression values within the cell remains about constant across the different samples. Given a small number of genes, estimation of the quantile distribution from different samples becomes prone to outliers.

The third group of methods is formed by modeling approaches, i.e., approaches that model the gene expression values as a composition of the true gene expression and various effects of technical and biological nature. For example Steibel et al. [7] use linear mixed models to estimate different effects on gene expression. Matz et al. [8] use generalized linear models. They model the initial transcript copy number and use a Poisson-lognormal distribution of errors in order to estimate different effects on gene expression.

The use of RGs in a knowledge-driven approach is widely spread for normalization and is implemented in many software tools for qPCR data analysis. Software tools are reviewed by Pabinger et al [9]. A basic technique for normalization is the ΔΔ*C*_*t*_ method described by Livak and Schmittgen [10] and Pfaffl [11]. The ΔΔ*C*_*t*_ method relies on a single RG. Current tools like *qBase^+^* [12], *DAG Expression* [13] and *SASqPCR* [14] use multiple RGs for normalization. They implement a normalization strategy that originates from Vandesompele et al [3], who suggested the use of multiple RGs. Their tool *geNorm* allows the most appropriate RGs to be chosen. *geNorm* calculates gene expression stability values (M) values for each gene, being values below 1.5 the most stable RGs. Additionally, it estimates the normalization factor (*NF*_*n*_) using the geometric mean of expression levels of *n* best RGs, given by the pairwise variation (V) analysis, by selecting a cut-off (0.15) below which *n* RGs should be used for normalizing.

Bas et al [15] and Tricarico et al [16] demonstrate that experimental results depend on the choice of RGs. The reason for that is responsiveness of RGs towards experimental conditions, which has often not been tested previously.

Numerous studies [17–19] have shown that common RGs, like genes for *albumin, actins, Gapdh, tubulins, cyclophilin* as well as *18S* or *28S* rRNA, may vary under experimental conditions. Furthermore, the difficulty of finding reliable RGs increases with the number of experimental conditions used in one experimental setup. The usage of multiple RGs can reduce the impact of single outliers in a RG measurement on the whole sample. However, in case of responsiveness of RGs towards experimental conditions, the number and the choice of RGs will alter the results. A distortion of results can be identified if the mean of raw values compared to processed values is shifted under an experimental condition.

To solve these problems we introduce a new modeling approach based on multivariable regression, which is specialized for the normalization of parallel qPCR data. Spurgeon et al. [20] introduced a high throughput approach for qPCR with microfluidic dynamic arrays. It enables to assess 48 or 96 transcripts in 48 or 96 samples on one array in parallel. The design of the microfluidic arrays for parallel qPCR measurements allows application of a tailored linear error model which circumvents the dependence on RGs. This approach is called LEMming (linear error model) and uses linear mixed models. LEMming allows the exclusion of technical errors, the retention of the biological variation and the exclusion of systematic errors, if they are identified by external measurements (e.g. cDNA quantification). LEMming reduces the variance in measurements while exerting no influence on the mean value of gene measurements under an experimental condition. Here we use LEMming to analyze three data sets with different stability patterns of RGs. Furthermore, we use LEMming to assess the contribution of several sources of variation in qPCR data. LEMming is implemented in the freely available language R and can be applied by customization of an R-script to a data set.

## Materials and Methods

We selected three data sets with different stability patterns of RGs reflecting different experimental situations:

**Data set 1 (DS1):** The first experiment was designed for precise quantification of various technical errors, as well as for the evaluation of reverse transcription (RT) reaction variability. In detail, HepG2 cells were treated with peroxisome proliferator activating receptor alpha (PPARa) agonist (N = 4). HepG2 cells, passage 11, were plated out in 12-well plates at 5 × 10^5^ cells/well density in high-glucose DMEM medium (11965-092, Life Technologies), containing Glutamine and 10% FCS. 24h later, 100 μM WY14,643—4-Chloro-6-(2,3-xylidino)-2-pyrimidinylthioacetic acid (C7081, Sigma) or solvent control, DMSO—Dimethyl sulfoxide (D9170, Sigma) were added to 4 wells each (DMSO for the samples A, B, C, and D; WY14,643 for the samples E, F, G, and H). After 48h of treatment, all wells were lysed using 300 μL of RLT buffer from RNeasy kit (74104, Qiagen) and RNA was immediately isolated following manufacture’s instructions. Next, three independent RT reactions have been performed from each sample, resulting in 3 cDNAs for each sample (i.e. A1, A2, A3, etc). Finally, 24 generated cDNAs were run in technical duplicates on a 48×48 microfluidic dynamic array. To sum up, DS1 has four biological replicates, three technical replicates for each cDNA conversion step and two replicates for the qPCR step per condition. DS1 is available in S1 Table.

**Data set 2 (DS2):** DS2 was selected because of its technical replicates and its seemingly stable RGs in raw data. Male mice (C56Bl6N) (20–25g, Charles River, Sandhofer Weg 7, Sulzfeld, Germany) were employed in this study. The animal protocol was reviewed and approved by the “Thüringer Landesamt für Lebensmittelsicherheit und Verbraucherschutz—Dez.22/Fachgebiet Tierschutz/Tierarzneimittel” Germany (§15 Tierschutzgesetz, Reg.-Nr. 02-009/14). Liver tissue samples, generated in a vivo experiment in mice, were investigated under the influence of starvation on the kinetic expression level with 7 housekeeping genes (*Actb*, *Itih4*, *Ywhaz*, *Rps13*, *Ppia*, *Hprt1*, *Eef1a1*). The mice were denied access to food for a period of 0, 24, 48 or 72 hours (*n* = 6 each group). The mice were housed under standard animal care conditions and access to water over the whole time. After observation times liver tissue samples were taken. The mRNA was extracted from the frozen tissues using the Qiagen RNeasy Mini Kit (Valencia, CA). RNA quantity was measured using Nanodrop (Thermo Scientific, Waltham, MA). RNA integrity number (RIN) was checked by Agilent 2100 Bioanalyzer (Agilent Technologies, Santa Clara, CA) and was above 8.5 for all samples. cDNA synthesis was performed with 2 μL of 50 ng/μL total RNA, 1 μL of 10 × TaqMan RT Buffer, 2.2 μL 25mM MgCl_2_, 2 μL of 2.5 nM dNTP-Mix, 0.5 μL of 50 μM random hexamers, 0.2 μL of RNase Inhibitor, 0.25 μL of 50 U/μL Multiscribe reverse transcriptase and 1.85 μL RNase-free water. All reagents were purchased from Applied Biosystems (TaqMan Reverse Transcription Reagents: N808-0234). The reaction mixtures were mixed with RNA and incubated by 25°C for 10 min, 48°C for 30 min and then 95°C for 5 min. All RNA samples were transcribed twice to detect systematic errors during the cDNA synthesis. In the end, 48 generated cDNAs were put on in technical duplicates on a 96×96 microfluidic dynamic array. Likewise, all 46 primers were put in twice on the array as technical replicates. The DS2 is available in S2 Table.

**Data set 3 (DS3):** DS3 was selected as a complex *in vivo* experiment where no stable RGs were identified in raw data. Male inbred Lewis rats (Lewis/HanTMHsd) (250–350g, Central Animal Laboratory, University Hospital Essen, Germany) were employed in this study. The animal protocol was reviewed and approved by the “Thüringer Landesamt für Lebensmittelsicherheit und Verbraucherschutz—Dez.22/Fachgebiet Tierschutz/Tierarzneimittel” Germany (§15 Tierschutzgesetz, Reg-Nr. 02-042/10).

Liver tissue samples, generated in a complex *in vivo* experiment in rats, were used to investigate the influence of different experimental conditions (surgery, drug treatment, vascular congestion) on the kinetic expression level of 9 commonly used RGs (*Ywhaz*, *Tfrc*, *Rpsl13*, *Rpl32, Rpl27, Mrps18a, Hprt1, Gapdh* and *Actb*).

The experiment was originally dedicated to explore the effect of vaso-active drugs (L-NAME, Molsidomine, saline) on the recovery from focal outflow obstruction in rats with portal hypertension due to liver resection (named: ligation/PH). Focal outflow obstruction was induced by ligation of the right median hepatic vein. Animals subjected to ligation only (named: ligation) and untreated rats (named: untreated) were used as controls. Samples were obtained after observation times of either 0h, 24h, 48h and 7d from 3 different locations of the right median lobe: the obstructed zone, the border zone and the normal zone. Further details regarding the experimental design and procedures are published in Hai et al. [21].

The mRNAs were extracted from the frozen tissues based on manufacturer’s protocols from Qiagen RNeasy Mini Kit (Valencia, CA). RNA quantity was determined using Nanodrop (Thermo Scientific, Waltham, MA) and the quality was assessed by Agilent 2100 Bioanalyzer (Agilent Technologies, Santa Clara, CA). RNA integrity number (RIN) was above 8.5 for all samples. cDNA synthesis was performed as described in DS2 with reagents by Applied Biosystems (TaqMan Reverse Transcription Reagents: N808-0234).

### Taqman qPCR measurement using Fluidigm Biomark

Pre-designed validated Taqman Gene expression assays were purchased from Life Technologies (Darmstadt, Germany) for the detection of human (DS1), mouse (DS2) and rat (DS3) transcripts. Gene expression assays are listed in the according data set table (S1, S2 and S3 Tables). The company claims assessed PCR efficiency of the assays to be 100 ± 5%. We nevertheless analyzed the amplification efficiency of three exemplary assays on our own using a dilution series of a control cDNA sample and could reproduce the data provided by the manufacturer.

For the analysis of DS1, following genes were used as potential reference genes: *ACTB*—Actin beta (Hs01060665_g), *TBP*—TATA box binding protein (Hs00427620_m1), *POLR2A*—polymerase (RNA) II (DNA directed) polypeptide A (Hs00172187_m1), *GAPDH*—Glyceraldehyde-3-phosphate dehydrogenase (Hs02758991_g1), *HMBS*—hydroxymethylbilane synthase (Hs00609296_g1) and *RPLP0*—ribosomal protein, large, P0 (Hs02992885_s1).

The assays of 9 reference genes used for DS2 and DS3 analysis are: *Actb*—Actin beta (Rn00667869 m1), *Gapdh*—Glyceraldehyde-3-phosphate dehydrogenase (Rn01775763 g1), *Hprt1*—Hypoxanthine phosphoribosyltransferase 1 (Rn01527840 m1), *Mrps18a*—Mitochondrial ribosomal protein S18A (Rn01511938 m1), *Rpl27*—Ribosomal protein L27 (Rn00821099 g1), *Rpl32*—Ribosomal protein L32 (Rn00820748 g1), *Rps13*—Ribosomal protein S13 (Rn02606812 g1), *Tfrc*—Transferrin receptor (Rn01474701 m1) and *Ywhaz*—Tyrosine 3-monooxygenase/tryptophan 5-monooxygenase activation protein, zeta (Rn00755072 m1).

qPCR was carried out using the 48×48 (for the DS1) and 96×96 (for the DS2 and DS3) Dynamic Array (Fluidigm Corporation, CA, USA) with minor modifications to the manufacturer’s protocol found in Spurgeon et al. [20]. Briefly, 2.7 μL sample mixture, 0.3 μL 20X GE Sample Loading Reagent (Fluidigm PN 7385000746) and TaqMan Universal PCR Master Mix (2X) (Applied Biosystems, PN 744304437) was used. After, 3.3 μL of Assay mix was prepared with 3.3 μL 2× Assay Loading Reagent (Fluidigm PN 85000736). After loading, the chip was placed in the BioMark Instrument 76 (GE96X96 Standard v1.pcl—protocol file) for PCR and run was set for an initial cycling at 50°C for 2 min, 70°C for 30 min and 95°C for 10 min, followed by 40 cycles at 95°C for 15 sec and 60°C for 60 sec. Raw data was analyzed with Real-Time PCR Analysis Software in the BIOMARK instrument (Fluidigm Corporation, CA, USA).

### Basics of parallel RT-qPCR

A dynamic array for Fluidigm Biomark measurements has 48 or 96 probe slots and 48 or 96 sample slots. The center of the chip is an integrated fluidic circuit (IFC), which is a network of fluid lines, NanoFlex valves and reaction chambers [20]. The fluid lines with the associated valves on the IFC combine each probe with each sample in a total number of 2304 or 9216 individual reaction chambers (RC) each carrying out a qPCR reaction. An experiment can include numerous arrays, where various tissues and treatments are measured.

A protocol for quantification of mRNA by real-time RT-qPCR (reverse transcription (RT) followed by polymerase chain reaction (PCR)) is given by Nolan et al. [22]. The target mRNA must be extracted and converted to cDNA in a reverse transcription process. Starting with the cDNA concentration *m*_0_, the cDNA is roughly doubled (PCR efficiency *E* is around 2) in every PCR cycle. The cDNA generates a fluorescence signal. When the fluorescence signal exceeds a certain threshold *T* the number of cycles *C*_*t*_ are registered.

$$T=m_{0}E^{C_{t}}$$ (1)

A positive shift in the *C*_*t*_ value indicates a lower mRNA starting concentration *m*_0_.

#### Sources of variation

RT-qPCR measurements have technical and biological sources of variation. Sources of technical variation are pipetting errors of probes and samples, as well as errors within the steps of RNA extraction, reverse transcription (RT) and qPCR. The most critical step is the RT, which contributes most to the variation of mRNA measurements [23]. The errors due to RT and qPCR steps may vary from gene to gene. Biological variation of gene expression may vary between treatment conditions and is especially increased after stimulus experiments. As we will show, the distinction between technical errors and biological variation improves the reliability of measured differential expression especially in cases of small effects.

### ΔΔ*C*_*t*_ method and *geNorm*

**The ΔΔ*C*_*t*_ method** is described by Livak and Schmittgen [10] and Pfaffl [11]. It determines the relative quantification of a target gene (*TG*) in comparison to a reference gene (RG). The relative expression ratio of a *TG* is calculated based on PCR efficiency *E* and *C*_*t*_ deviation of a sample (*s*) versus a control (*c*) [11]:

$$ratio=\frac{{\left(E_{TG}\right)}^{\left(C_{t}^{TG,c}-C_{t}^{TG,s}\right)}}{{\left(E_{RG}\right)}^{\left(C_{t}^{RG,c}-C_{t}^{RG,s}\right)}}$$ (2)

***geNorm*** is a method for the identification of the most stable RGs given a list of potential candidate RGs [3]. The method relies on the principle that the expression ratio between two ideal RGs remains constant over all samples. Variation in this ratio however corresponds to a decreased expression stability. This provides a ranking of the candidate RGs for their use as part of the normalization factor (*NF*). For each sample, *NF* itself is defined as the geometric mean of their expression values. The computation of expression stability and normalization factor as well as the calculation of *geNorm* normalized values that are comparable with raw and LEMming processed data is described in detail in S2 File section 2. Application of the ΔΔ*C*_*t*_ method for normalization as well as identification of stable RGs using *geNorm* require complete data sets without missing values. Therefore, imputing of missing values was performed using K-nearest neighbor imputing [24] with default values (K-value of 10) as provided by the R-package impute.

### Linear error model (LEM)

A framework for data analysis of relative qPCR using linear mixed models has been introduced by Steibel et al [7]. This framework is used to test for inferences in complex experimental layouts but is still using RGs. We also use a linear error model, but focus on the generation of a correct tool independently of RGs. The matching of probes and samples in the reaction chambers of microfluidic arrays imply an experimental design that allows to determine probe and samples errors in a tailored error model. Here, the sequence of estimated effects plays an important role.

We distinguish two types of technical errors: The probe error per array (*ϵ*_*P*:*A*_) is the effect or mean of all 48/96 sample measurements of a gene on an array. Pipetting of the probe and the channel for probe transportation on the array influence *ϵ*_*P*:*A*_. The sample error (*ϵ*_*S*_) is the effect or mean of all 48/96 gene measurements of a sample. Sample preparation steps which are exclusive to a sample like pipetting, the channel for sample transportation and the RNA-extraction influence *ϵ*_*S*_. Both technical errors are systematic influences which are present in 48/96 measurements at a time. They can be excluded in order to reduce variance of all measurements.

Furthermore, systematic errors or batch effects $\tilde{\epsilon }$ can cause offsets. Systematic sample errors can be identified by single-stranded DNA quantification and considered in the error model (see S1 and S4 Files).

Other systematic effects like influence of treatment and biological variance are part of the measurement and should be retained for visualization. The treatment effect splits up into two parts, the global effect (Δ_*T*_) and the treatment effect per gene (Δ_*T*:*G*_). Biological variance and non-systematic technical errors are described by the variable *ϵ*.

To sum up, a measurement *Y* is composed of

$$Y=\underset{\tilde{Y}}{\underset{︸}{\epsilon_{PA}+\epsilon_{S}+\tilde{\epsilon }}}+\underset{\hat{Y}}{\underset{︸}{\Delta_{T}+\Delta_{T:G}+\epsilon }}$$ (3)

The different effects are estimated by linear model fitting. The variable $\hat{Y}$ collects influences which are retained. $\tilde{Y}$ describes the estimated technical errors which can be subtracted. Since parallel qPCR evaluates only a selection of genes, the average of *C*_*t*_ measurements may change with the treatment. This effect is the global effect Δ_*T*_ which need to be retained for $\hat{Y}$.

The different effects are estimated in the following order:

1. Estimate the probe error per array (*ϵ*_*P*:*A*_).
2. Estimate systematic errors/batch effects ($\tilde{\epsilon }$).
3. Estimate the treatment/tissue effect (Δ_*T*_).
4. Estimate the sample error (*ϵ*_*S*_).
5. Estimate the treatment effect per gene (Δ_*T*:*G*_).

It is important to stick to this order. If the sample error would be estimated first of all, systematic errors and treatment effects would be included in this sample error. Sticking to the estimation order above guarantees that Δ_*T*_ contains no systematic errors and is not removed by the sample error *ϵ*_*S*_. The variables $\hat{Y}$ are used to compute the −Δ*C*_*t*_ values of a gene under a treatment compared to the untreated group

$$-\Delta C_{t}=-\left(\hat{Y}-Mean\left[{\hat{Y}}_{untreated}\right]\right)$$ (4)

The fold change values are 2^−Δ*C*_*t*_^. They are visualized per gene and treatment group in boxplots with the fold change on a base-2 log scale. The displayed boxplots have a centered thick line which marks the median. Lower and upper bounds of the box are the quantiles *q*_25%_ resp. *q*_75%_. Red points are classified outliers (1.5 times outside of interquartile range below/above of *q*_25%_/*q*_75%_).

S3 File is an example R-script for using LEMming with data set 2. To assess the statistical significances of differences we used the function *ebayes* with option robust from the *limma* package [25].

#### Recommendations for using LEMming

We recommend to use a proper experimental design (e.g. latin square). Based on that systematic errors can be identified and excluded by using LEMming.

## Results

### Evaluation of data sets

#### DS1

**Fig 1:**
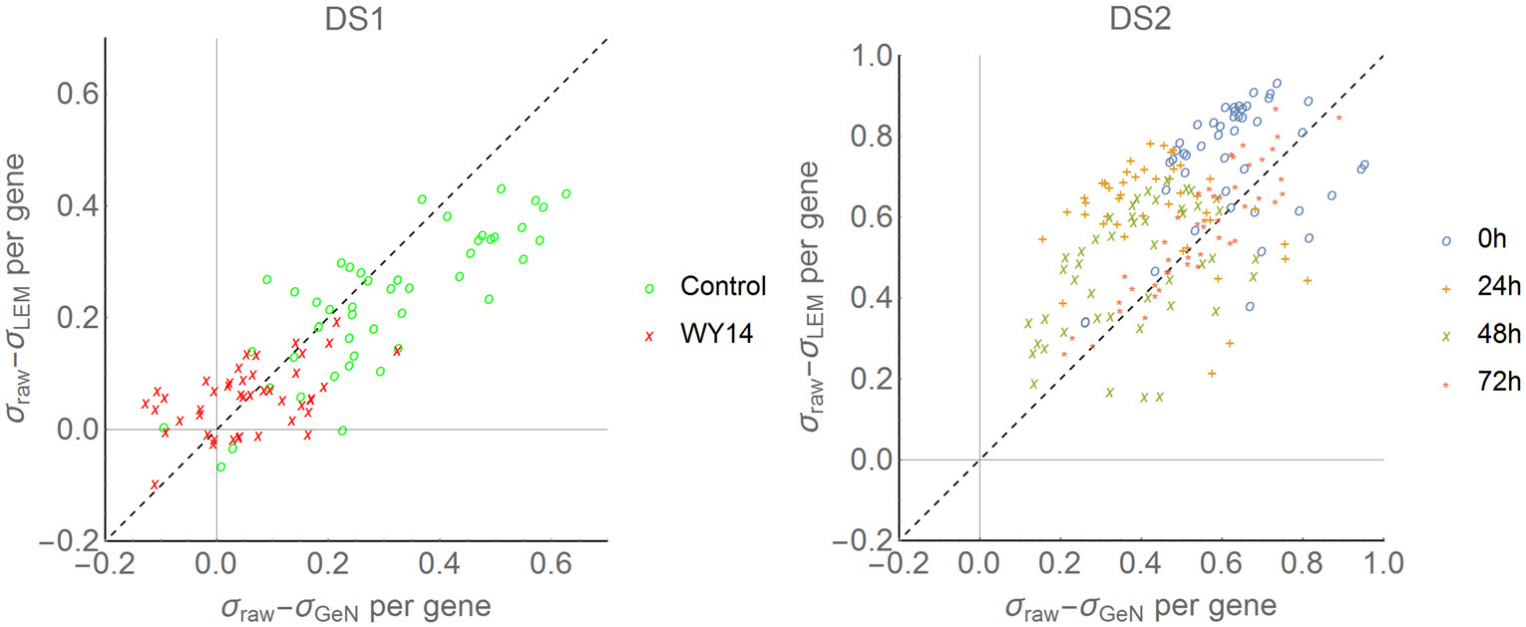
Comparison of standard deviations for *geNorm* and LEMming per gene in DS1 and DS2. The x-axis shows the difference between standard deviation (sd) of raw values and sd of *geNorm* processed data per gene. Accordingly the y-axis shows the difference of sd of raw values to LEMming processed data. If points are in the positive quadrant and above the dotted line, sd of LEMming processed data is smaller compared to sd *geNorm* and raw data.

We calculated the average expression stability and the pairwise variation of common RGs in DS1 according to *geNorm* (plots are shown in S1 File). *GAPDH* is the most stable RG which was used for normalization with the ΔΔ*C*_*t*_ method. The normalization factor (NF) calculated from the genes *GAPDH, HMBS, RPLP0* and *TBP* show a low pairwise variation compared with the NF that includes *POLR2A* (pairwise variation 0.15). Thus, there is no need to include *POLR2A* into the calculation of the *NF*. Consequently, we used the four most stable RGs for normalization with *geNorm*. Standard deviations (*σ*’s) per gene and condition were computed from raw data, from ΔΔ*C*_*t*_, *geNorm* and LEMming normalized data. The comparison of *σ*’s between raw data, ΔΔ*C*_*t*_ and LEMming normalized data is presented in S1 File. We tested the *H*_0_-hypothesis of equality of *σ*’s with the Wilcoxon signed-rank test. Std. devs of LEMming residuals per gene and condition are significantly smaller than std. devs of raw data (p ≤ 10^−11^) and std. devs of ΔΔ*C*_*t*_ normalized data (p ≤ 10^−6^). Additionally, we present a proof in S1 File, showing that the std. devs of ΔΔ*C*_*t*_ with a single RG is greater than of std. devs LEMming processed data. The boxplots per gene and condition comparing raw data, *geNorm* and LEMming processed data are presented in S1 File. The variance-mean plots in S1 File show a slight shift of the mean values for WY14,643 treated cells with *geNorm*, but not with LEMming. A comparison between std. devs of *geNorm* and LEMming processed data per gene is shown in Fig 1. The std. devs of control measurements are reduced on average by 26.8% ± 4.9 with *geNorm* and 23.7% ± 3.8 with LEMming compared to raw data. The std. devs of WY14,643 treated samples are reduced on average by 1.5% ± 9.7 with *geNorm* and 6.5% ± 2.8 with LEMming compared to raw data. Fig 1 shows few genes whose std. dev. is enhanced by usage of normalization techniques.

#### DS2

**Fig 2:**
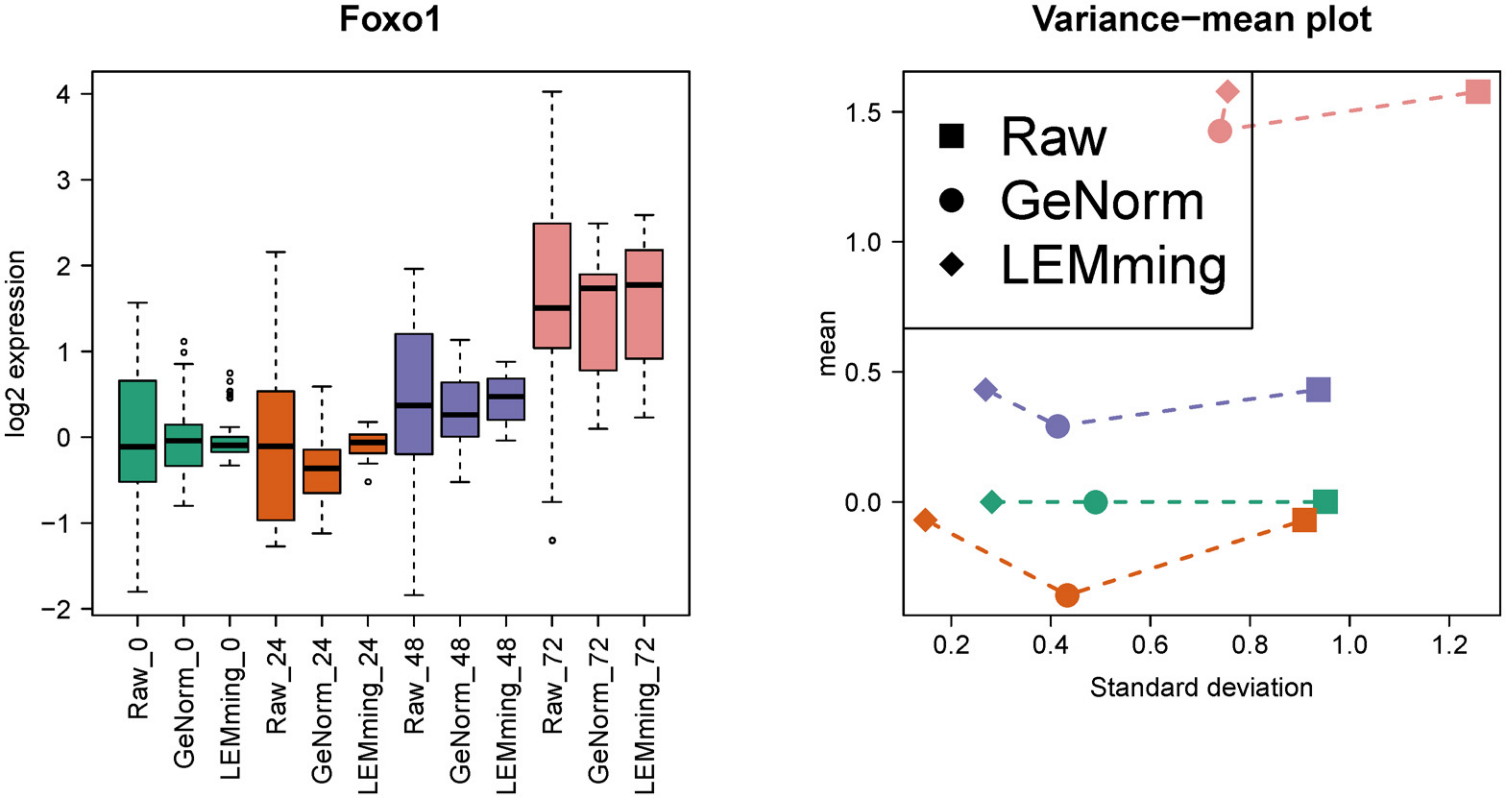
Example boxplot for *Foxo1* in DS2. Left: Log2-fold differential expression of the gene *Foxo1* in DS2. Conditions are 0h, 24h, 48h and 72 h. Right: Variance-mean plot showing the mean over the standard deviation per condition for *Foxo1*.

The average expression stability and the pairwise variation of common RGs in DS2 according to *geNorm* are presented in S2 File. The RGs *Eef1a1, Hprt* and *Ppia* were chosen for *geNorm* normalization (M-values below 0.36). We applied LEMming to DS2 (R-Script in S3 File) and found a systematic effect that relates to the variable cDNA in the data. This effect was validated by single stranded DNA quantification (details in S2 File) and was excluded with LEMming as a systematic batch effect ($\tilde{\epsilon }$). *geNorm* inherently considers this effect due to the normalization with RGs. An exemplary plot comparing the log2 fold differential expression for the gene *Foxo1* in DS2 for raw, *geNorm* and LEMming processed values is presented in Fig 2. The variance-mean plot in Fig 2 demonstrates that *geNorm* normalized data shows a displacement effect of the gene wise mean (Δ*C*_*t*_ of -0.28 at 24h, of -0.14 at 48h and -0.15 at 72h compared to 0h) which is present for all genes measured in DS2 (see S2 File).

T-tests on the three RGs in raw data have no significant results comparing the 24h, 48h and 72h condition to the 0h condition with a Bonferroni corrected *α*-level (smallest p-Value for *Eef1a1* for 0h vs 72h: *p* = 0.0502). In contrast, t-tests with *geNorm* normalized data have significant results for all three RGs (*Eef1a1*: 0h vs 48h *p* = 1.46 × 10^−9^, 0h vs 72h *p* = 1.51 × 10^−9^, other RGs have under all comparisons *p* < 10^−11^). LEMming normalized data shows also significant results for the three RGs (see S2 Table worksheet *LEM_diff*).

Std. devs of *geNorm* and LEMming processed data are compared in Fig 1. LEMming data displays significantly reduced std. devs when comparing to *geNorm* (sign test p ≤ 10^−11^). The mean reduction of std. dev. per gene and condition is 49.4% for *geNorm* and 50.5% for LEMming. LEMming excludes systematic effects that are responsible for 83.1% of variance of the median gene in this experiment.

#### DS3

The average expression stability and the pairwise variation of common RGs in DS3 according to *geNorm* are presented in S4 File. Raw, *geNorm* and LEMming processed data of RGs in DS3 is available as S3 Table. Data of other genes in DS3 will be published elsewhere under a biological issue. The plots of log2 fold differential expression comparing raw, *geNorm* and LEMming processed values and the according variance-mean plot is part of S4 File. Both *geNorm* and LEMming shift the mean values per gene and treatment, whereby the distance to the mean of raw data is smaller with LEMming. In LEMming this effect appears, because the data set is measured on three different arrays. The exclusion of batch effects with LEMming in DS3 is discussed in S4 File (subsection 1.1).

**Fig 3:**
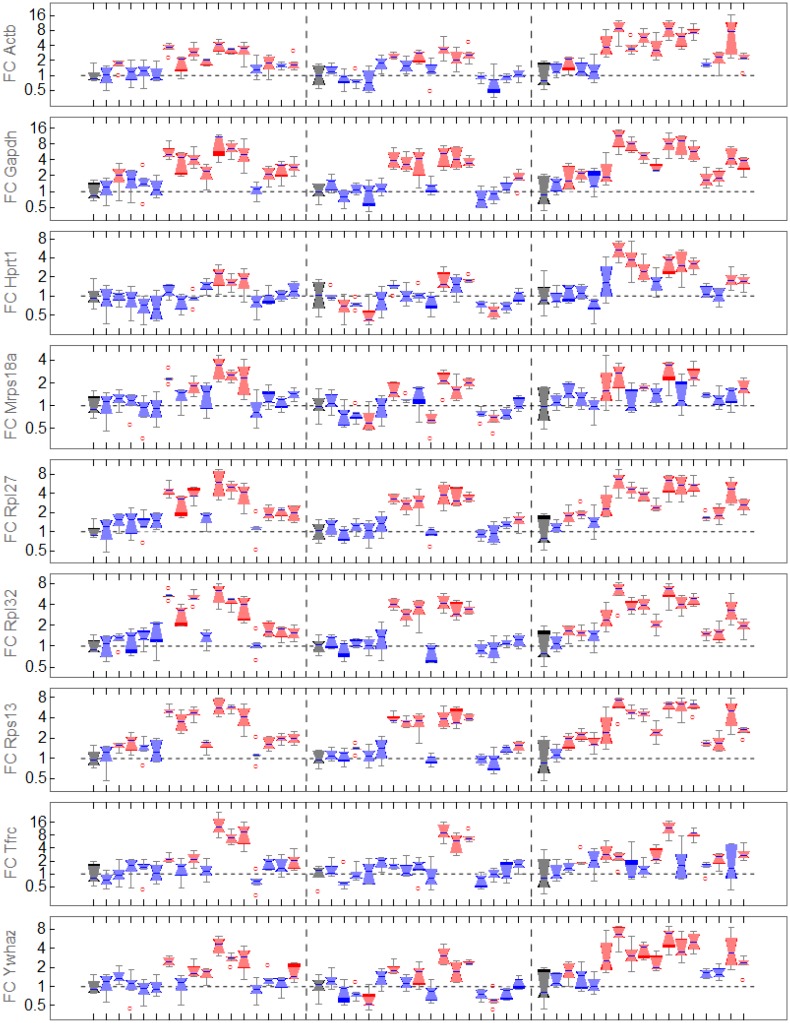
Raw data of common reference genes in data set 3. Boxplots of the untreated conditions are black, boxplots of treatment conditions (dedicated in S4 File) that are not significant differentially expressed compared to untreated are blue and boxplots of treatments with significant differentially expressed measurements are red. Significance was calculated by an unpaired t-test with unequal variances and Bonferroni corrected significance level *α* = 0.05/48. Outliers are marked by red circles.

The fold change values ($2^{-\Delta C_{t}}$) of RGs calculated from raw values are shown for various treatments in three tissue types in Fig 3. Statistical comparisons of raw values from untreated and treated conditions (unpaired t-test with unequal variances, Bonferroni corrected significance level $\alpha =\frac{0.05}{48}$) colorize the boxplots: conditions with significant differential expression are shown as red boxplots, other ones are shown as blue boxplots. The different experimental conditions in the plot are dedicated in S4 File. According to *geNorm* (and NormFinder) *Rpl27*, *Rps13* and *Rpl32* are the most stable RGs which were selected for calculation of the *NF* (details in S4 File). However, Fig 3 indicates that none of the RGs is stably expressed. We used two approaches to assess the stability of the proposed RGs (details in S4 File subsection 1.1). First, we used an external measurement (ssDNA quantification) to estimate the introduced error during cDNA conversion. Second, we made use of the experimental setup, estimated potential systematic errors and removed them. This provides a conservative estimate of the stability of the observed RGs. Both approaches failed in explaining the significant differences between experimental conditions for the RGs.

Using the three RGs in this case distorts the results as it is demonstrated by the variance-mean plot in S4 File. A particular example is the gene *Pcna* whose evaluation is disturbed by using the three RGs for normalization. *Pcna* is upregulated with raw data and LEMming processed data but is downregulated with *geNorm* normalized data under the condition ligation partial hepatectomy after 24h and 48h (see Figure J in S4 File). *Pcna* is used as marker for partial hepatectomy [26]. It is upregulated under these conditions [27], which was validated in microarray data (GEO GSE55434).

### Analysis of variance

**Fig 4:**
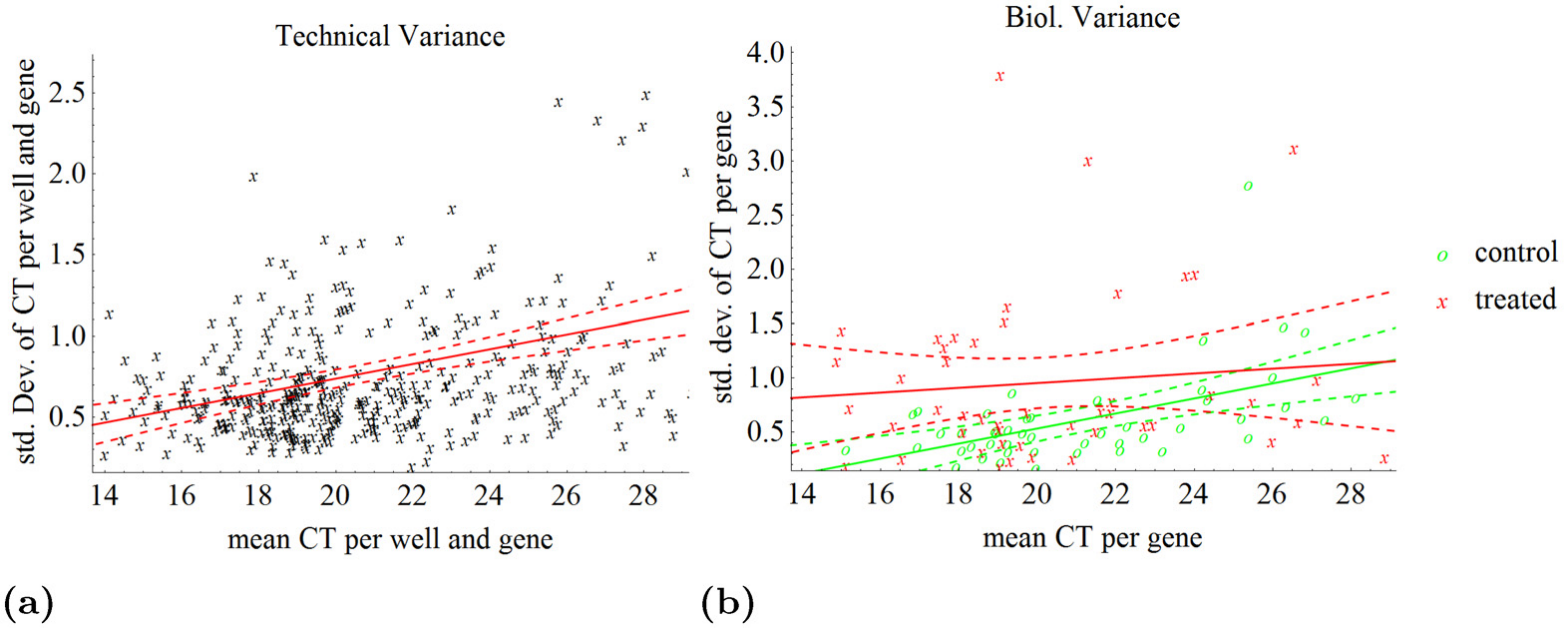
Technical and biological variances over mean value per gene. **(a)** Standard deviation of *C*_*t*_ value of each gene and well over the mean *C*_*t*_ value for data set 1 (DS1). Each gene is measured six times per biological replicate (3× cDNA and 2× PCR per cDNA). The regression line shows that the standard deviation increases with the *C*_*t*_ values (lower mRNA content). **(b)** Standard deviation of biological replicates over the mean *C*_*t*_ value for DS1. The mean of all six technical replicates is computed per biological replicate and gene. The standard deviation of these means is computed with 4 biological replicates for each gene. The biological variance is higher under treated conditions compared to the control conditions.

We analyzed how variance relates to the absolute *C*_*t*_-value with raw values of DS1 in Fig 4. With increasing *C*_*t*_-value, which means with lower mRNA content, the standard deviation of technical replicates increases (Fig 4(a)). The same phenomenon—called heteroscedasticity—was observed for variances of biological replicates (Fig 4(b)). Here the heteroscedasticity means that measurements with higher *C*_*t*_-values are less trustable than measurements with lower ones. The biological variance in the control group is significantly smaller compared to the treated WY14,643 group (ANOVA of standard deviation values per gene for the mean expression values of 4 biological replicates: p = 0.01).

**Fig 5:**
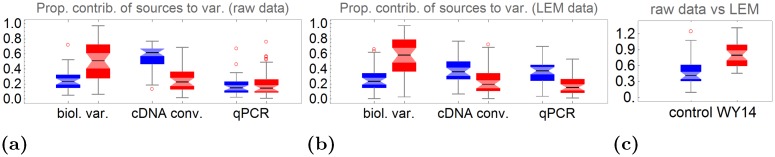
Contribution of biological variance, cDNA conversion error and qPCR error to the overall variance for data set 1. Proportions of variance contribution are estimated from raw data **(a)** and from residuals of LEMming **(b)**. Blue boxplots are measurements of the control group, red boxplots are measurements of WY14,643 treated cells.

DS1 and DS2 with its multiple technical replicates allow to further discriminate sources of variation in the data. Thus, the contribution of non-systematic technical errors (cDNA conversion error and qPCR error) to the sample error *ϵ*_*S*_ and the residuals of LEMming *ϵ* (Eq 3) can be resolved. We determined the contribution of biological variance, cDNA conversion error and qPCR error to the variance per gene in DS1 by an ANOVA. The analysis was performed directly with the raw data and with LEMming processed data. The proportions of variance contribution are shown in Fig 5.

The contribution of biological variance, cDNA conversion error and qPCR error to the overall variance varies from gene to gene. In raw data the cDNA conversion contributes most (median over all genes: 62%) to the overall variance, while biological variance only accounts for 23% and qPCR error for 15% of the overall variance (see Fig 5(a)). This is in accordance with published data [23]. The variance of LEMming residuals *ϵ* is explained by 24% (median) biological variance, 36% (median) cDNA conversion error and 38% (median) qPCR error in the control group (blue boxplots Fig 5(b)). The contribution of biological variance is significantly increased in the WY14,643 treated samples (red boxplots Fig 5) in both raw data and LEMming residuals.

**Fig 6:**
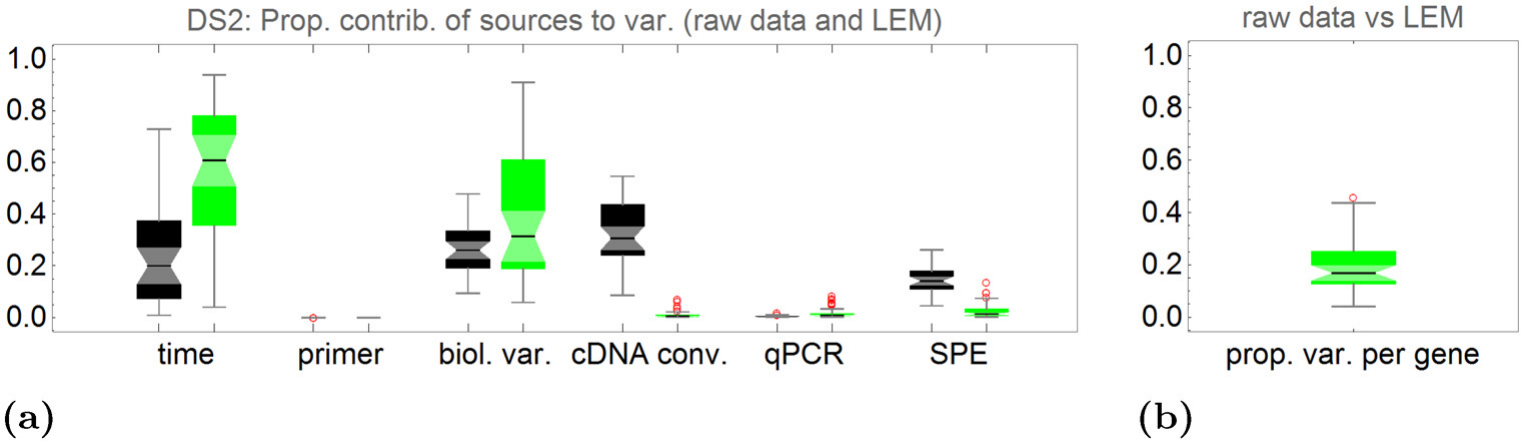
Proportional contribution of different effects to the variance of a gene in data set 2 (DS2). Black—raw data, Green—LEMming processed data. **(a)** Proportion of sum of squares associated to the effects time, primer pipetting, biological variance, cDNA conversion, qPCR error and sample pipetting error (SPE) resulting from a ANOVA for each gene. **(b)** Proportion of sum of squares of LEMming processed to raw data without the effect of time (treatment effect). The median is 16.9%, which means that LEMming excludes systematic effects that are responsible for 83.1% of variance of the median gene in this experiment.

The same analysis was performed with DS2 (see Fig 6). With DS2 it is possible to split up the sample error *ϵ*_*S*_ into a cDNA conversion error and a sample pipetting error. Here the cDNA conversion is the dominant variance source in the raw data. Due to the multiple technical replicates in the experiment, this effect is nearly completely removed by the LEMming approach.

### Distribution of *C*_*t*_-values of reference genes

**Fig 7:**
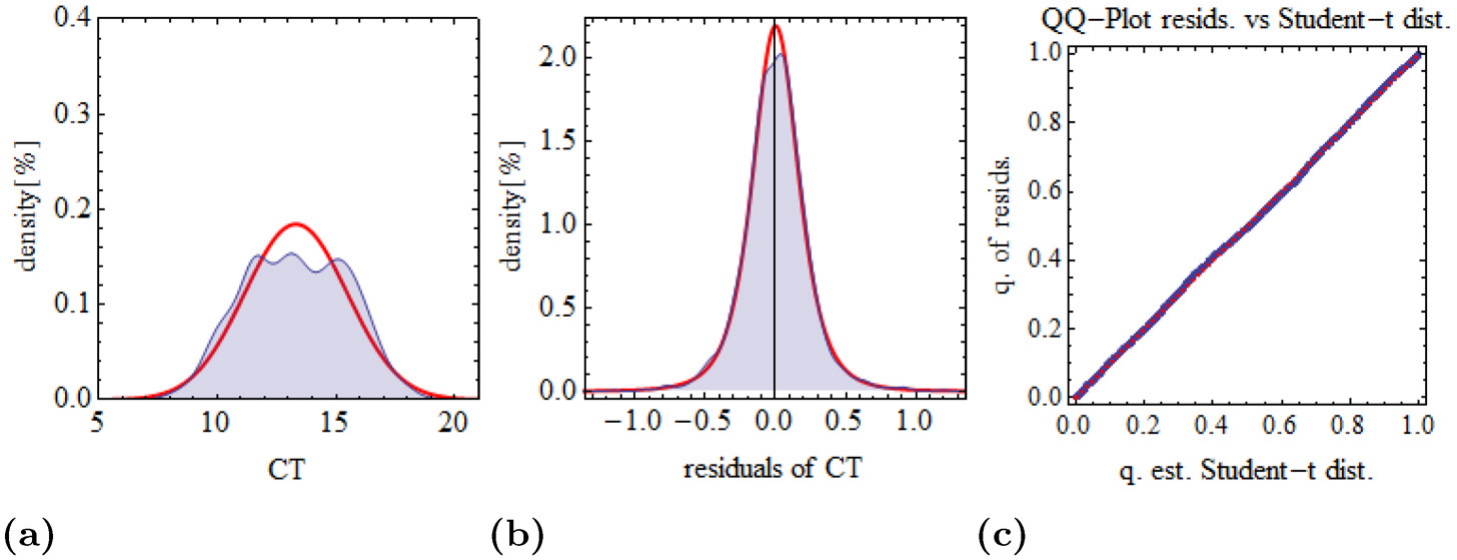
Distribution of raw data and residuals in reference genes in DS3. Density plot of raw data (a) and residuals of LEM-method (b) of reference genes in DS3. Blue: kernel density estimation of raw data/residuals. Red: estimated Student-t distribution. (c) Quantile-Quantile plot with quantiles of estimated Student-t distribution versus quantiles of residuals.

The residuals *ϵ* of LEMming can be described by a Student-t distribution. Distribution tests (*H*_0_ hypothesis (Student-t distribution of residuals)) for DS1 accepted the *H*_0_ hypothesis (Kolmogorov-Smirnov test *p* = 0.228 and Kuiper test *p* = 0.096). Fig 7 shows the distribution of raw data and LEMming residuals for reference genes in DS3. The *H*_0_ hypothesis (Student-t distribution of residuals) was accepted by a Kolmogorov-Smirnov test (*p* = 0.723) and a Kuiper test (*p* = 0.397). The QQ-Plot Fig 7(c) shows the quantiles of the estimated Student-t distribution on the angle bisector with the quantiles of the residuals.

## Discussion

Here we generated and validated a method for normalization of parallel qPCR measurements, called LEMming, which is based on a linear error model to exclude technical and systematic errors. LEMming takes advantage of the experimental design of qPCR studies conducted on microfluidic arrays, which is a high throughput platform for qPCR. Our LEMming tool allows, therefore, the analysis of such data without usage of reference genes (RGs). We applied LEMming to three data sets with different stability patterns of common RGs.

DS1 represents a data set with stable RGs available. *geNorm* and LEMming resulted in nearly the same results. The slight shift in the mean value of 5 genes in *geNorm* processed data could be neglected since the RGs themselves were affected. We concluded that in DS1 *geNorm* and LEMming are on a par in reduction of standard deviation per gene and treatment group. Both methods were superior to the use of a single RG with the ΔΔ*C*_*t*_ method.

DS2 evaluated mice under starvation conditions in liver samples. It represents a data set where stability of RGs is questionable after normalization. RGs were not significantly differential expressed in raw data and were selected according to *geNorm* criteria. However, after *geNorm* and LEMming normalization none of them was accepted as stably expressed by t-tests. Here the question appears, whether such RGs are appropriate for normalization. *geNorm*-normalized data finally showed an effect of a shifted mean per gene per condition compared to the raw data and LEMming-normalized data. Comparing the decrease of standard deviation from raw data to *geNorm* and to LEMming, the latter was superior. LEMming compensated for errors that are responsible for 83.1% of variance in the raw data for the median gene (see Fig 6). We concluded that the use of RGs is not necessary in this case and would result in slightly distorted results.

DS3 evaluated rats after partial hepatectomy under vaso-active drugs in different zones of the liver after various observation times with a control of untreated and ligation of the right median hepatic vein. Thus, DS3 presents a very complex *in vivo* experiment. According to *geNorm* criteria (M-values and pairwise variation) stable RGs existed, while t-tests between treatment conditions in raw data suggested that this is not the case. Even after exclusion of systematic errors by LEMming no stable RGs existed following this t-test criteria. Applying RGs for normalization shifted the mean value per gene under most treatment conditions, which distorts the data analysis. The case of the marker gene *Pcna*, which is upregulated after 24 h and 48 h of partial hepatectomy (PH), demonstrated that raw data and LEMming are in accordance with microarray data. *geNorm* makes the opposite claim of down regulation. Interestingly, Assy et al [26] found the protein PCNA as a marker for PH, but found no increase at the mRNA level using RT-qPCR and normalizing with RGs. It might be, that this occurred due data distortion by the use of RGs.

This rises the question, whether M-values and pairwise variation criteria for stable RG selection are complete. The criteria are based on inter- and intragroup variation of the RGs. The selected RGs *Rpl27*, *Rps13* and *Rpl32* showed similar expression patterns (see Fig 3). If RGs are coexpressed but are not independent of the experimental condition the stability criteria fail. However, to renounce the use of RGs in those complex *in-vivo* experiments without stable RGs following the t-test, LEMming is an alternative for experiment analysis investigated with parallel qPCR.

The analysis of variance in DS1 and DS2 revealed that the reverse transcription step (cDNA conversion error) is the dominant technical error. Treatment conditions had a positive effect on the biological variance compared to the untreated condition. LEMming was able to remove large proportions of technical errors and retained the biological variation. We showed theoretically and practically that applying LEMming results in reduced gene wise variances per treatment group compared to normalization with a single RG. The reduction of these variances is based on the removal of systematic errors which are part of a linear mixed model estimated from the data. It is important to estimate the effects of this model in a particular order, otherwise effects like the global treatment effect would be removed as a sample error. Usually the residuals of linear mixed models are assumed to be normally distributed. If this is not the case, estimated parameters might be biased. We observed a Student t-distribution of residuals of the linear mixed model. This distribution is symmetric like a normal distribution, but has heavier tails. Thus, the estimated effects are not biased, but the standard error of the estimated effects might be inaccurate. To not overestimate the significances of differential expressions, we recommend to use robust tests to analyze them. We used the function *ebayes* with option robust from the *R* package *limma* [25] in order to do that.

LEMming uses the assumption that the means of *C*_*t*_ values within the samples of similarly treated groups are equal. Since the genes are selected by the criterion to see a difference between conditions, a global treatment effect Δ_*T*_ can be shown in most data sets. However, a systematic sample error per treated group $\tilde{\epsilon }$ and the global treatment effect Δ_*T*_ are indistinguishable by LEMming. The use of RGs which are provably independent of the treatment would automatically compensate this. If such RGs are available, we strongly recommend to use them because of their capability to automatically remove systematic batch effects. However, with a growing number of experimental conditions the chance of finding such RGs decreases.

Thus, we recommend to use quantification of total cDNA content per sample as a second independent measurement in order to identify systematic sample errors per treated group ($\tilde{\epsilon }$). We exemplified and discussed this issue in detail in S2 File and in S4 File. The usage of total cDNA quantification helps to ensure that the global treatment effect (Δ_*T*_) is not distorted.

Despite a method being independent of RGs, we would still recommend to measure at least two RGs which is recommended according to the MIQE guidelines [2]. We see LEMming as a tool which could complement current qPCR data analysis software by an RG independent normalization approach.

## Supporting Information

S1 File Result of data set 1 and proof of decreased error variances. (PDF)

S2 File Derivation of LEMming error model and results of data set 2. (PDF)

S3 File R-Script for using LEMming with data set 2. (R)

S4 File Results of data set 3. (PDF)

S1 Table Data set 1. (XLSX)

S2 Table Data set 2. (XLSX)

S3 Table Raw and analyzed data of RGs in data set 3. (XLSX)
