# Supplementary material for: LEMming: A Linear Error Model to Normalize Parallel Quantitative Real-Time PCR (qPCR) Data as an Alternative to Reference Gene Based Methods
Source: PLoS One. 2015 Sep 1;10(9):e0135852. doi: 10.1371/journal.pone.0135852 (PMC4556681; doi:10.1371/journal.pone.0135852)
Supplement: S1 File — (PDF) [file pone.0135852.s001.pdf]

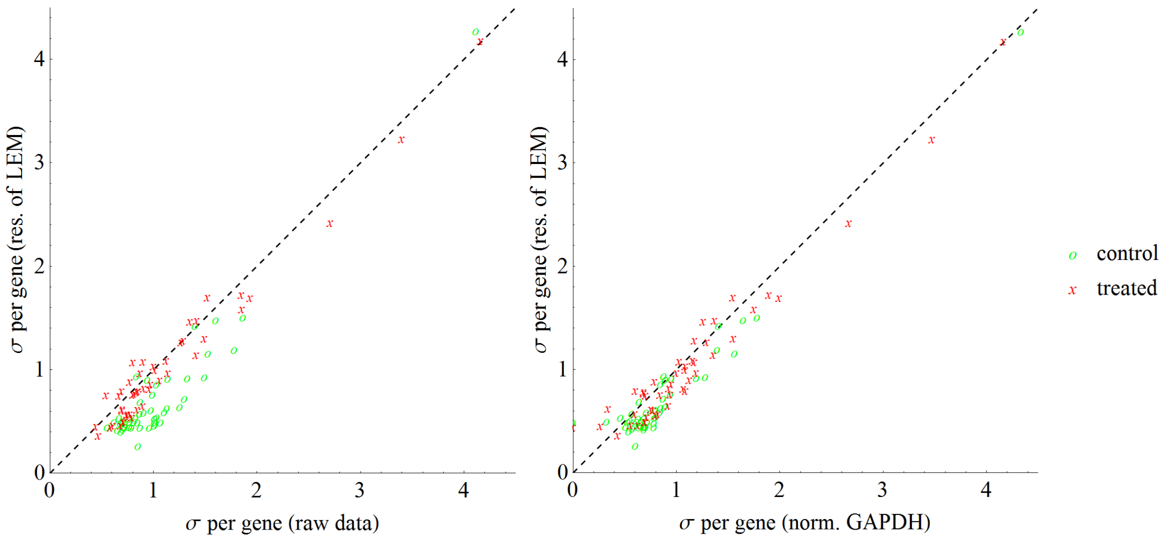

Figure 1: Comparision of standard deviation σ per gene for data set 1. Left: Raw data versus LEMming. Right: $\Delta\Delta CT$ normalized data (reference gene: *Gapdh*) versus LEMming processed data. Both cases show that σ's per gene of LEMming processed are smaller.

Error variance of $\Delta\Delta C_t$ method in comparison with LEMming

Here, we want to proof, that LEMming results in a decreased standard deviation compared $\Delta\Delta C_t$ with one reference gene (RG). In the following we use abbreviations for *ref* - r, *target* - t, *sample* - s and *control* - c. For sake of simplicity we make three simplifying assumptions: We assume that the PCR efficiency $E_t = E_r$, the $\Delta\Delta C_t$ is

$$\Delta\Delta C_t = (C_t^{r,s} - C_t^{t,s}) - (C_t^{r,c} - C_t^{t,c})$$

We assume that control and sample measurements originate from different animals. According to LEMming the C_t-values can be decomposed (e.g. $C_t^{r,s} = \epsilon_{P:A,r} + \epsilon_{S,s} + \Delta_{T,s} + \Delta_{T:G,r,s} + \epsilon_{r,s}$). Calculating the $\Delta\Delta C_t$ value the probe and sample errors cancel out. The variables $\Delta_{T,\_}$ and $\Delta_{T:G,\_,\_}$ with the according indices are not classified as random variables. Thus, their variance is zero. The random variables $\epsilon_{r,s}$, $\epsilon_{r,c}$, $\epsilon_{t,s}$ and $\epsilon_{t,c}$ are independently distributed.

$$Var(\Delta\Delta C_t) = Var(\epsilon_{r,s} - \epsilon_{t,s} - \epsilon_{r,c} + \epsilon_{t,c})$$

Assuming that all ϵ's have the same variance σ_ϵ^2, the variance of $\Delta\Delta C_t$ is given by $4\sigma_\epsilon^2$.
The ΔC_t (distance to the mean of the control) of LEMming has the variance

$$Var(\Delta C_t) = Var(\epsilon_{t,s}) + Var(Mean(\epsilon_{t,c}))$$

With all ϵ's being equal the variance is of the ΔC_t is $\sigma_\epsilon^2 + \frac{\sigma_\epsilon^2}{n}$ where n is the size of the control group. This means that $Var(\Delta C_t) < Var(\Delta\Delta C_t)$, proving that LEMming has lower variance compared to the $\Delta\Delta C_t$ method. If the variances of control group and treated group are different, as we have shown for DS1 in the main manuscript, only $Var(\epsilon_{t,s})$ cancel out and the variances are:

$$Var(Mean(\epsilon_{t,c})) < Var(\epsilon_{r,s}) + Var(\epsilon_{r,c}) + Var(\epsilon_{t,c}).$$

If sample and control are from the same animals, the random variables ϵ are not statistically independent. The covariance of ϵ's can reduce the variances of the $\Delta\Delta C_t$ method.

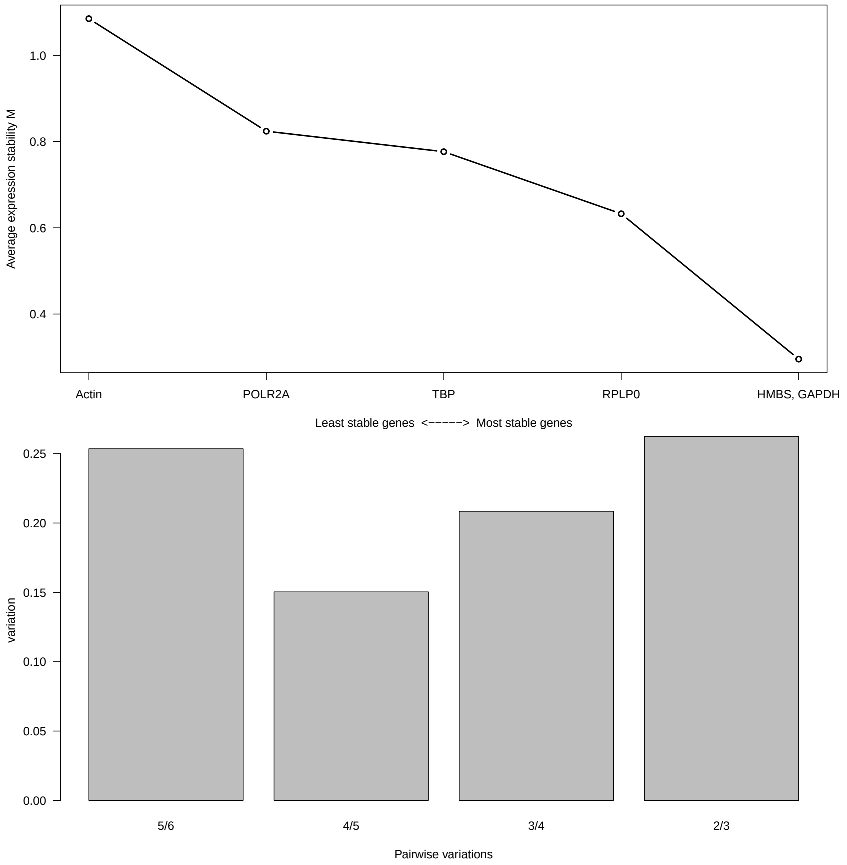

Figure 2: Average expression stability and pairwise variation of data set 1 according to Vandesompele et al. 2002. We selected *GAPDH, HMBS, RPLP0* and *TBP* with a pairwise variation of 0.15 and M-values below 0.8 as RGs for *geNorm* normalization.

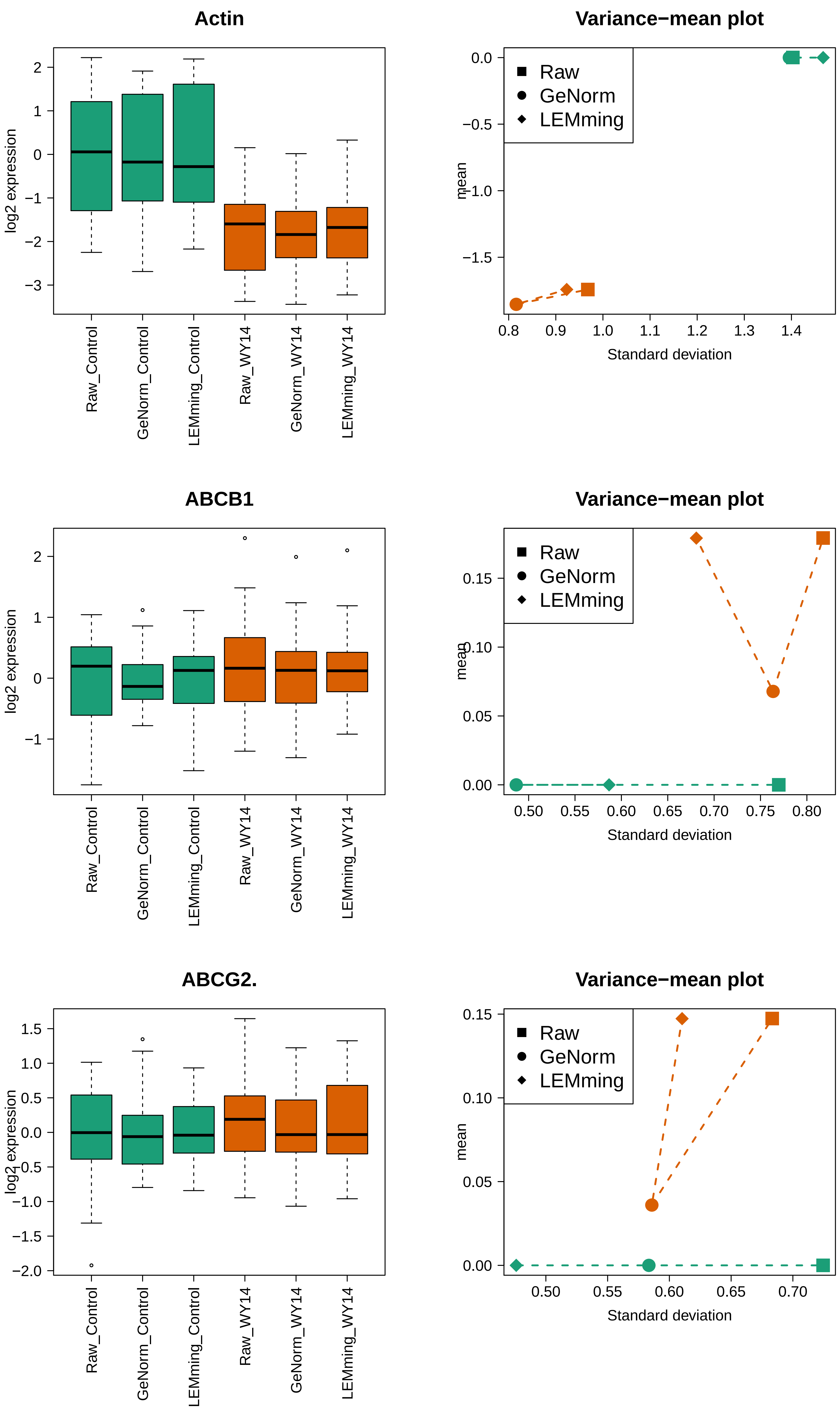
Actin
log2 expression
Raw_Control
GeNorm_Control
LEMming_Control
Raw_WY14
GeNorm_WY14
LEMming_WY14
Variance−mean plot
Raw
GeNorm
LEMming
mean
Standard deviation
ABCB1
log2 expression
Raw_Control
GeNorm_Control
LEMming_Control
Raw_WY14
GeNorm_WY14
LEMming_WY14
Variance−mean plot
Raw
GeNorm
LEMming
mean
Standard deviation
ABCG2.
log2 expression
Raw_Control
GeNorm_Control
LEMming_Control
Raw_WY14
GeNorm_WY14
LEMming_WY14
Variance−mean plot
Raw
GeNorm
LEMming
mean
Standard deviation

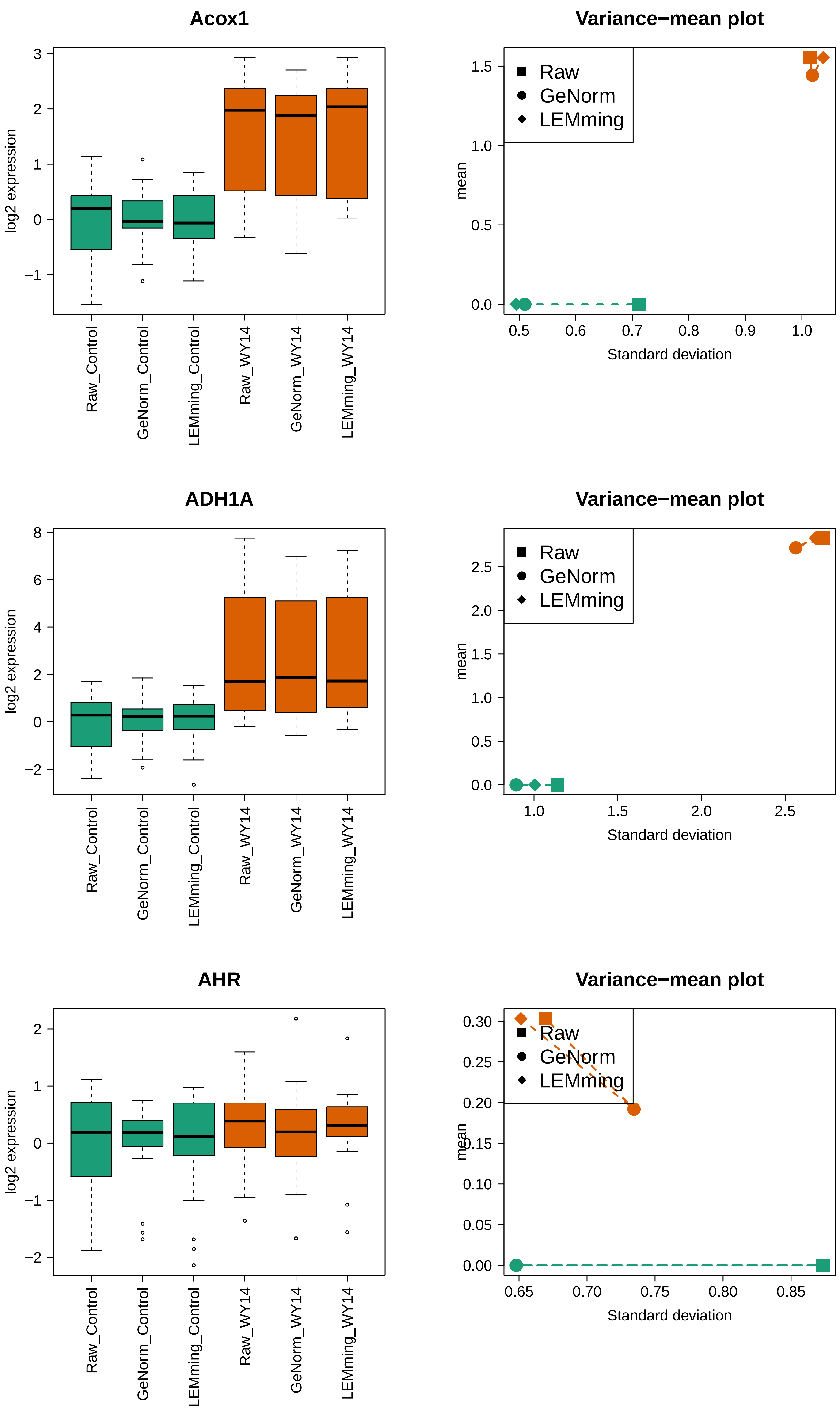
Acox1
Variance−mean plot
ADH1A
Variance−mean plot
AHR
Variance−mean plot
log2 expression
mean
Standard deviation
Raw
GeNorm
LEMming
Raw_Control
GeNorm_Control
LEMming_Control
Raw_WY14
GeNorm_WY14
LEMming_WY14

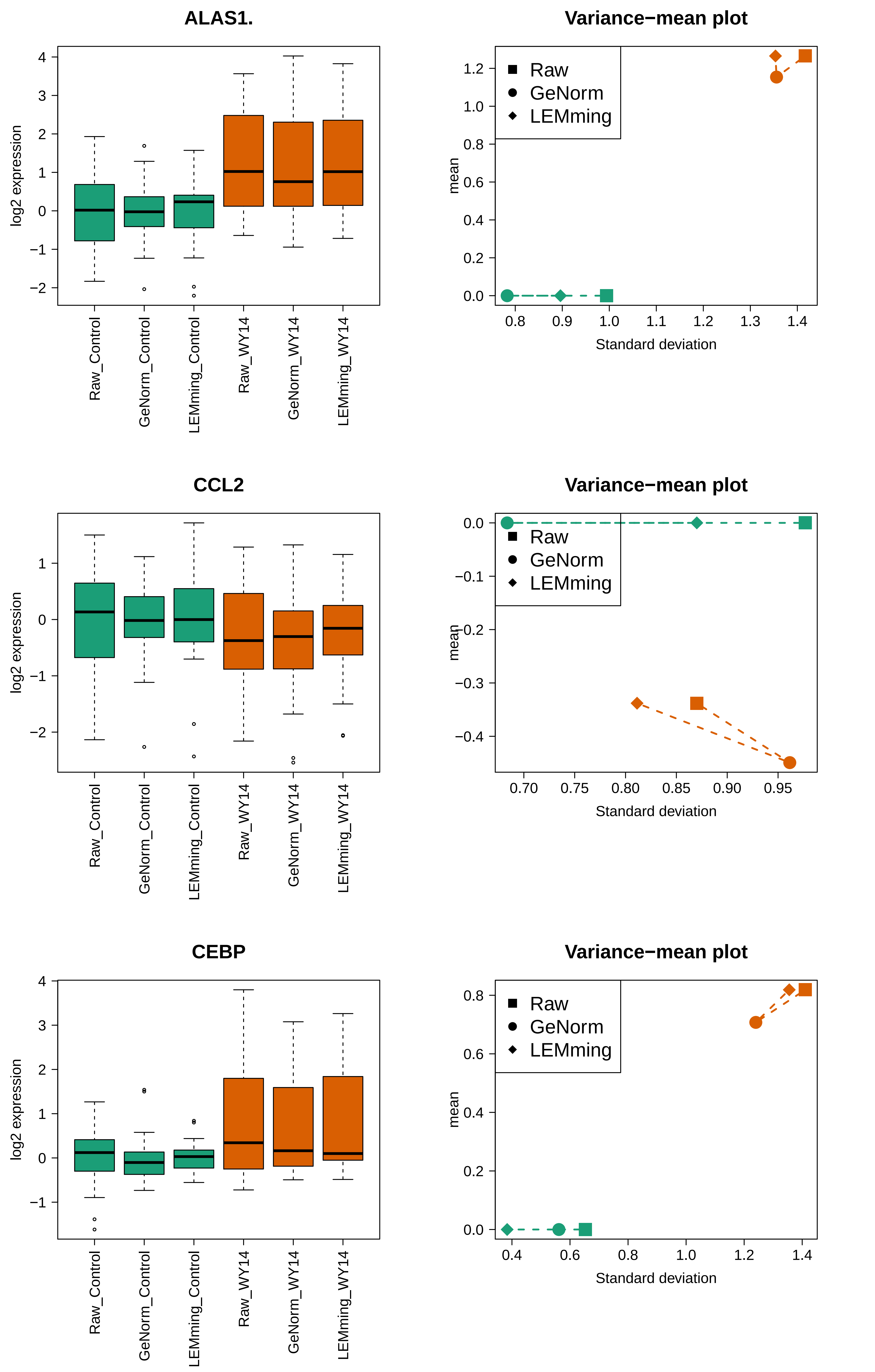
ALAS1.
log2 expression
Raw_Control
GeNorm_Control
LEMming_Control
Raw_WY14
GeNorm_WY14
LEMming_WY14
Variance−mean plot
Raw
GeNorm
LEMming
mean
Standard deviation
CCL2
log2 expression
Raw_Control
GeNorm_Control
LEMming_Control
Raw_WY14
GeNorm_WY14
LEMming_WY14
Variance−mean plot
Raw
GeNorm
LEMming
mean
Standard deviation
CEBP
log2 expression
Raw_Control
GeNorm_Control
LEMming_Control
Raw_WY14
GeNorm_WY14
LEMming_WY14
Variance−mean plot
Raw
GeNorm
LEMming
mean
Standard deviation

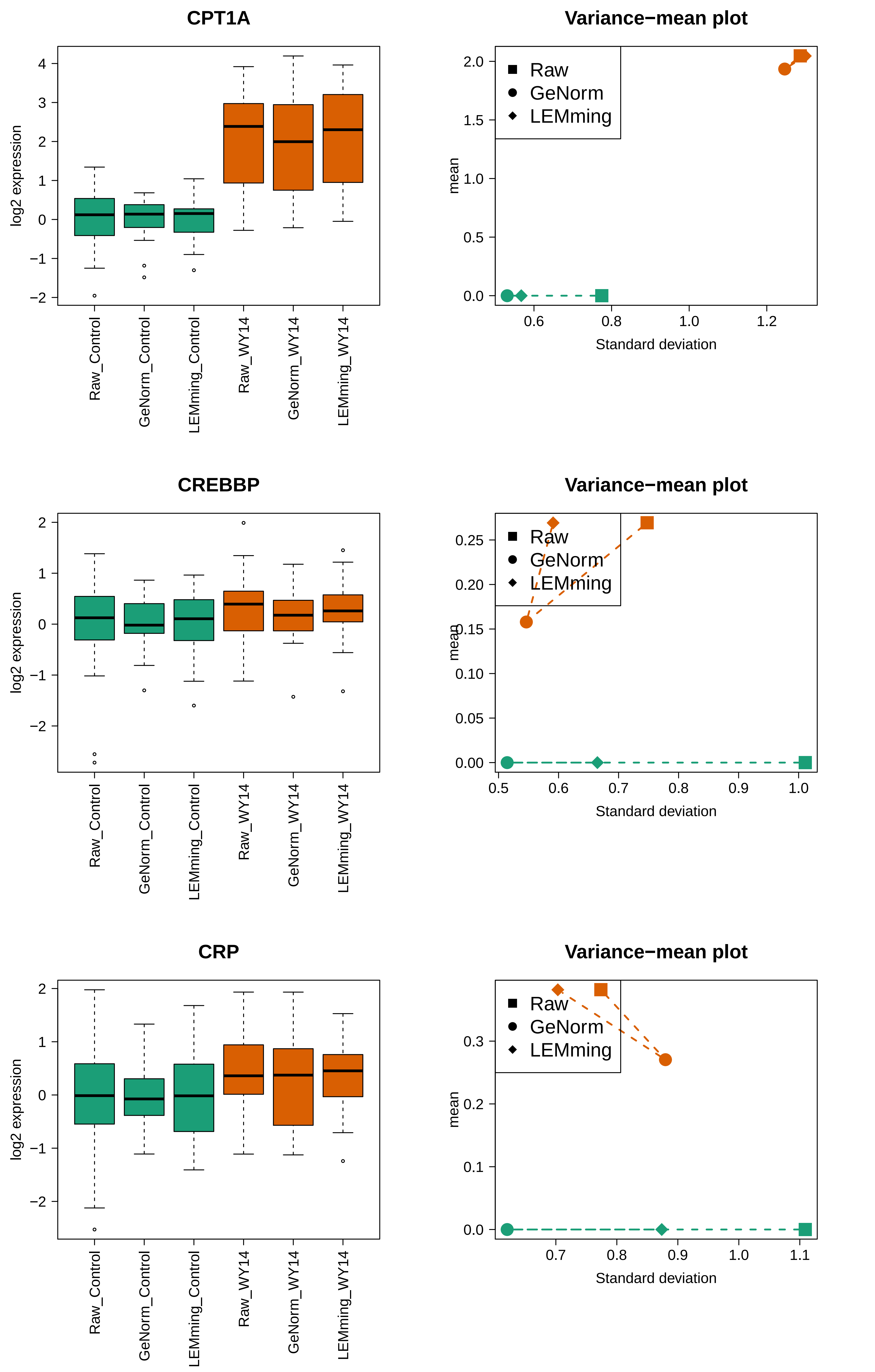
CPT1A
Variance−mean plot
CREBBP
Variance−mean plot
CRP
Variance−mean plot
log2 expression
Raw_Control
GeNorm_Control
LEMming_Control
Raw_WY14
GeNorm_WY14
LEMming_WY14
mean
Standard deviation
Raw
GeNorm
LEMming

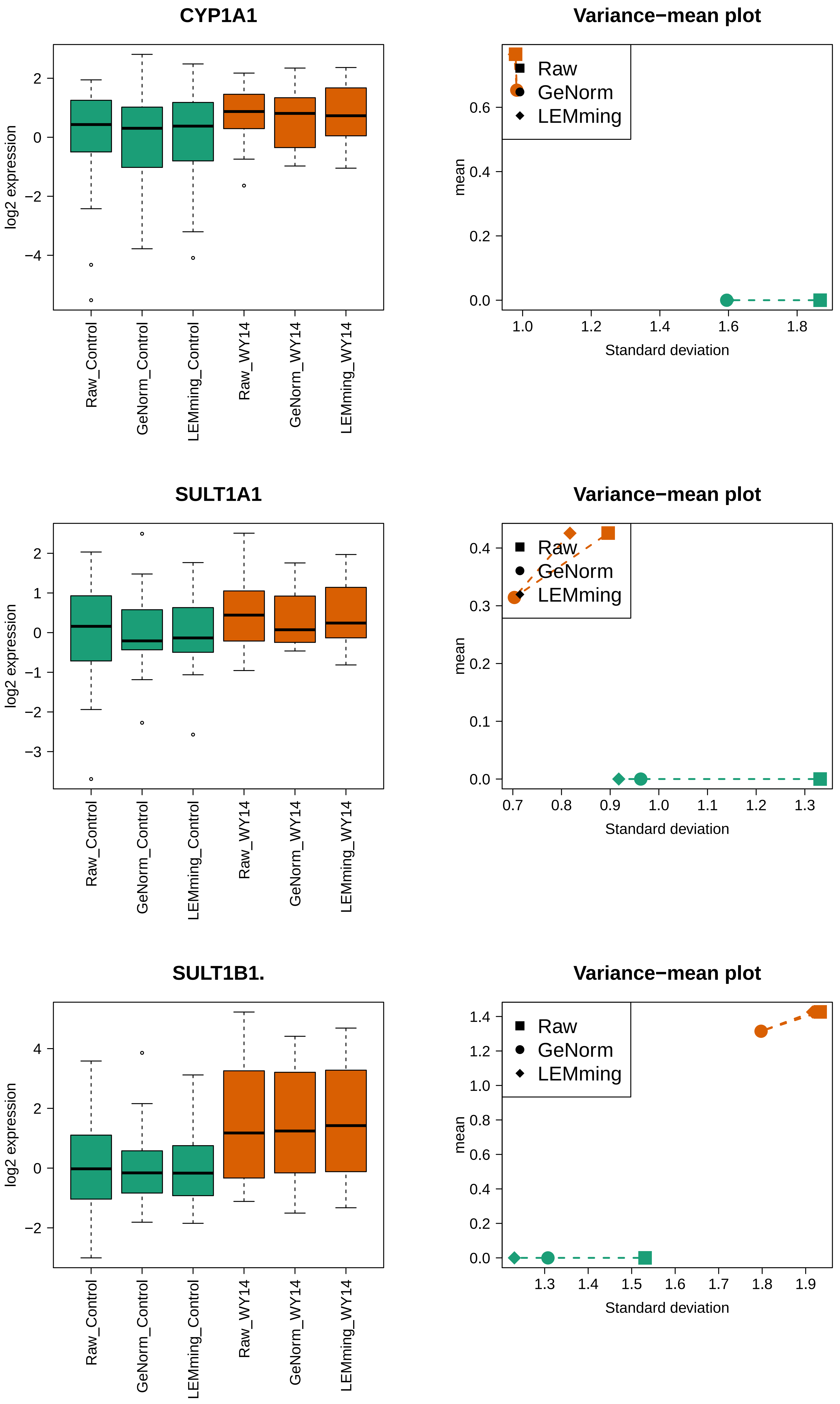

CYP1A1
log2 expression
Raw_Control
GeNorm_Control
LEMming_Control
Raw_WY14
GeNorm_WY14
LEMming_WY14
Variance−mean plot
Raw
GeNorm
LEMming
mean
Standard deviation
SULT1A1
log2 expression
Raw_Control
GeNorm_Control
LEMming_Control
Raw_WY14
GeNorm_WY14
LEMming_WY14
Variance−mean plot
Raw
GeNorm
LEMming
mean
Standard deviation
SULT1B1.
log2 expression
Raw_Control
GeNorm_Control
LEMming_Control
Raw_WY14
GeNorm_WY14
LEMming_WY14
Variance−mean plot
Raw
GeNorm
LEMming
mean
Standard deviation

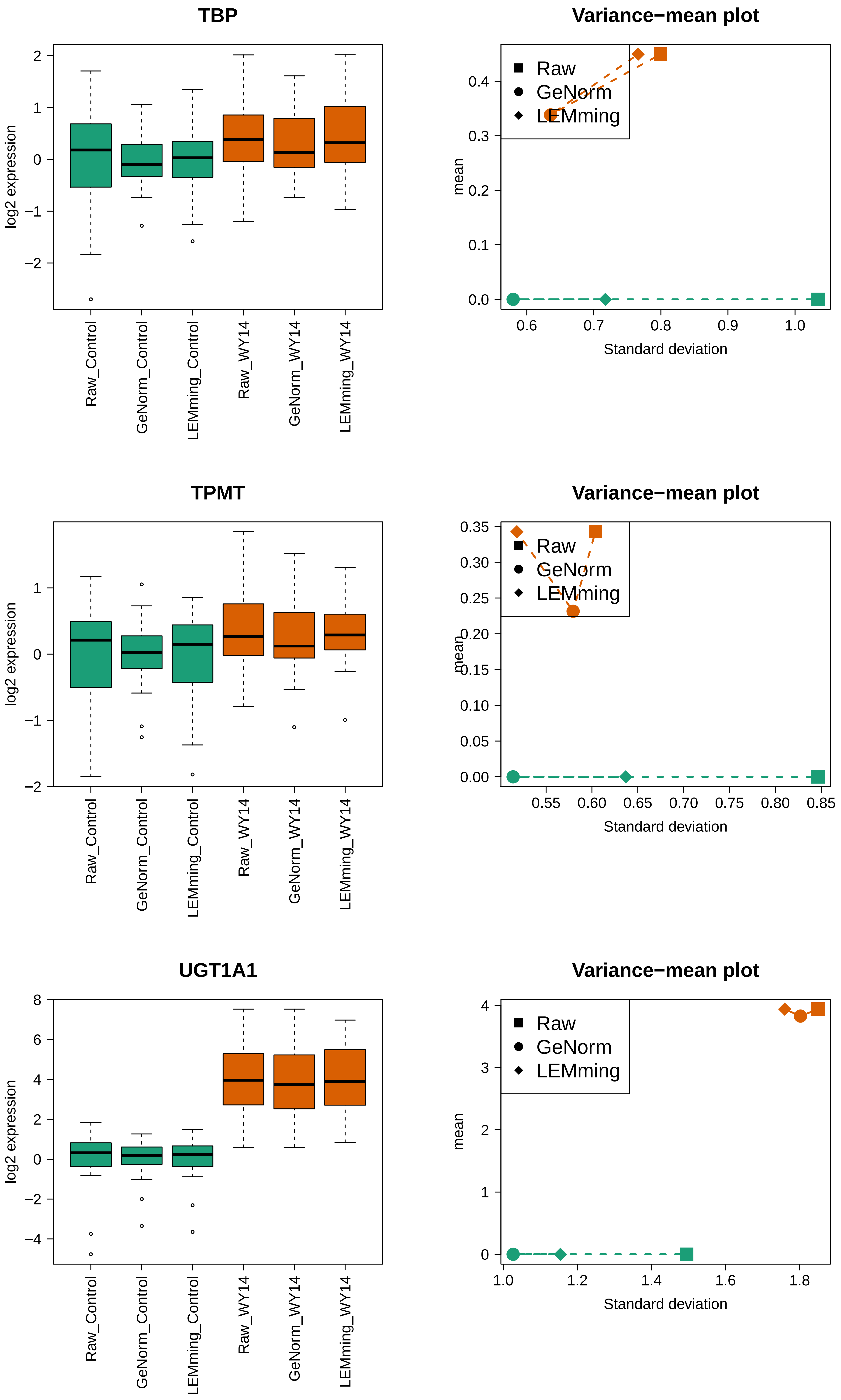

TBP
log2 expression
Raw_Control
GeNorm_Control
LEMming_Control
Raw_WY14
GeNorm_WY14
LEMming_WY14
Variance−mean plot
Raw
GeNorm
LEMming
mean
Standard deviation
TPMT
log2 expression
Raw_Control
GeNorm_Control
LEMming_Control
Raw_WY14
GeNorm_WY14
LEMming_WY14
Variance−mean plot
Raw
GeNorm
LEMming
mean
Standard deviation
UGT1A1
log2 expression
Raw_Control
GeNorm_Control
LEMming_Control
Raw_WY14
GeNorm_WY14
LEMming_WY14
Variance−mean plot
Raw
GeNorm
LEMming
mean
Standard deviation

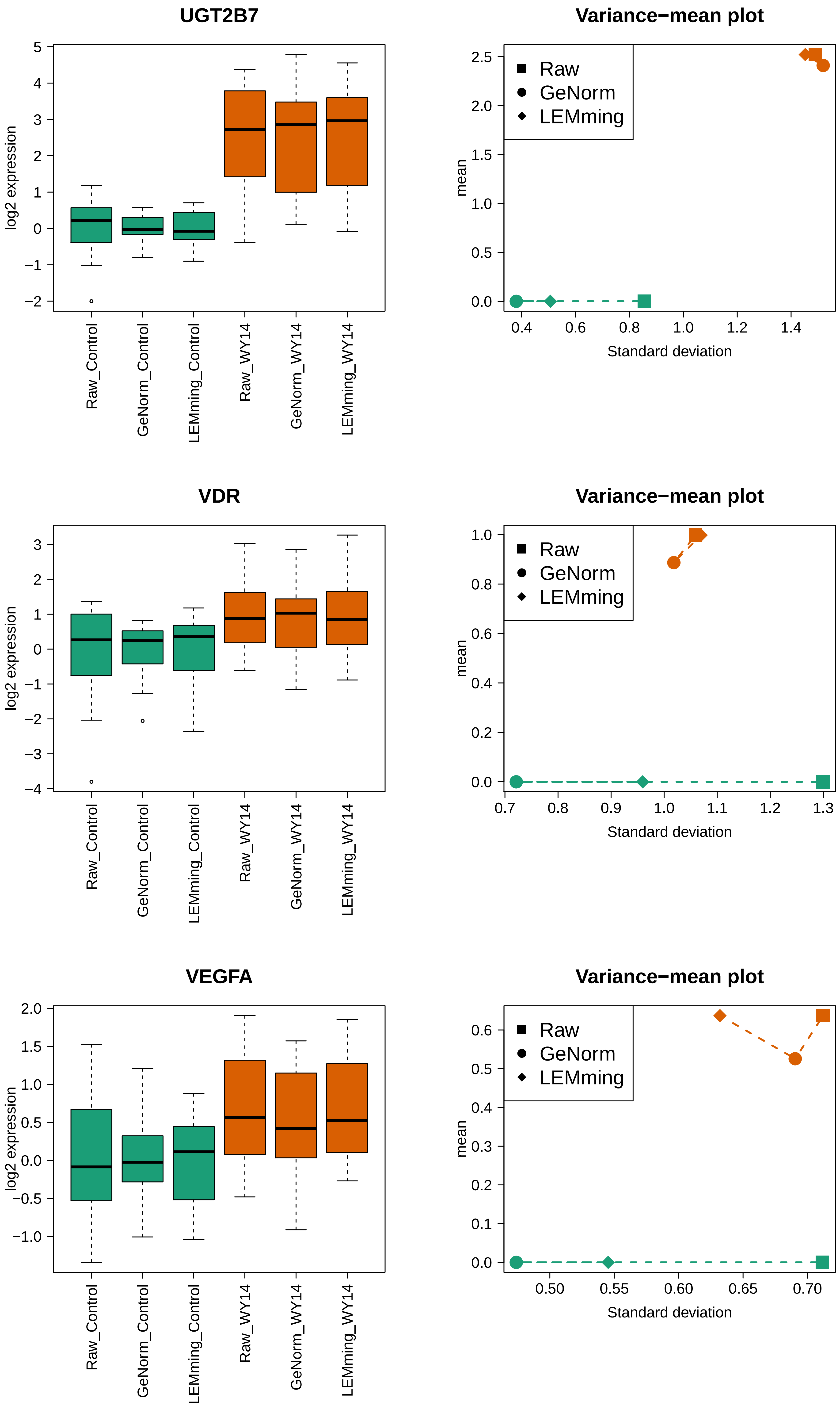
UGT2B7
log2 expression
5
4
3
2
1
0
−1
−2
Raw_Control
GeNorm_Control
LEMming_Control
Raw_WY14
GeNorm_WY14
LEMming_WY14
Variance−mean plot
Raw
GeNorm
LEMming
mean
2.5
2.0
1.5
1.0
0.5
0.0
0.4
0.6
0.8
1.0
1.2
1.4
Standard deviation
VDR
log2 expression
3
2
1
0
−1
−2
−3
−4
Raw_Control
GeNorm_Control
LEMming_Control
Raw_WY14
GeNorm_WY14
LEMming_WY14
Variance−mean plot
Raw
GeNorm
LEMming
mean
1.0
0.8
0.6
0.4
0.2
0.0
0.7
0.8
0.9
1.0
1.1
1.2
1.3
Standard deviation
VEGFA
log2 expression
2.0
1.5
1.0
0.5
0.0
−0.5
−1.0
Raw_Control
GeNorm_Control
LEMming_Control
Raw_WY14
GeNorm_WY14
LEMming_WY14
Variance−mean plot
Raw
GeNorm
LEMming
mean
0.6
0.5
0.4
0.3
0.2
0.1
0.0
0.50
0.55
0.60
0.65
0.70
Standard deviation

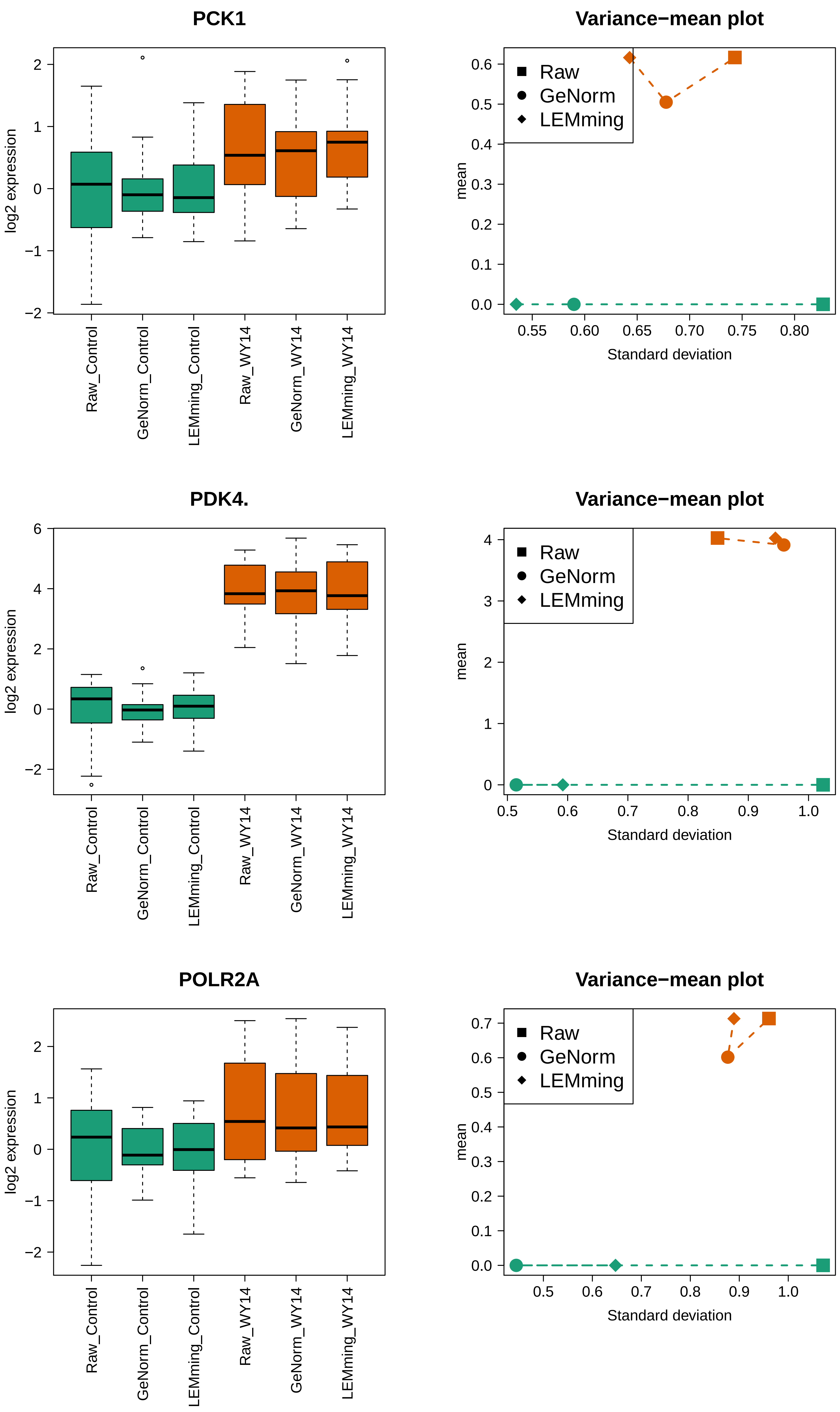
PCK1
Variance−mean plot
log2 expression
Raw_Control
GeNorm_Control
LEMming_Control
Raw_WY14
GeNorm_WY14
LEMming_WY14
Raw
GeNorm
LEMming
mean
Standard deviation
PDK4.
Variance−mean plot
log2 expression
Raw_Control
GeNorm_Control
LEMming_Control
Raw_WY14
GeNorm_WY14
LEMming_WY14
Raw
GeNorm
LEMming
mean
Standard deviation
POLR2A
Variance−mean plot
log2 expression
Raw_Control
GeNorm_Control
LEMming_Control
Raw_WY14
GeNorm_WY14
LEMming_WY14
Raw
GeNorm
LEMming
mean
Standard deviation

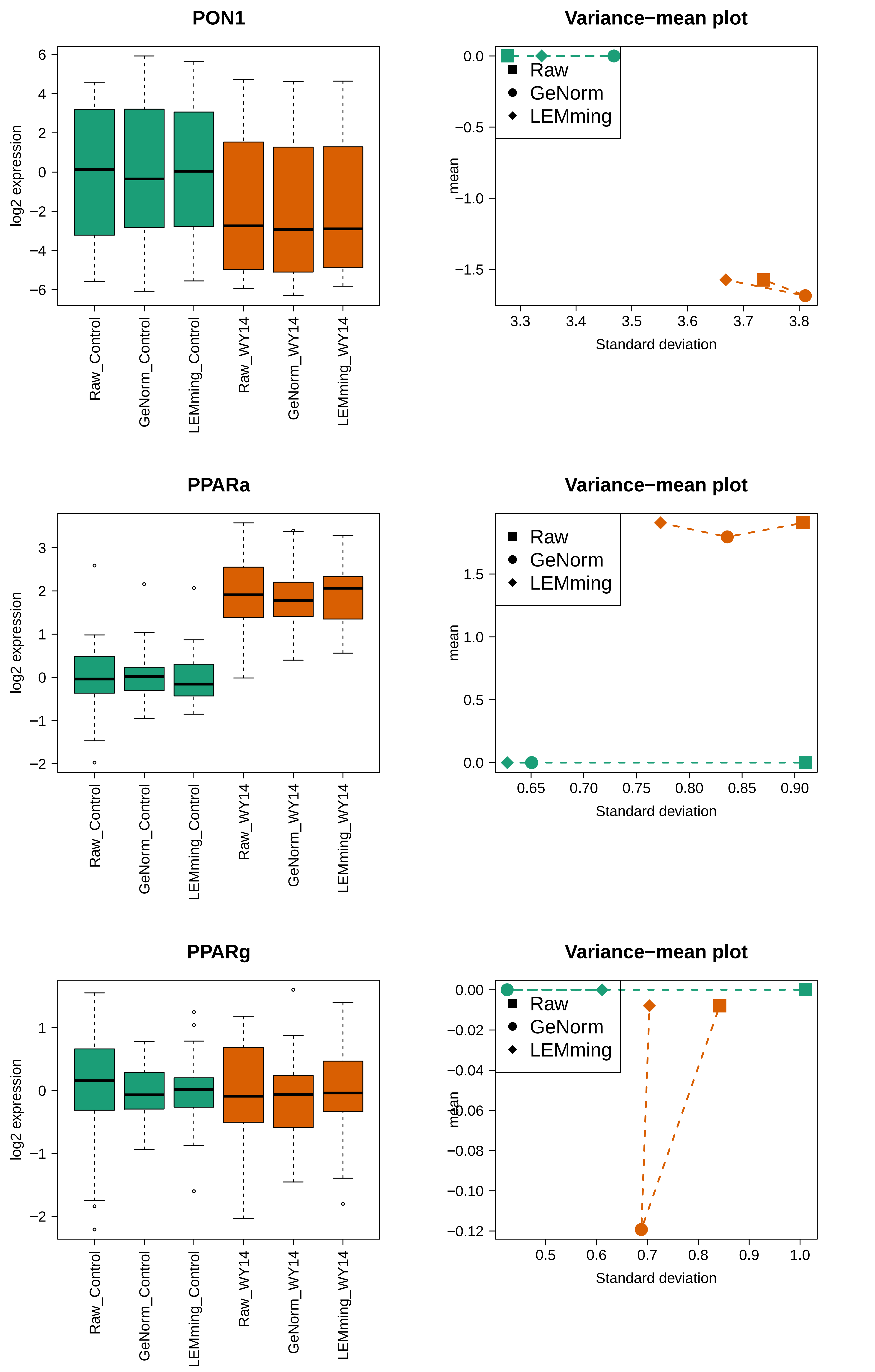
PON1
log2 expression
6
4
2
0
−2
−4
−6
Raw_Control
GeNorm_Control
LEMming_Control
Raw_WY14
GeNorm_WY14
LEMming_WY14
Variance−mean plot
mean
0.0
−0.5
−1.0
−1.5
3.3
3.4
3.5
3.6
3.7
3.8
Standard deviation
Raw
GeNorm
LEMming
PPARa
log2 expression
3
2
1
0
−1
−2
Raw_Control
GeNorm_Control
LEMming_Control
Raw_WY14
GeNorm_WY14
LEMming_WY14
Variance−mean plot
mean
1.5
1.0
0.5
0.0
0.65
0.70
0.75
0.80
0.85
0.90
Standard deviation
Raw
GeNorm
LEMming
PPARg
log2 expression
1
0
−1
−2
Raw_Control
GeNorm_Control
LEMming_Control
Raw_WY14
GeNorm_WY14
LEMming_WY14
Variance−mean plot
mean
0.00
−0.02
−0.04
−0.06
−0.08
−0.10
−0.12
0.5
0.6
0.7
0.8
0.9
1.0
Standard deviation
Raw
GeNorm
LEMming

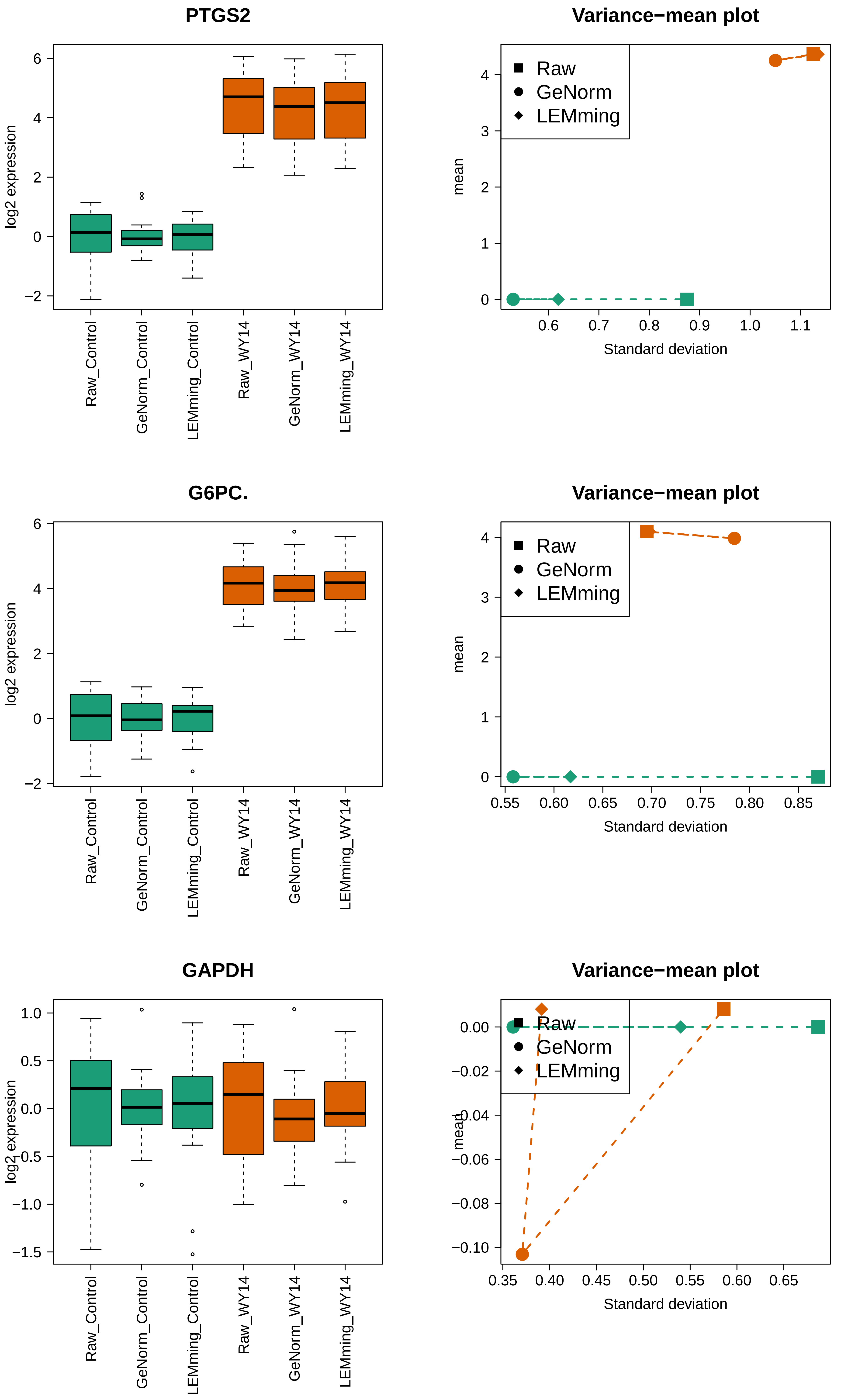
PTGS2
log2 expression
Raw_Control
GeNorm_Control
LEMming_Control
Raw_WY14
GeNorm_WY14
LEMming_WY14
Variance−mean plot
Raw
GeNorm
LEMming
mean
Standard deviation
G6PC.
GAPDH

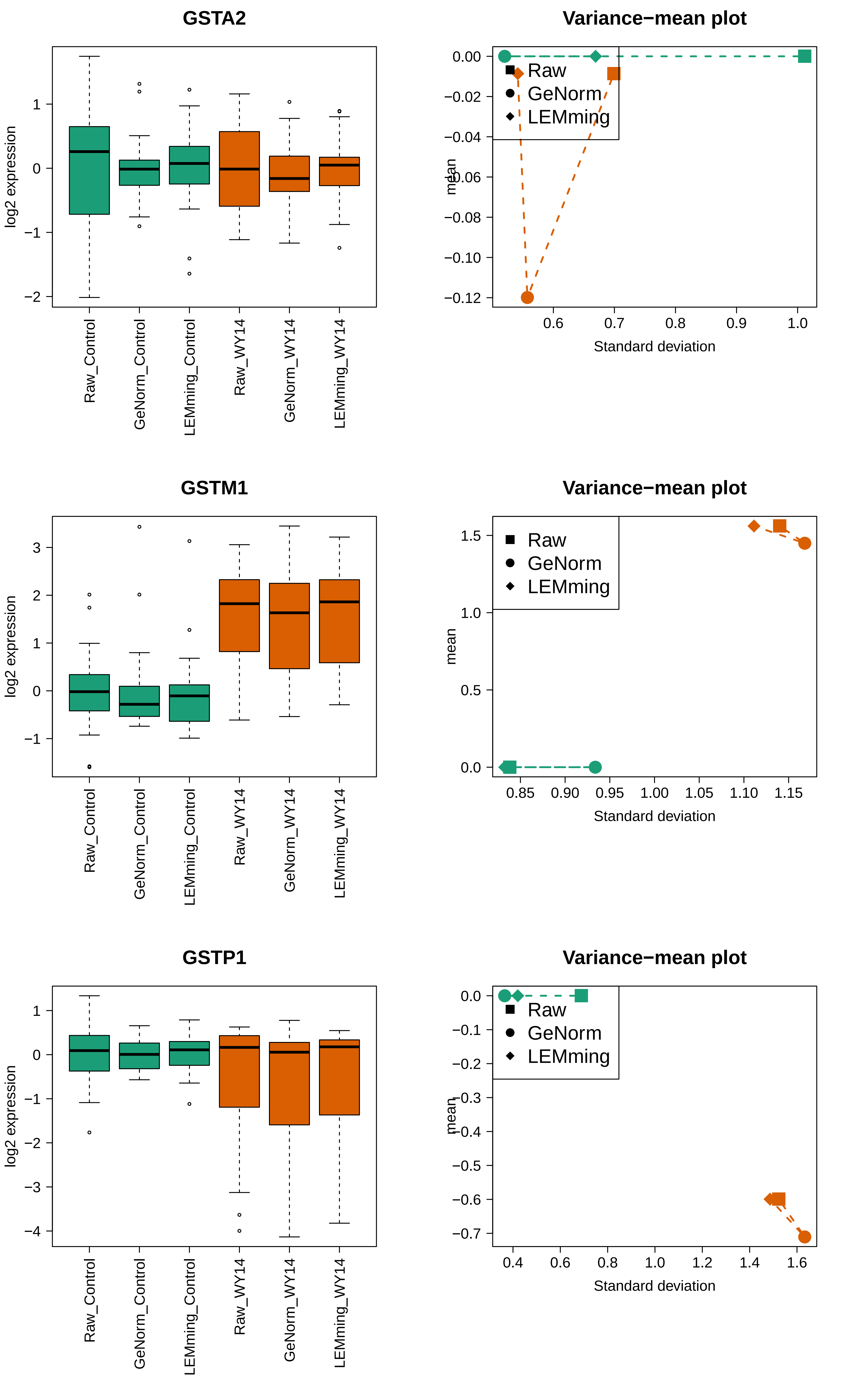

GSTA2
Variance−mean plot
GSTM1
Variance−mean plot
GSTP1
Variance−mean plot
log2 expression
Raw_Control
GeNorm_Control
LEMming_Control
Raw_WY14
GeNorm_WY14
LEMming_WY14
mean
Standard deviation
Raw
GeNorm
LEMming

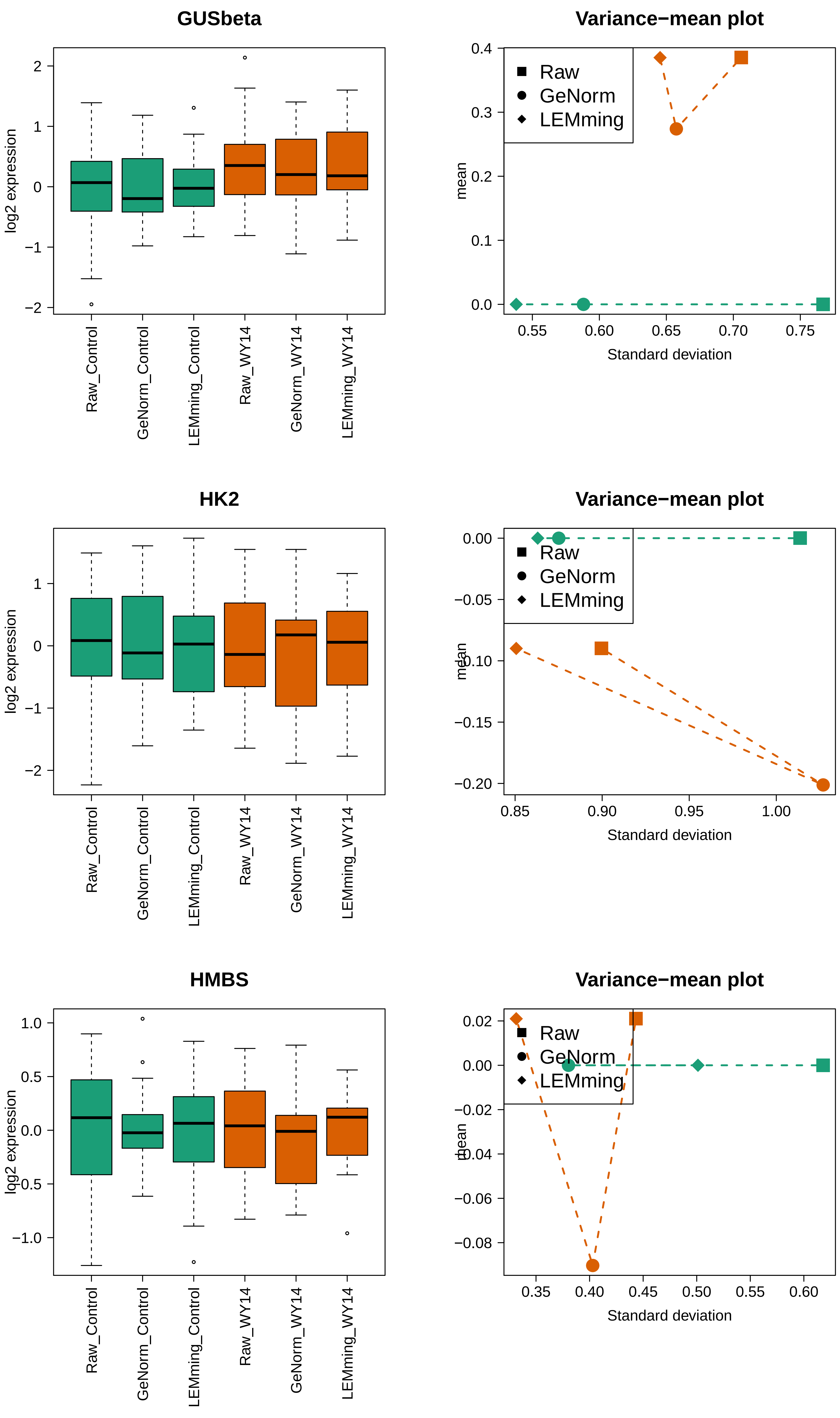
GUSbeta
log2 expression
Raw_Control
GeNorm_Control
LEMming_Control
Raw_WY14
GeNorm_WY14
LEMming_WY14
Variance−mean plot
Raw
GeNorm
LEMming
mean
Standard deviation
HK2
log2 expression
Raw_Control
GeNorm_Control
LEMming_Control
Raw_WY14
GeNorm_WY14
LEMming_WY14
Variance−mean plot
Raw
GeNorm
LEMming
mean
Standard deviation
HMBS
log2 expression
Raw_Control
GeNorm_Control
LEMming_Control
Raw_WY14
GeNorm_WY14
LEMming_WY14
Variance−mean plot
Raw
GeNorm
LEMming
mean
Standard deviation

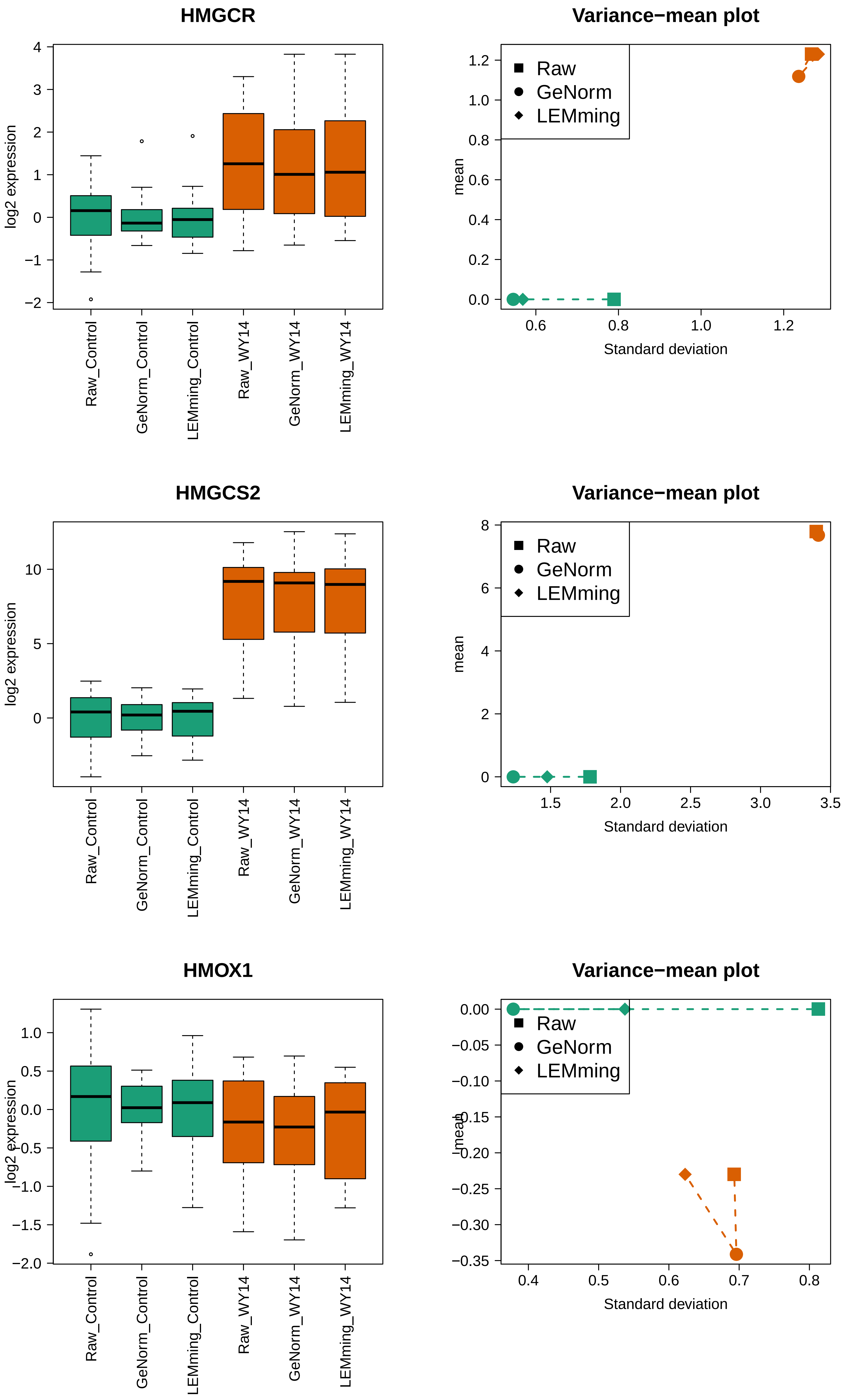
HMGCR
log2 expression
Raw_Control
GeNorm_Control
LEMming_Control
Raw_WY14
GeNorm_WY14
LEMming_WY14
Variance−mean plot
Raw
GeNorm
LEMming
mean
Standard deviation
HMGCS2
log2 expression
Raw_Control
GeNorm_Control
LEMming_Control
Raw_WY14
GeNorm_WY14
LEMming_WY14
Variance−mean plot
Raw
GeNorm
LEMming
mean
Standard deviation
HMOX1
log2 expression
Raw_Control
GeNorm_Control
LEMming_Control
Raw_WY14
GeNorm_WY14
LEMming_WY14
Variance−mean plot
Raw
GeNorm
LEMming
mean
Standard deviation

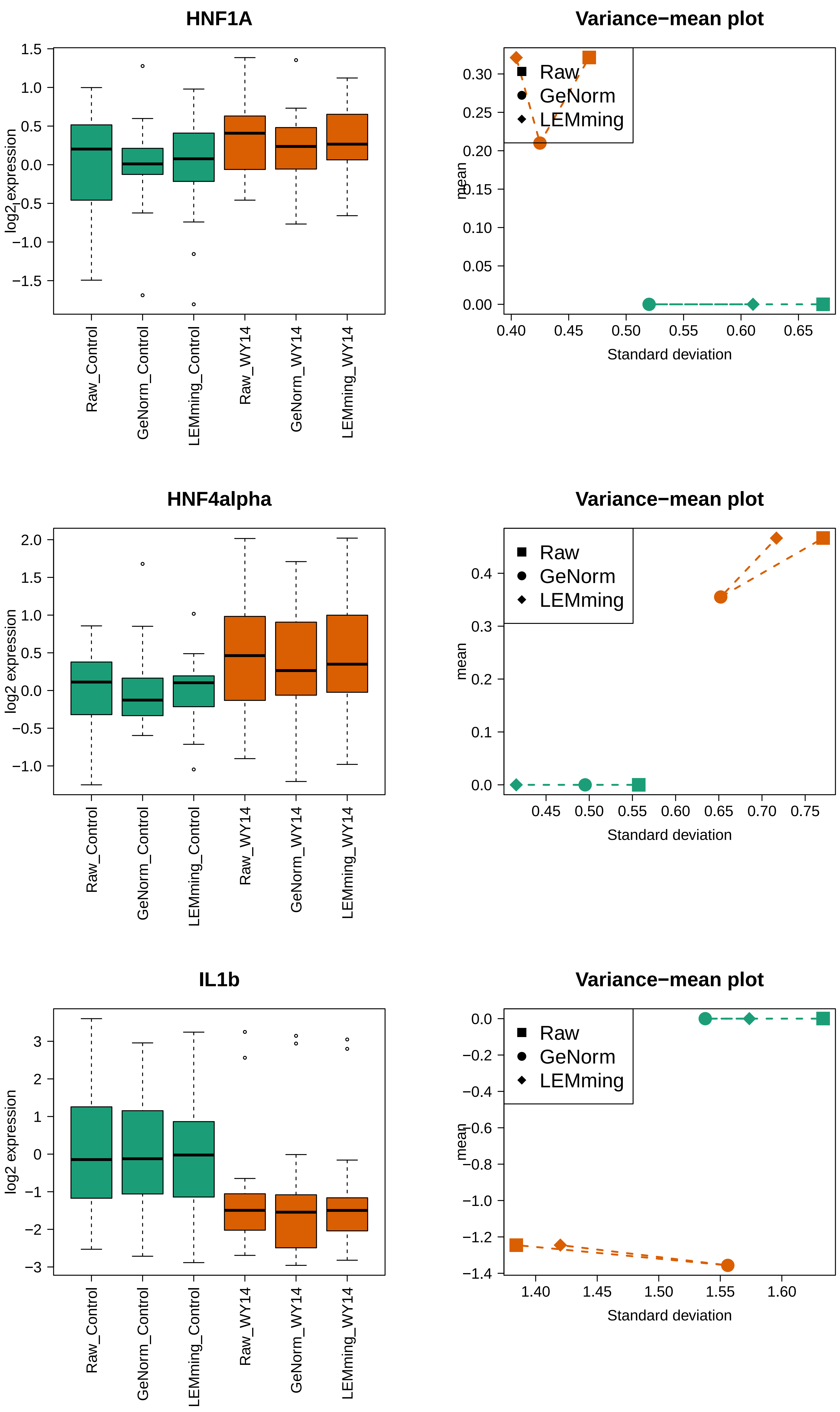
HNF1A
log2 expression
Raw_Control
GeNorm_Control
LEMming_Control
Raw_WY14
GeNorm_WY14
LEMming_WY14
Variance−mean plot
Raw
GeNorm
LEMming
mean
Standard deviation
HNF4alpha
log2 expression
Raw_Control
GeNorm_Control
LEMming_Control
Raw_WY14
GeNorm_WY14
LEMming_WY14
Variance−mean plot
Raw
GeNorm
LEMming
mean
Standard deviation
IL1b
log2 expression
Raw_Control
GeNorm_Control
LEMming_Control
Raw_WY14
GeNorm_WY14
LEMming_WY14
Variance−mean plot
Raw
GeNorm
LEMming
mean
Standard deviation

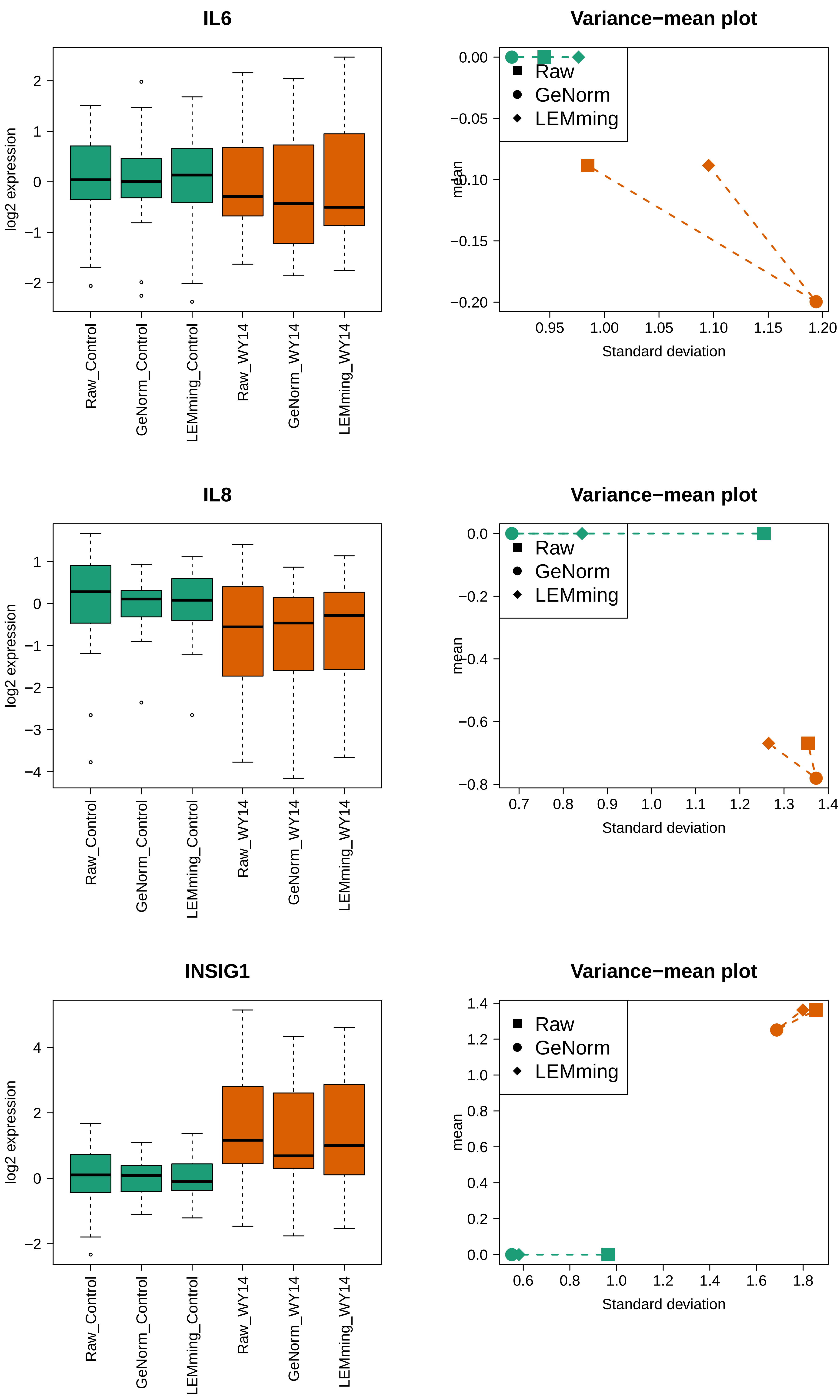

IL6
log2 expression
Raw_Control
GeNorm_Control
LEMming_Control
Raw_WY14
GeNorm_WY14
LEMming_WY14
Variance−mean plot
Raw
GeNorm
LEMming
mean
Standard deviation
IL8
log2 expression
Raw_Control
GeNorm_Control
LEMming_Control
Raw_WY14
GeNorm_WY14
LEMming_WY14
Variance−mean plot
Raw
GeNorm
LEMming
mean
Standard deviation
INSIG1
log2 expression
Raw_Control
GeNorm_Control
LEMming_Control
Raw_WY14
GeNorm_WY14
LEMming_WY14
Variance−mean plot
Raw
GeNorm
LEMming
mean
Standard deviation

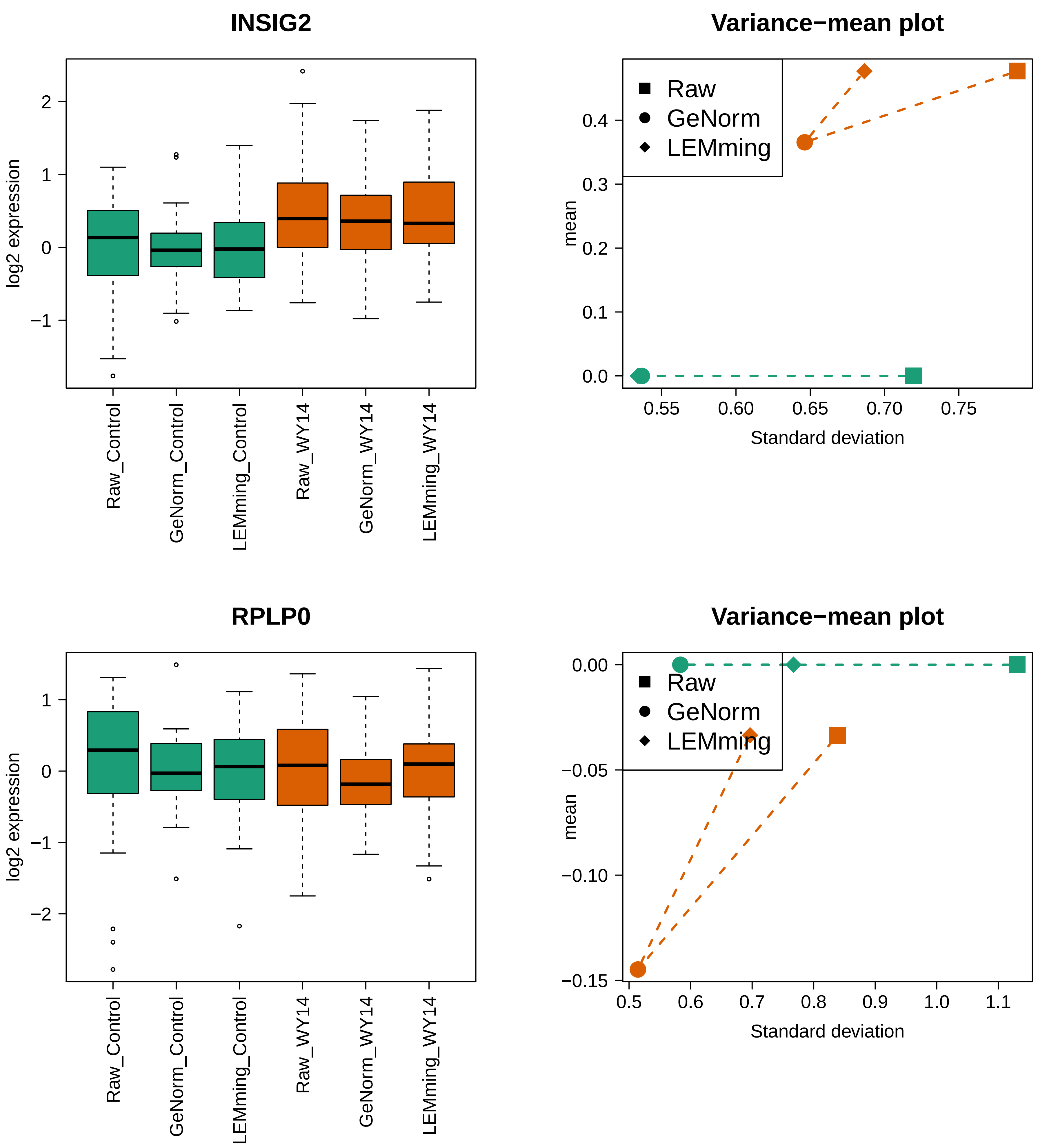

INSIG2
log2 expression
2
1
0
−1
Raw_Control
GeNorm_Control
LEMming_Control
Raw_WY14
GeNorm_WY14
LEMming_WY14
Variance−mean plot
Raw
GeNorm
LEMming
mean
0.4
0.3
0.2
0.1
0.0
0.55
0.60
0.65
0.70
0.75
Standard deviation
RPLP0
log2 expression
1
0
−1
−2
Raw_Control
GeNorm_Control
LEMming_Control
Raw_WY14
GeNorm_WY14
LEMming_WY14
Variance−mean plot
Raw
GeNorm
LEMming
mean
0.00
−0.05
−0.10
−0.15
0.5
0.6
0.7
0.8
0.9
1.0
1.1
Standard deviation
