# Supplementary material for: LEMming: A Linear Error Model to Normalize Parallel Quantitative Real-Time PCR (qPCR) Data as an Alternative to Reference Gene Based Methods
Source: PLoS One. 2015 Sep 1;10(9):e0135852. doi: 10.1371/journal.pone.0135852 (PMC4556681; doi:10.1371/journal.pone.0135852)
Supplement: S2 File — (PDF) [file pone.0135852.s002.pdf]

Supplement 3 - Derivation of LEMming error model with data set 2

1 Derivation of LEMming error model

LEMming uses multivariable regression in order to exclude systematic errors like probe pipetting errors, systematic batch effects and sample errors from parallel qPCR measurements. An assay for parallel qPCR measurements is shown in Fig. 1. Here we demonstrate that these errors are identified as systematic errors by using an additional ssDNA measurement in data set 2.

1.1 Measurements of single-stranded DNA (ssDNA) quantity

The content of single stranded DNA (ssDNA) in each sample was determined using the Quant-iT OliGreen ssDNA Assay Kit (O11492, Life Technologies), according to the manufacturer's instruction in 96-well plate using microtiterplate reader Enspire (Perkin Elmer). Briefly, working solution of the Quant-iT^{TM} OliGreen® reagent for the low-range standard curve (spanning from 100 pg/mL to 50 ng/mL) was prepared immediately before the experiment using M13 primer. For sample quantification, equal volumes (15 µL) of a cDNA sample and working solution was mixed and incubated in the darkness for 5 minutes. Finally, samples were excited at 485 nm and fluorescence intensity was measured at 520 nm.

1.2 Identification of batch effects $\tilde{\epsilon}$ by ssDNA

The ssDNA measurement facilitates the identification of batch effects $\tilde{\epsilon}$ in the experimental setting. We recommend to perform an analysis of variance (ANOVA) of all variables in the experiment trying to explain differences in the average expression of all genes in a sample. In data set 2 (DS2) the variables *cDNA* ($p < 2 \times 10^{-16}$) and *Ansatz* ($p < 0.0164$) have a significant effect on the average expression. If these variables also show a significant impact on the ssDNA measurement, the effect is confirmed by a second independent measurement technique. In DS2 only the variable *cDNA* ($p < 2 \times 10^{-16}$) has a significant impact on ssDNA. On an average the ssDNA signal in the second cDNA is 318.95 ng ml^{-1} higher than in the first cDNA batch. In this experiment the material of the sample was pipetted twice in order to get two cDNA batches. Afterwards we found out, that the sample material was pipetted with the same pipette in one procedure. The effect that a lower amount of sample material is pipetted in first pipette event than in the second is able to explain the systematic error. We argue, that this effect can be excluded as a systematic error.

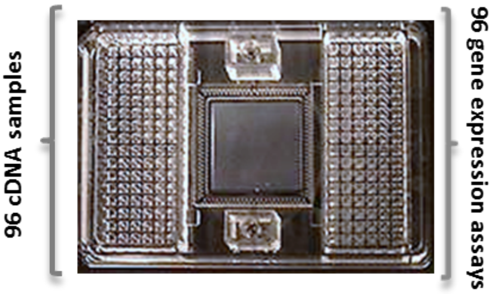

Figure 1: Schematic representation of a 96×96 dynamic array for Fluidigm Biomark^{TM}measurements.

1.3 Sample error ϵ_S

Parallel qPCR techniques evaluate a selection of genes and they might show a different average expression under treatment conditions than the average of all genes measured for example with microarrays. Thus with parallel qPCR, the treatment conditions can have effects on the average over all genes in a sample. At least the average over all genes in samples from the same experimental conditions should show a high correlation with the ssDNA measurement.
The linear model

$$\bar{C}_t \sim ssDNA : Treatment \quad (1)$$

predicts the average over all genes in a sample $\bar{C}_t$ from a ssDNA factor per condition. The statistic correlation for DS2 data is shown in Fig. 2. The coefficients of the model are all significant with $p < 10^{-11}$. The adjusted R^2 is 0.3562. The residuals have a standard deviation of 0.76 C_t. If the *Actb* measurement replaces the ssDNA measurement in the model Eq. 1, the variance of the residuals is 0.32 C_t.
Fig. 2 shows, that the mean C_t value of a sample is predictable by its ssDNA content. However, the high variation of the residuals demonstrates, that using measurements of ssDNA content for normalization would perform worse compared to RGs in this case.

The relation between ssDNA content and mean C_t per sample shows, that this effect needs to be corrected by a proper normalization technique. The fact that the technical replicates are close together in Fig. 2 indicates, that there is a potential to further correct the sample wise error. The experimental setting of DS2 allows to examine the pipetting error per sample. Each cDNA is measured four times (see Fig. 3). It is pipetted twice in a well and each well is measured two times by an equal primer set. Thus, the pipetting error of the sample is assigned to the residuals of the following model:

$$\bar{C}_t \sim Sample : Primerset \quad (2)$$

The contrasts *sample per primer* cover 60% of the standard error of $\bar{C}_t$. These contrasts include the experimental condition effect and effects resulting from different cDNA content. The remaining difference is the pipetting error of a cDNA into the well. The residuals of this model cover 40% of the standard error of $\bar{C}_t$. This demonstrates that the pipetting error is a relevant source of variation and

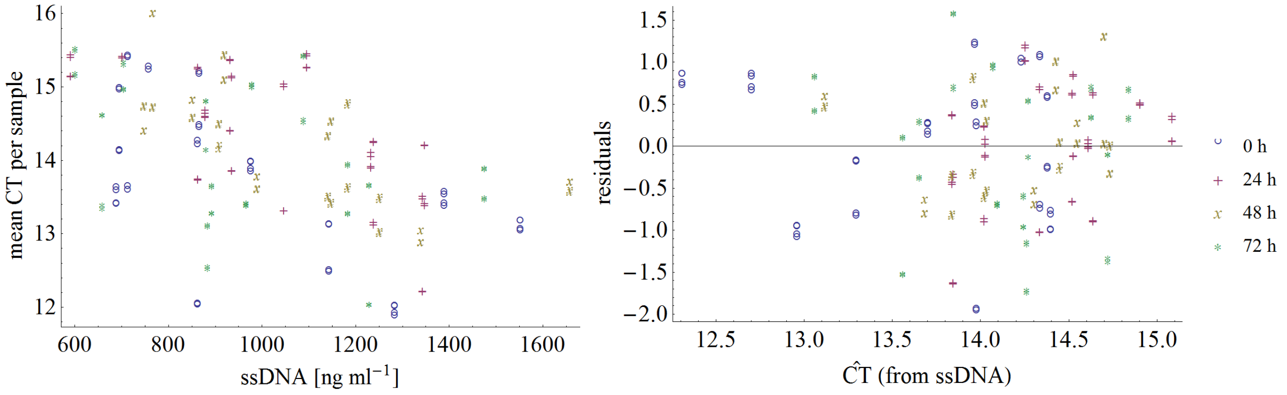

Figure 2: The different symbols represent the 4 different experimental conditions. Left: mean C_t per sample over ssDNA content. The relation between ssDNA and mean sample C_t is described by the model Eq. 1. Right shows the residuals of the linear model over predicted values. Four symbols that are close together on a vertical line are technical replicates with the same ssDNA content.

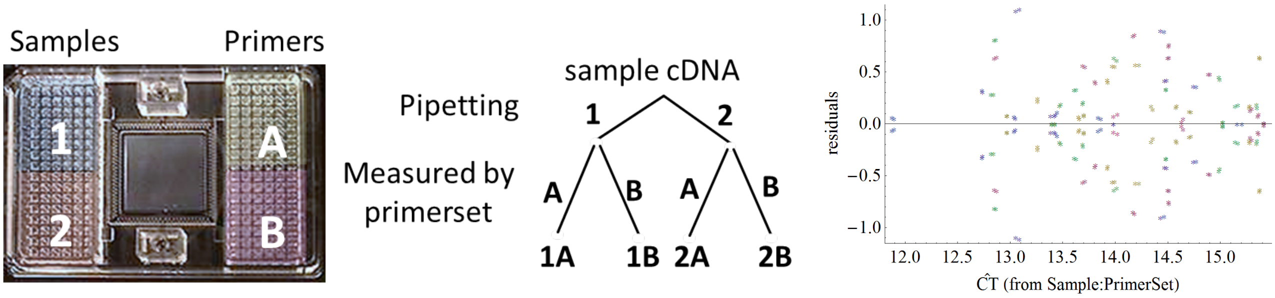

Figure 3: Left and middle: Scheme for experimental setting of data set 2. Each sample cDNA is measured 4 times. Right: The model Eq. 2 introduces a contrast for each sample cDNA per primer set. The resulting residuals represent the differences between **1A** and **2A** as well as between **1B** and **2B**, which is the cDNA sample pipetting error.

should be regarded by a proper normalization technique.

Here we identified two relevant sources of errors (cDNA amount and sample pipetting), that effect the average expression of a sample. If RGs with constant expression under all treatment conditions are available, they can be used for normalization and will be able to correct for these errors. Alternatively, LEMming introduces the sample error ϵ_S which also corrects for different cDNA amounts and sample pipetting errors. Measurements without ϵ_S are equalized in the average expression level over all genes. However, due to the reason that treatment conditions can have effects on the average over all genes in a sample, this global treatment effect Δ_T needs to be removed before estimating the ϵ_S. Otherwise Δ_T would be removed with ϵ_S. Because of this, the LEMming method is independent from RGs.

1.3.1 Global treatment effect Δ_T or systematic sample error $\tilde{\epsilon}$

We already discussed, that the mean C_t-value of a selection of genes might change with treatment conditions. Fig. 4 left shows, that this is the case for DS2. The mean C_t at 24 h is significantly higher than at 0 h (t-test: p$\leq 10^{-4}$), which means that the mean mRNA concentration of selected genes is lower. Using the LEMming approach, we need to decide, whether the difference between mean C_t values is a global treatment effect Δ_T or a systematic sample error $\tilde{\epsilon}$. The fluorescence signal of ssDNA is increased at 24 h , 48 h and 72 h compared to 0 h (see Fig. 4 right) indicating that there is systematic mRNA-concentration error between the time conditions. However, the three reference genes selected by *geNorm* (see Fig. 7) show small variations (one-sided t-test Eef1a1 0h vs 72h: p= 0.025; Hprt 0h vs 72h: p= 0.024; Ppia 0h vs 48h: p= 0.053; see Fig. 4), but non of them clearly reflects the course of the median of the ssDNA quantification.
The aim of reference genes and the *geNorm* normalization factor is to correct for those sample effects. The correlation between ssDNA and *geNorm* factor is indeed positive (Spearman Rank correlation 0.563), but is rather low (see Fig. 5). There are large differences of *geNorm* normalization factors of samples with nearly the same amount of ssDNA and vice versa.
If the stability of reference genes is questionable, the ssDNA is a possibility to correct for a systematic mRNA-concentration error. In order to use the ssDNA measurement, the relationship between ssDNA fluorescence signal and mRNA concentration needs to be determined. This is currently not available for this experiment. Thus, we cannot distinguish between a global treatment effect and a systematic sample error.
In fact ssDNA measurements indicate higher cDNA concentrations at later time points, while the mean C_t suggests a lower mRNA concentration of the selected gene set (see Fig. 4). Thus, we would expect that the global treatment effect is underestimated. Hence, we decided to introduce a one-

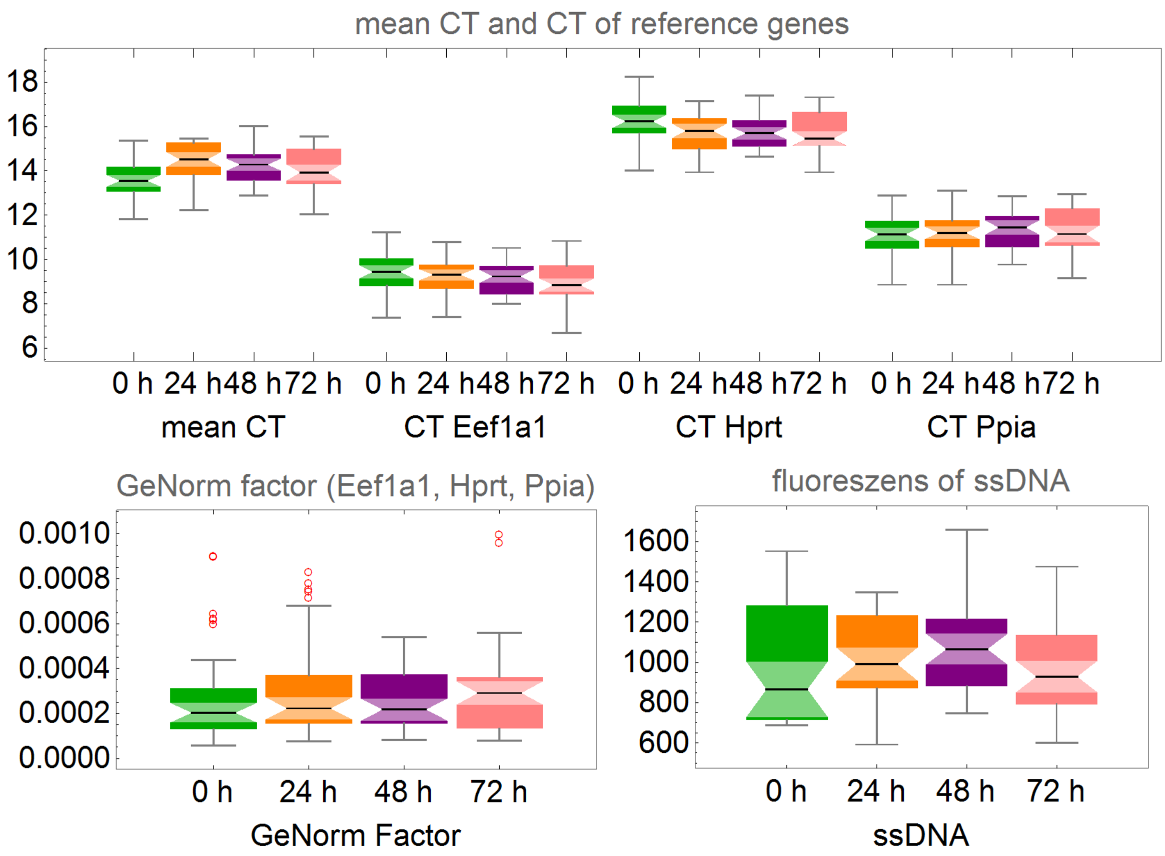

Figure 4: Above: Comparison between mean C_t value of samples and C_t values of the three most stable reference genes. Left: *geNorm* normalization factor (NF, see section 2) of samples computed with the reference genes (*Eef1a1*, *Hprt* and *Ppia*). Right: Content of single stranded DNA (ssDNA) in ng/mL of samples (determined using the Quant-iT OliGreen ssDNA Assay Kit).

sided threshold $\Delta_{th,j}$. If under condition j the $-\Delta C_t > 0$ (which means that the mRNA amount is increased), the differential expression is only confident if the threshold $\Delta_{th,j}$ is exceeded. We defined that threshold as the 75% quantile of potential reference genes under the condition j:

$$\Delta_{th,j} := q^j_{0.75}((-\Delta C_t)_i) \quad (\forall i \text{ of potential reference gene index}) \tag{3}$$

$$-\Delta C_t < 0 : p < \alpha \Rightarrow \text{ confident lower expression under condition } j \tag{4}$$

$$-\Delta C_t > \Delta_{th,j} : p < \alpha \Rightarrow \text{ confident higher expression under condition } j \tag{5}$$

Supplement 4 contains data tables of data set 2. The color coding in worksheet *LEM_Diff.xlsx* reflects the classification into confident and not confident differential expression.

1.4 Probe pipetting error per array $\epsilon_{P:A}$

Pipetting of the probe and the channel for probe transportation on the assay are sources of systematic errors that should be regarded if assays are compared or probes are pipetted multiply on an assay. The idea is, that the average expression of a gene measured with the same probe well should be the same as with other probe wells.

Thus, it is important, that the experimental design ensures a proper distribution of samples on different assays. If for example all untreated samples are on array one and all treated samples are on array two, it is impossible to assign the difference between expression of the gene either to the treatment per gene

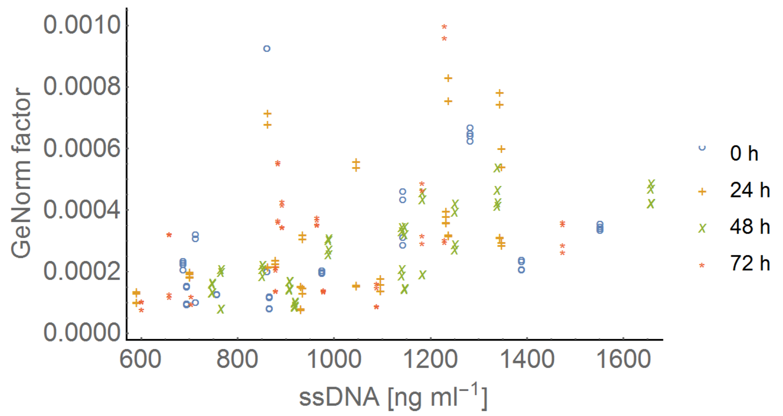

Figure 5: Content of single stranded DNA (ssDNA) versus the *geNorm* factor per sample.

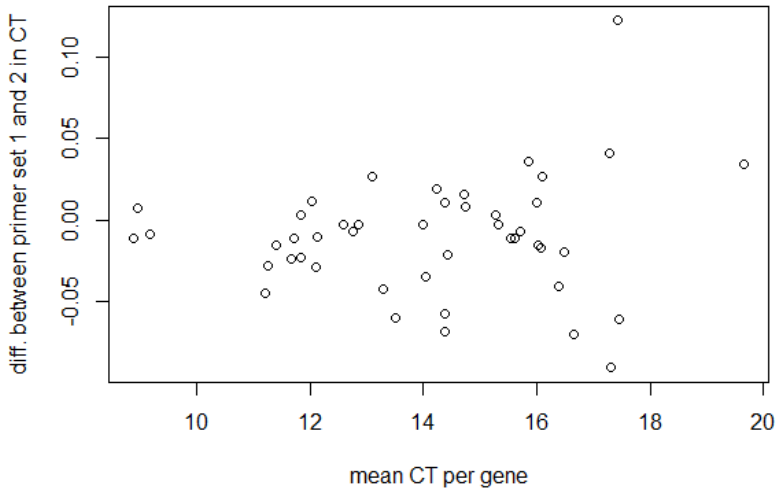

Figure 6: Probe pipetting error $\epsilon_{P:A}$ in data set 2: Mean C_t of genes over the difference between primer set one and two per gene.

or to the array per gene effect.
For example, the latin square design is very common and will fulfill the requirement of proper experimental design.

In case of data set 2 each probe is pipetted twice on the array. Thus, the difference between primer set one and two per gene corresponds to the probe pipetting error ($\epsilon_{P:A}$). Fig. 6 shows the $\epsilon_{P:A}$. The standard deviation of the difference between the primer sets is 0.0356 C_t. The variation increases with increasing cycling time.

1.5 Conclusion for LEMming

The above described sources for errors are regarded in the LEMming method. To prevent the exclusion of effects that are part of the treatment effects, it is important to regard the correct order in the linear

error model. E.g systematic errors, treatment effects and sample errors are effects that would be included in the sample error. It is the aim to maintain the treatment effect without the systematic errors but to reduce the variance by the remaining sample error. For this reason it is essential to stick to the following order of estimation.

1. The probe pipetting error $\epsilon_{P:A}$ is excluded, which also accounts for interassay variation. The experimental design need to ensure that a proper distribution of samples on different assays is guaranteed.
2. Estimation of systematic errors $\tilde{\epsilon}$ in residuals.
3. Estimate the global treatment effect Δ_T.
4. Estimate sample error ϵ_S.

The LEMming corrected data are the residuals of the sample error plus the global treatment effect.

LEMming Example for data set 2:

```
library(xlsx)
# import data
rawdata <- read.xlsx("S4_DS2_Exp_MET.xlsx","RawData_Processed")
# make an expression set
es = new("ExpressionSet", exprs=t(apply(rawdata[,-c(1:8)],2,as.numeric)))
pData(es) =  rawdata[,c(1:8)] # add the annotation frame
...
# LEMming for data set 2
# 1. probe pitting error (each primer is pipetted twice)
design <- model.matrix(~factor(es$Primer)-1)
fit <- lmFit(exprs(es), design)
summary(fit)
# build new expression set with residuals
es.Resid1 = new("ExpressionSet", exprs= residuals(fit,exprs(es)))
pData(es.Resid1)=pData(es)

# compute mean expression of each sample
pData(es.Resid1) = cbind(pData(es), "ExprMean"=colMeans(exprs(es.Resid1)))

# 2. Sytematic errors (in this case cDNA amount)
lm.sysErr <-lm(ExprMean ~ cDNA -1, data=pData(es.Resid1))
summary(lm.sysErr)
pData(es.Resid1)["ResidSysErr"]<- residuals(lm.sysErr)
pData(es.Resid1)["PredSysErr"]<- predict(lm.sysErr)

# 3. Treatment effect
lm.treat <-lm(ResidSysErr ~ Time -1, data=pData(es.Resid1))
summary(lm.treat)
pData(es.Resid1)["PredTreatEff"]<- predict(lm.treat)
pData(es.Resid1)["ResidTreatEff"]<- residuals(lm.treat)

# 4. Sample error
lm.SE <-lm(ResidTreatEff ~ Sample:cDNA:Replicate -1, data=pData(es.Resid1))
summary(lm.SE)
pData(es.Resid1)["PredSE"]<- predict(lm.SE)
pData(es.Resid1)["ResidSE"]<- residuals(lm.SE)

# make LEMming corrected expression set
## subtract systematic error and sample error
pData(es.Resid1)["Corr"]<- pData(es.Resid1)["PredSysErr"] + pData(es.Resid1)["PredSE"]
corVals<-as.data.frame(as.matrix(replicate(dim(exprs(es.Resid1))[1],pData(es.Resid1)["Corr"][,1]))
## mean per gen for correct average expression values
genMean<-as.data.frame(as.matrix(replicate(dim(exprs(es.Resid1))[2],rowMeans(exprs(es)))))
```

```
## corrected Values:
normVals<-as.matrix(exprs(es.Resid1)-t(corVals)+genMean)
es.Norm = new("ExpressionSet", exprs= normVals)
pData(es.Norm)=pData(es.Resid1)

# calculate differential expression as you want
f <- pData(es.Norm)$Factor
design <- model.matrix(~0+f)
colnames(design) = levels(f)
fit <- lmFit(es.Norm , design)
# all factors together
contrast.matrix <- makeContrasts("h0-h24","h0-h48","h0-h72", levels=design)
fit2 <- contrasts.fit(fit , contrast.matrix)
fit3 <- eBayes(fit2 , robust=TRUE) # robust version
topTableF(fit3 , adjust="bonferroni", lfc=0, number=dim(exprs(es))[1], p.value=1)
```

2 *geNorm*

geNorm is a method for the identification of the most stable reference genes given a list of potential candidate reference genes [1]. The method relies on the principle that the expression ratio between two ideal reference genes remains constant over all samples. Variation in this ratio however corresponds to a decreased expression stability. It should be noted that the expression of all reference genes is required to be not co-regulated.
Given an experiment with m samples and n candidate reference genes the pairwise variation v for each pair of genes i and j (with $i, j \in n$) is calculated. $v_{i,j}$ is defined as the standard deviation (sd) of the log2 transformed expression ratios $\frac{x_{k,i}}{x_{k,j}}$ for all samples k (with $k \in m$) (equation 6). For each gene i the corresponding pairwise variation values $v_{i,j}$ are then summarized to form the control gene-stability measure M_i using the arithmetic mean over all samples k (equation 7).

$$v_{i,j} = sd(log_2\{\frac{x_{i,k}}{x_{j,k}}\}_{k=1\rightarrow m}) \tag{6}$$

$$M_i = \frac{\sum_{j=1}^{n} v_{i,j}}{n-1} \tag{7}$$

Starting with the full set of candidate reference genes n the gene with the largest stability measure M is stepwise excluded until the two constitutively expressed housekeeping genes with the most stable expression remain. This provides a ranking of the candidate reference genes for their use as part of the normalization factor (NF). NF itself is defined as the geometric mean of the expression values x of the most stable genes l (with $l \in n$) in sample k (NF_l). Starting with a minimal number of two of the most stable reference genes for normalization ($NF_{l=2}$, with $l = 2 \dots n$) the pairwise variation v between the two sequential normalization factors NF_l and NF_{l+1} ($v_{NF_l,NF_{l+1}}$) is calculated over all samples k (see equation 6). In general, larger values of pairwise variation v between two sequential normalization factors suggest that the additional reference gene should be included in the final normalization factor. The average expression stability M for housekeeping genes of data set2 is shown in Fig. 7.
Based on the normalization factor the ΔC_t normalized expression value of gene i in sample k is defined as:

$$x'_{i,k} = \log_2\left(\frac{2^{-x_{i,k}}}{NF_k}\right). \tag{8}$$

This corresponds to the values provided in Supplement 2, 4 and 7 of the corresponding data sets DS1, DS2 and DS3. For comparsion to the raw data and the LEMming processed data we normalized these

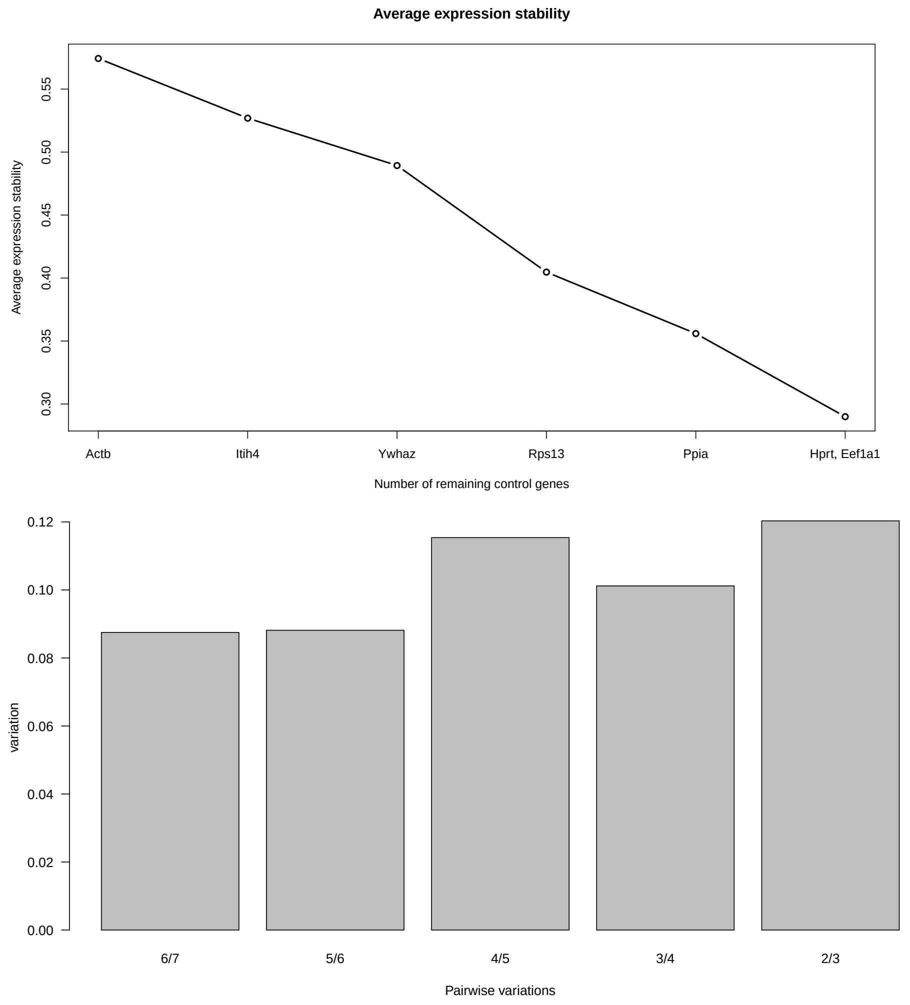

Figure 7: Average expression stability and pairwise variation according to *geNorm*.

ΔC_t values against the respective mean ΔC_t value of the control group ($x'_{i,k} - mean(x'_{i,control})$, with *control* denoting for all samples of the control group). Thus, $\Delta\Delta C_t$ values corresponding to log2 fold-changes were calculated to compare the results of the different methods.

References

[1] Vandesompele J, De Preter K, Pattyn F, Poppe B, Van Roy N, et al. (2002) Accurate normalization of real-time quantitative RT-PCR data by geometric averaging of multiple internal control genes. Genome Biology 3: research0034.1–research0034.11.

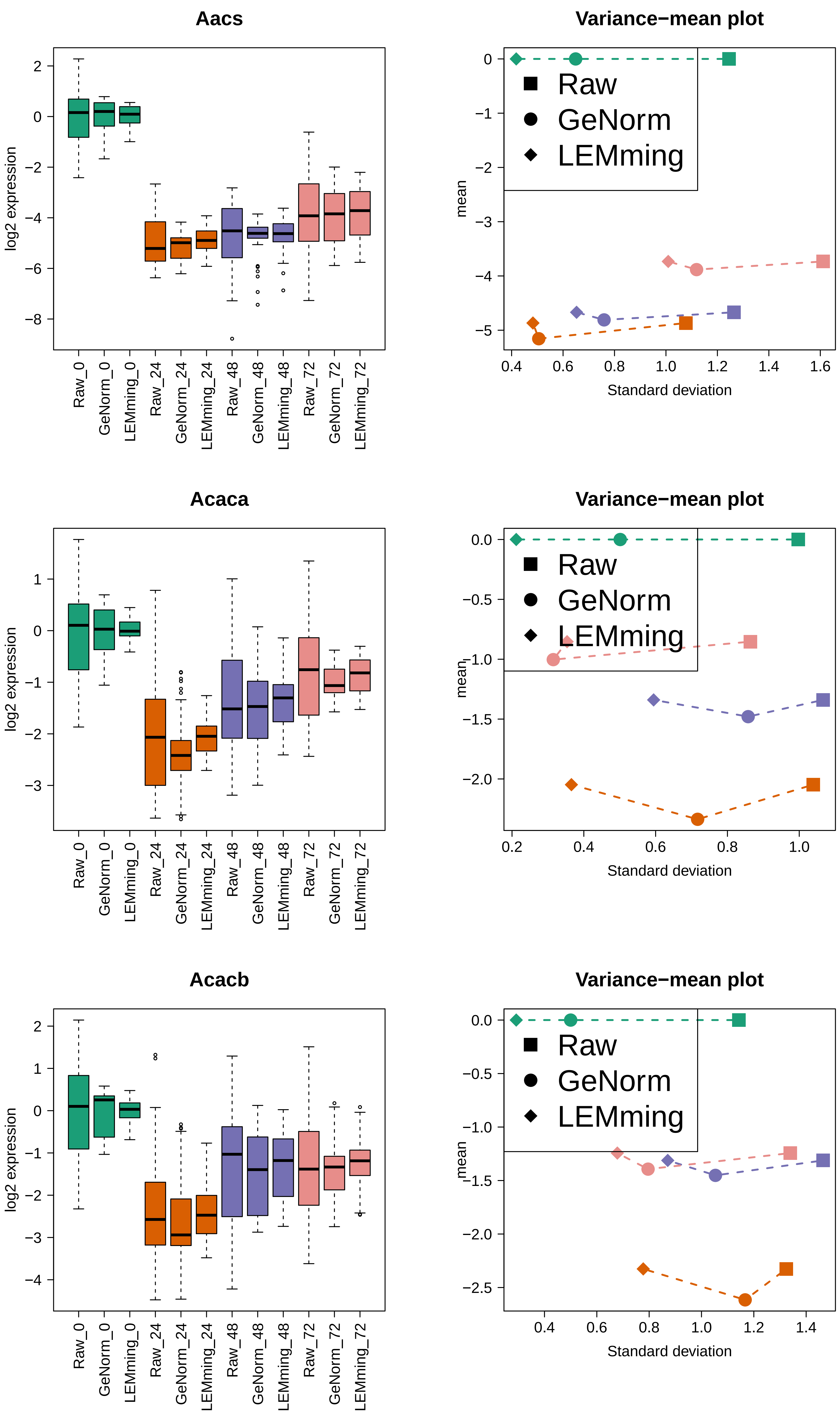
Aacs
log2 expression
2
0
−2
−4
−6
−8
Raw_0
GeNorm_0
LEMming_0
Raw_24
GeNorm_24
LEMming_24
Raw_48
GeNorm_48
LEMming_48
Raw_72
GeNorm_72
LEMming_72
Variance−mean plot
Raw
GeNorm
LEMming
mean
0
−1
−2
−3
−4
−5
0.4
0.6
0.8
1.0
1.2
1.4
1.6
Standard deviation
Acaca
log2 expression
1
0
−1
−2
−3
Raw_0
GeNorm_0
LEMming_0
Raw_24
GeNorm_24
LEMming_24
Raw_48
GeNorm_48
LEMming_48
Raw_72
GeNorm_72
LEMming_72
Variance−mean plot
Raw
GeNorm
LEMming
mean
0.0
−0.5
−1.0
−1.5
−2.0
0.2
0.4
0.6
0.8
1.0
Standard deviation
Acacb
log2 expression
2
1
0
−1
−2
−3
−4
Raw_0
GeNorm_0
LEMming_0
Raw_24
GeNorm_24
LEMming_24
Raw_48
GeNorm_48
LEMming_48
Raw_72
GeNorm_72
LEMming_72
Variance−mean plot
Raw
GeNorm
LEMming
mean
0.0
−0.5
−1.0
−1.5
−2.0
−2.5
0.4
0.6
0.8
1.0
1.2
1.4
Standard deviation

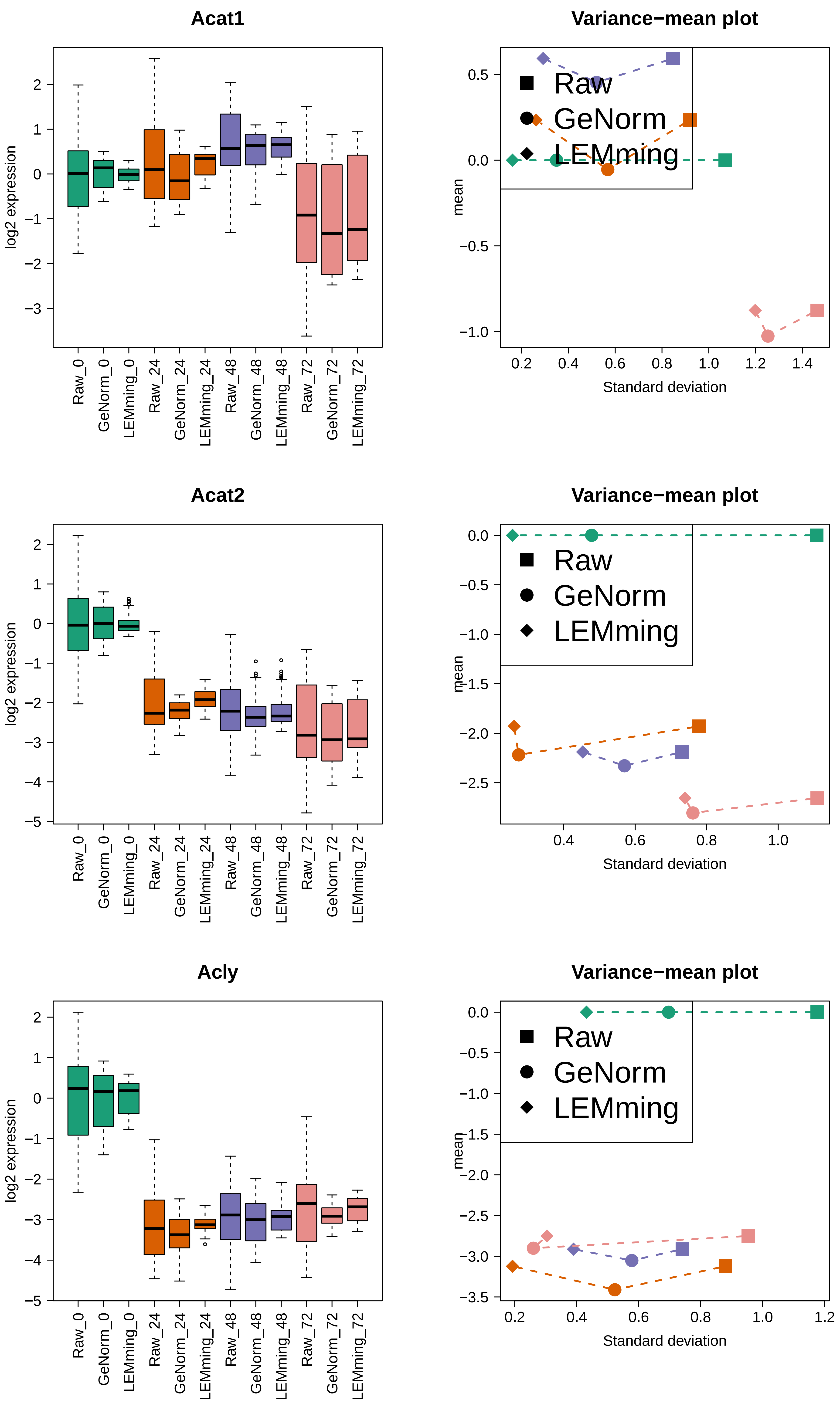
Acat1
Variance−mean plot
Acat2
Variance−mean plot
Acly
Variance−mean plot
log2 expression
mean
Standard deviation
Raw
GeNorm
LEMming
Raw_0
GeNorm_0
LEMming_0
Raw_24
GeNorm_24
LEMming_24
Raw_48
GeNorm_48
LEMming_48
Raw_72
GeNorm_72
LEMming_72

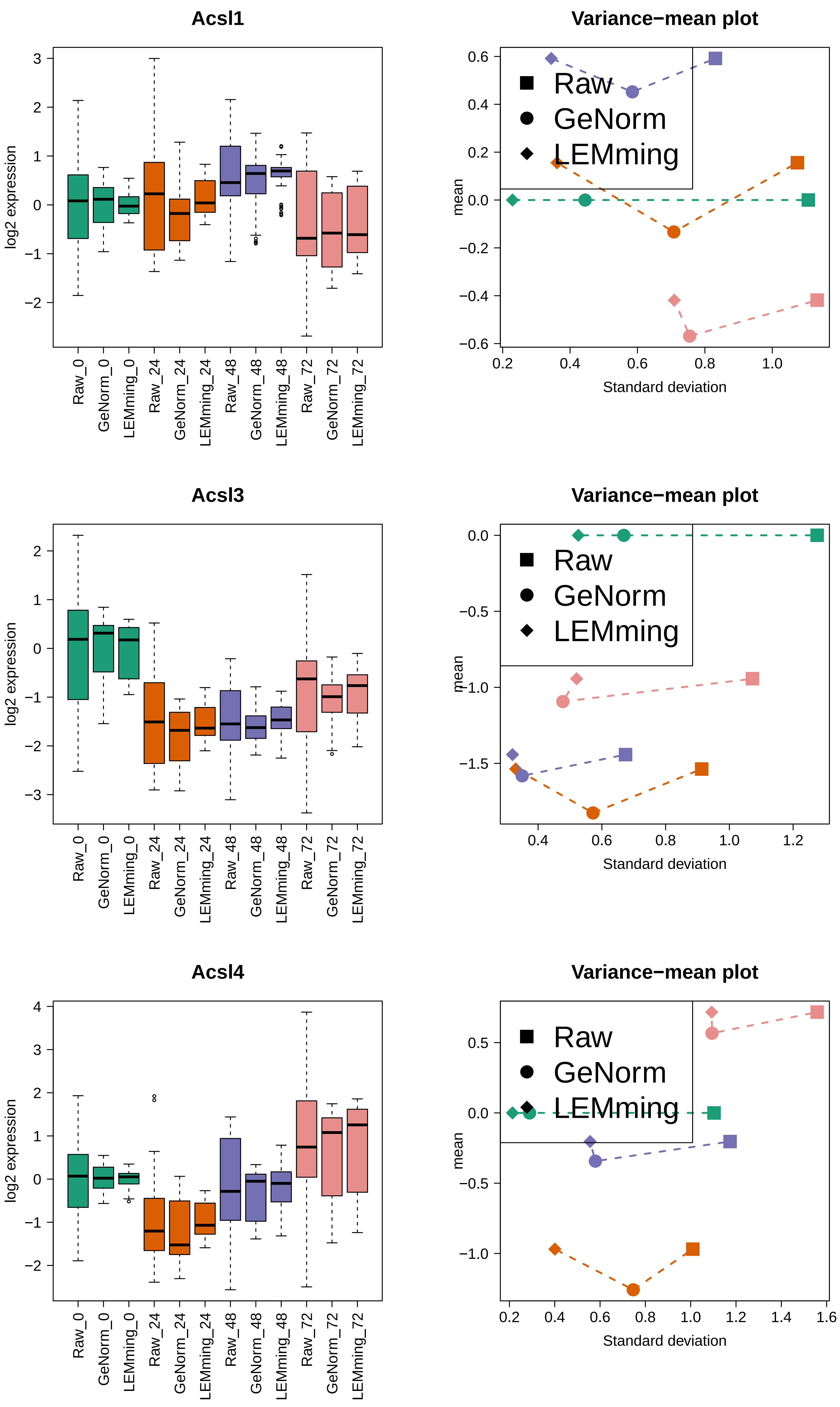
Acsl1
Variance−mean plot
Acsl3
Variance−mean plot
Acsl4
Variance−mean plot
log2 expression
mean
Standard deviation
Raw
GeNorm
LEMming
Raw_0
GeNorm_0
LEMming_0
Raw_24
GeNorm_24
LEMming_24
Raw_48
GeNorm_48
LEMming_48
Raw_72
GeNorm_72
LEMming_72

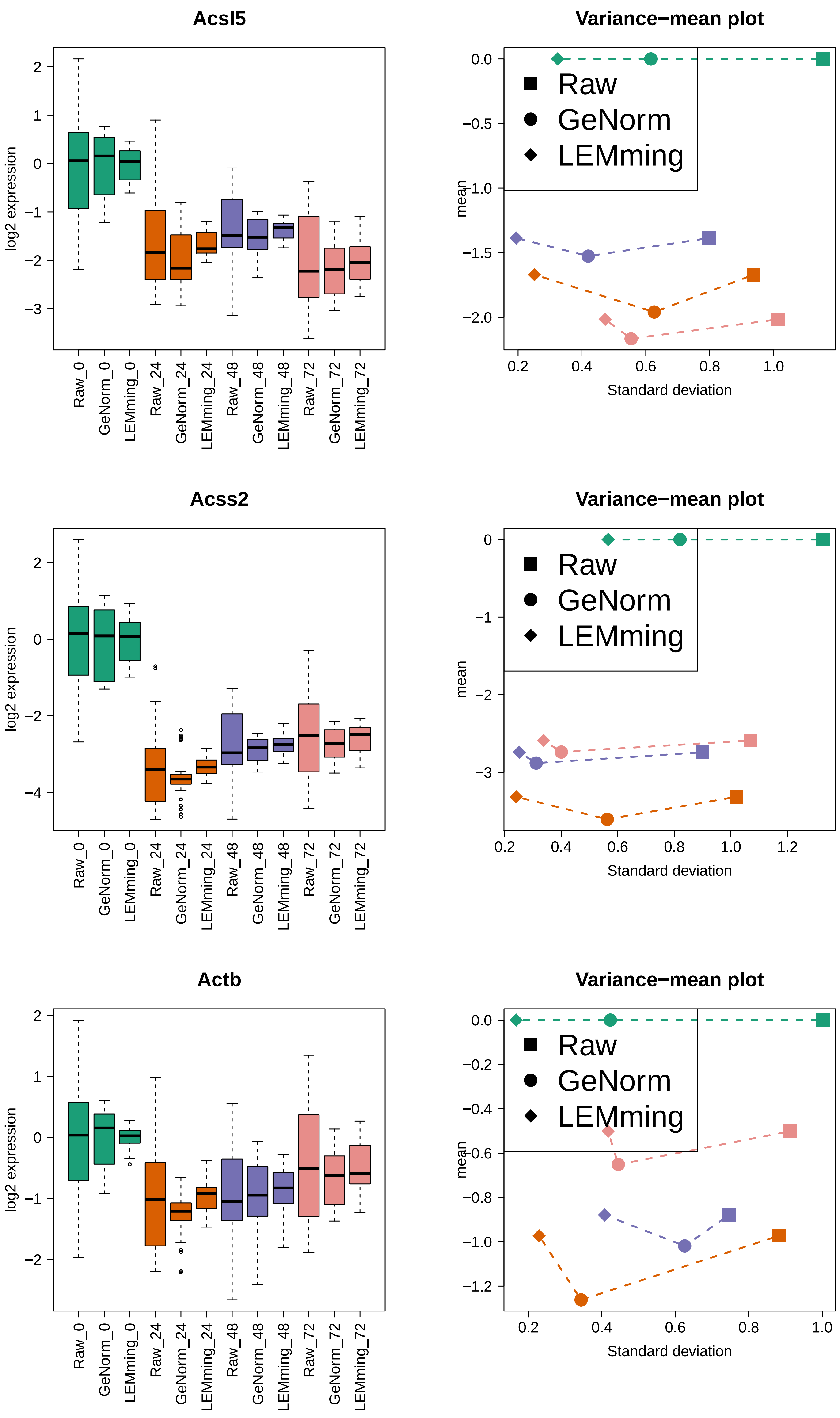

Acsl5
Variance−mean plot
Acss2
Variance−mean plot
Actb
Variance−mean plot
log2 expression
mean
Standard deviation
Raw
GeNorm
LEMming
Raw_0
GeNorm_0
LEMming_0
Raw_24
GeNorm_24
LEMming_24
Raw_48
GeNorm_48
LEMming_48
Raw_72
GeNorm_72
LEMming_72

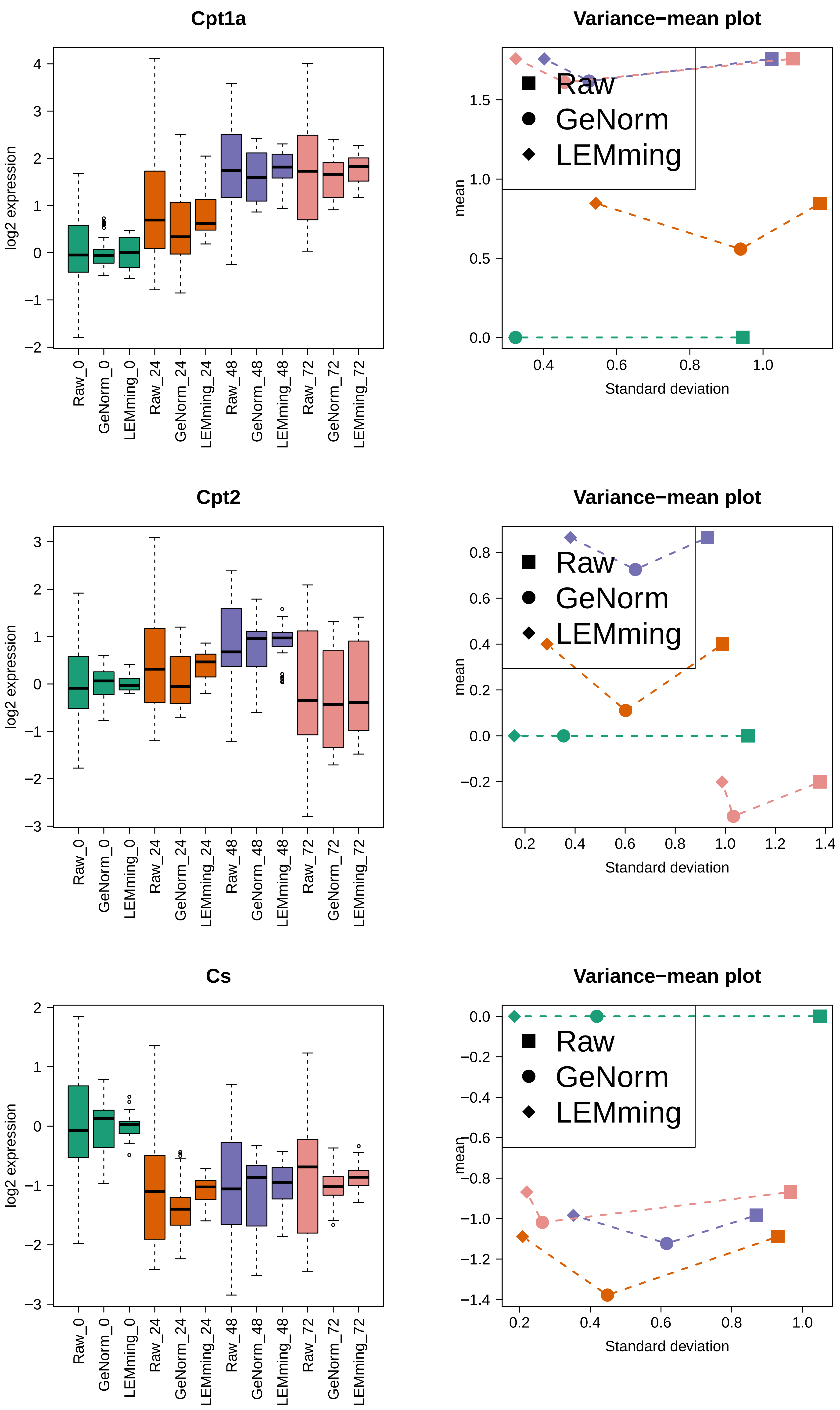

Cpt1a
log2 expression
Raw_0
GeNorm_0
LEMming_0
Raw_24
GeNorm_24
LEMming_24
Raw_48
GeNorm_48
LEMming_48
Raw_72
GeNorm_72
LEMming_72
Variance−mean plot
mean
Standard deviation
Raw
GeNorm
LEMming
Cpt2
log2 expression
Raw_0
GeNorm_0
LEMming_0
Raw_24
GeNorm_24
LEMming_24
Raw_48
GeNorm_48
LEMming_48
Raw_72
GeNorm_72
LEMming_72
Variance−mean plot
mean
Standard deviation
Raw
GeNorm
LEMming
Cs
log2 expression
Raw_0
GeNorm_0
LEMming_0
Raw_24
GeNorm_24
LEMming_24
Raw_48
GeNorm_48
LEMming_48
Raw_72
GeNorm_72
LEMming_72
Variance−mean plot
mean
Standard deviation
Raw
GeNorm
LEMming

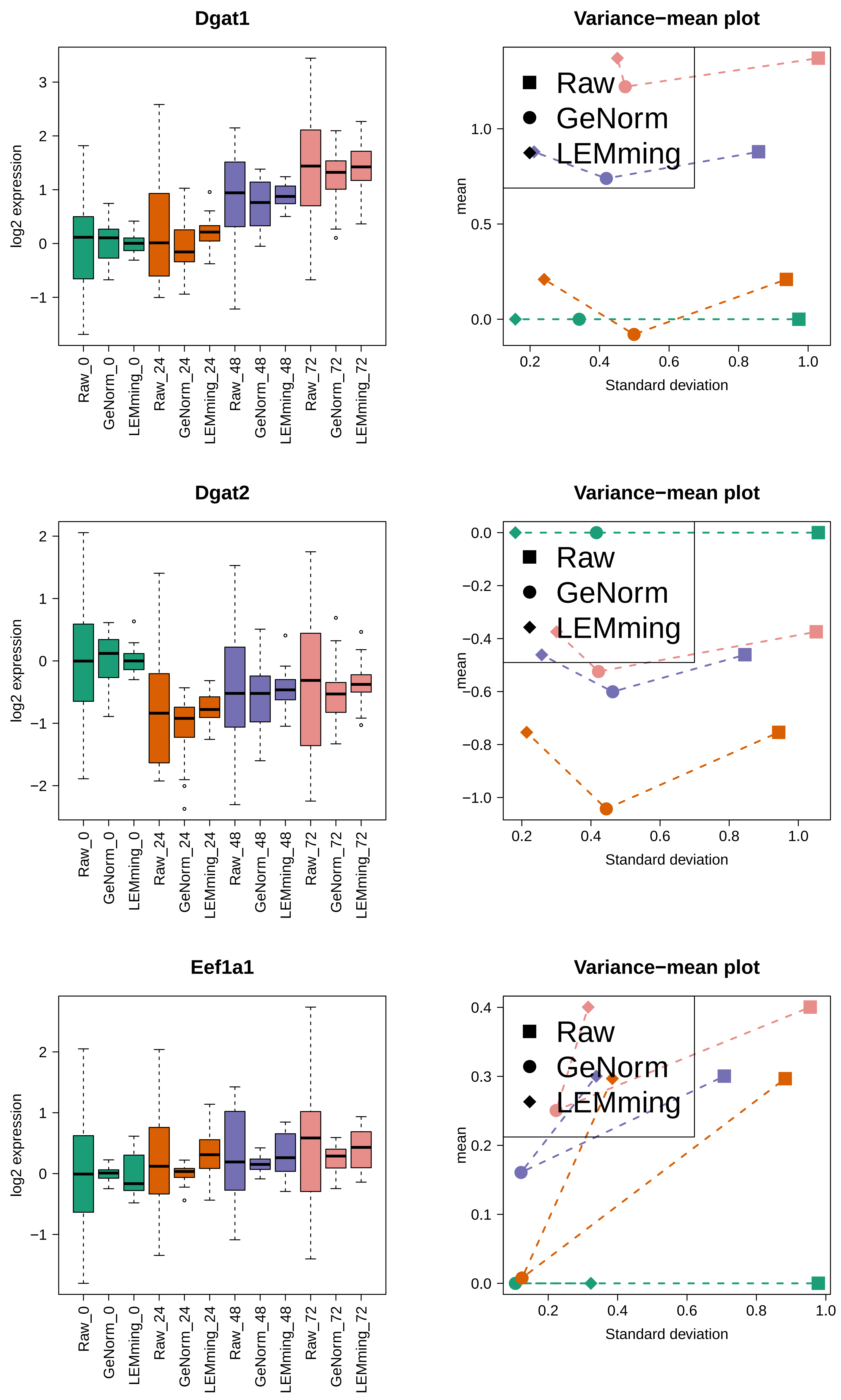
Dgat1
Variance−mean plot
Dgat2
Variance−mean plot
Eef1a1
Variance−mean plot
log2 expression
mean
Standard deviation
Raw
GeNorm
LEMming
Raw_0
GeNorm_0
LEMming_0
Raw_24
GeNorm_24
LEMming_24
Raw_48
GeNorm_48
LEMming_48
Raw_72
GeNorm_72
LEMming_72

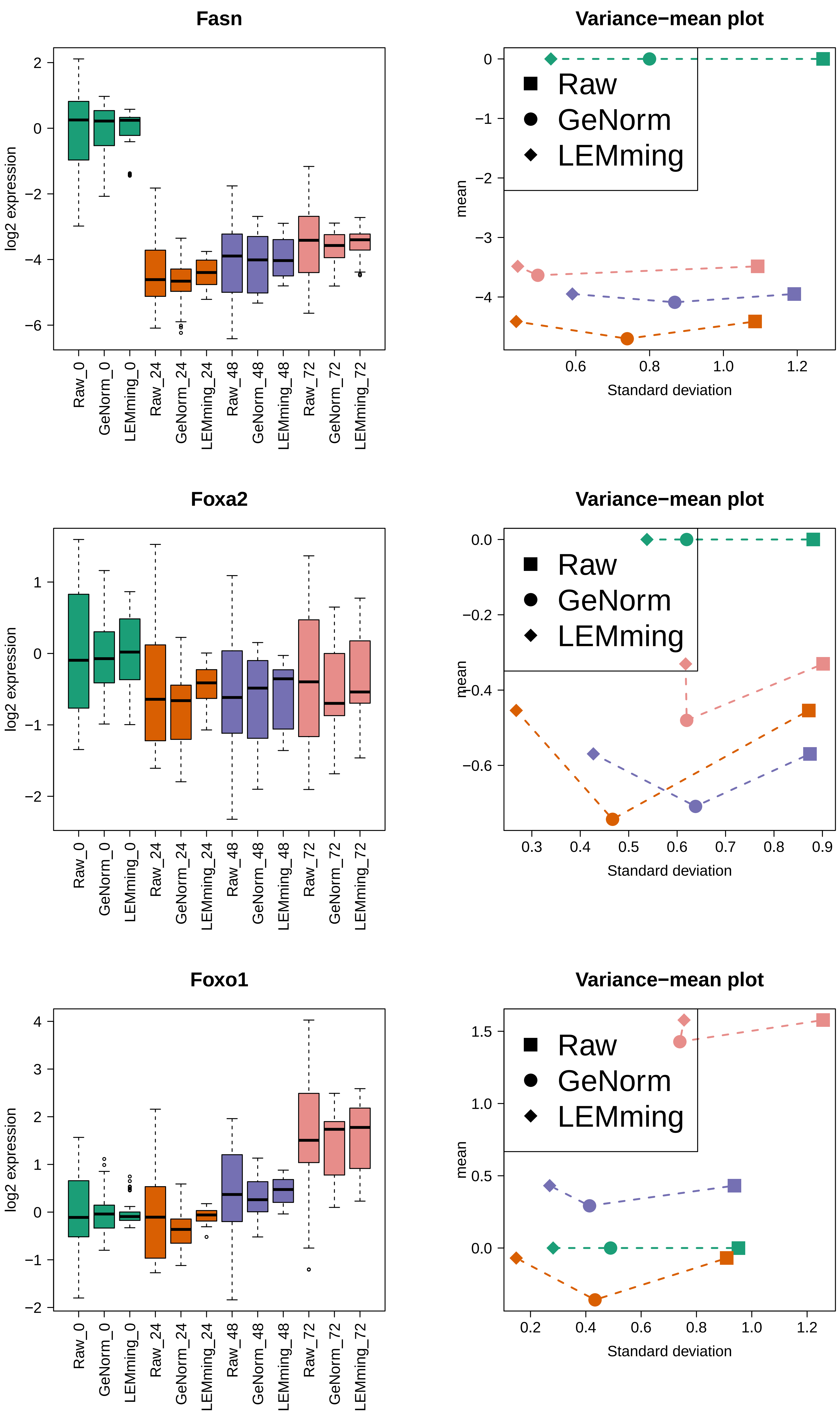

Fasn
log2 expression
2
0
−2
−4
−6
Raw_0
GeNorm_0
LEMming_0
Raw_24
GeNorm_24
LEMming_24
Raw_48
GeNorm_48
LEMming_48
Raw_72
GeNorm_72
LEMming_72
Variance−mean plot
mean
0
−1
−2
−3
−4
Raw
GeNorm
LEMming
0.6
0.8
1.0
1.2
Standard deviation
Foxa2
log2 expression
1
0
−1
−2
Raw_0
GeNorm_0
LEMming_0
Raw_24
GeNorm_24
LEMming_24
Raw_48
GeNorm_48
LEMming_48
Raw_72
GeNorm_72
LEMming_72
Variance−mean plot
mean
0.0
−0.2
−0.4
−0.6
Raw
GeNorm
LEMming
0.3
0.4
0.5
0.6
0.7
0.8
0.9
Standard deviation
Foxo1
log2 expression
4
3
2
1
0
−1
−2
Raw_0
GeNorm_0
LEMming_0
Raw_24
GeNorm_24
LEMming_24
Raw_48
GeNorm_48
LEMming_48
Raw_72
GeNorm_72
LEMming_72
Variance−mean plot
mean
1.5
1.0
0.5
0.0
Raw
GeNorm
LEMming
0.2
0.4
0.6
0.8
1.0
1.2
Standard deviation

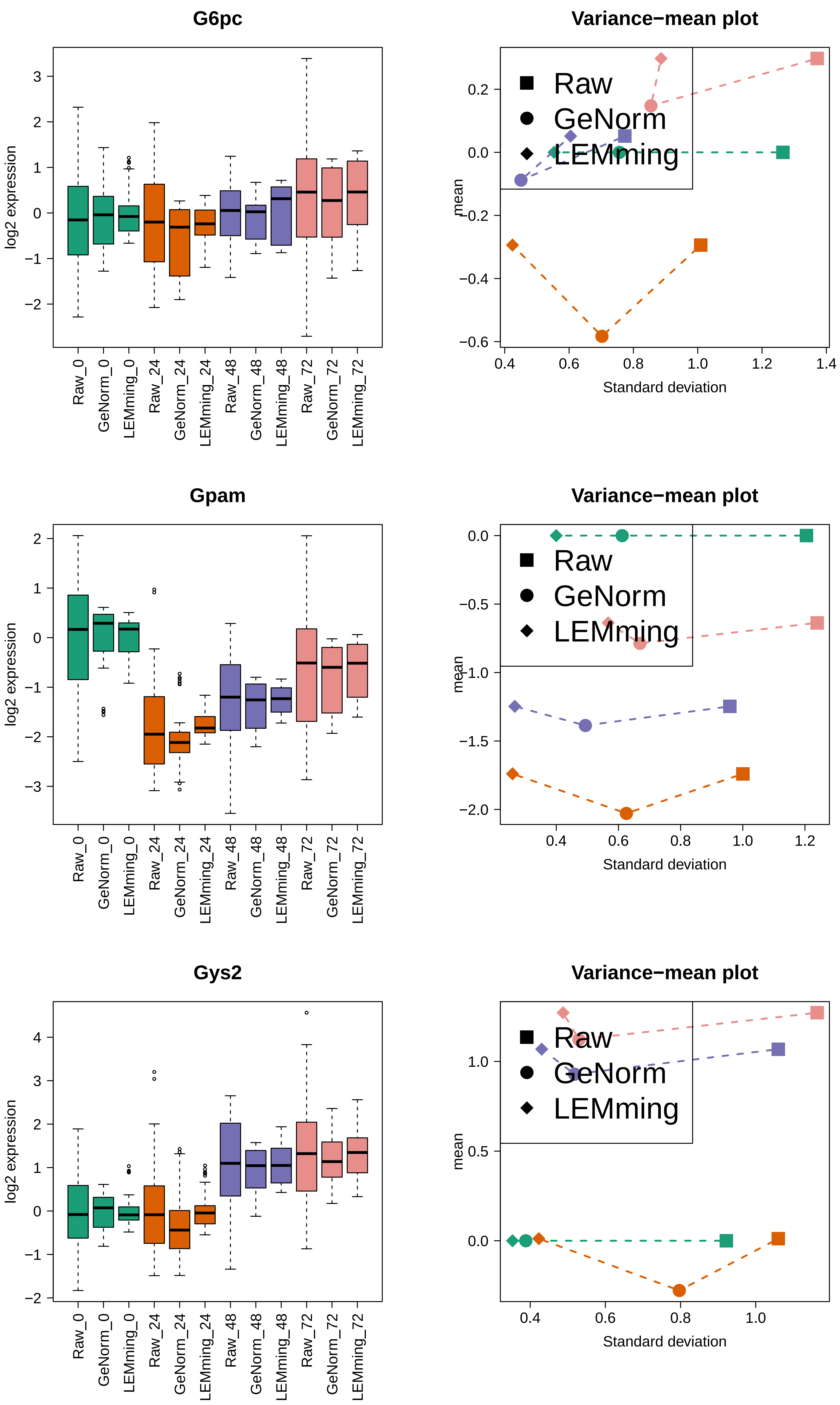

G6pc
Variance−mean plot
Gpam
Variance−mean plot
Gys2
Variance−mean plot
log2 expression
mean
Standard deviation
Raw
GeNorm
LEMming
Raw_0
GeNorm_0
LEMming_0
Raw_24
GeNorm_24
LEMming_24
Raw_48
GeNorm_48
LEMming_48
Raw_72
GeNorm_72
LEMming_72

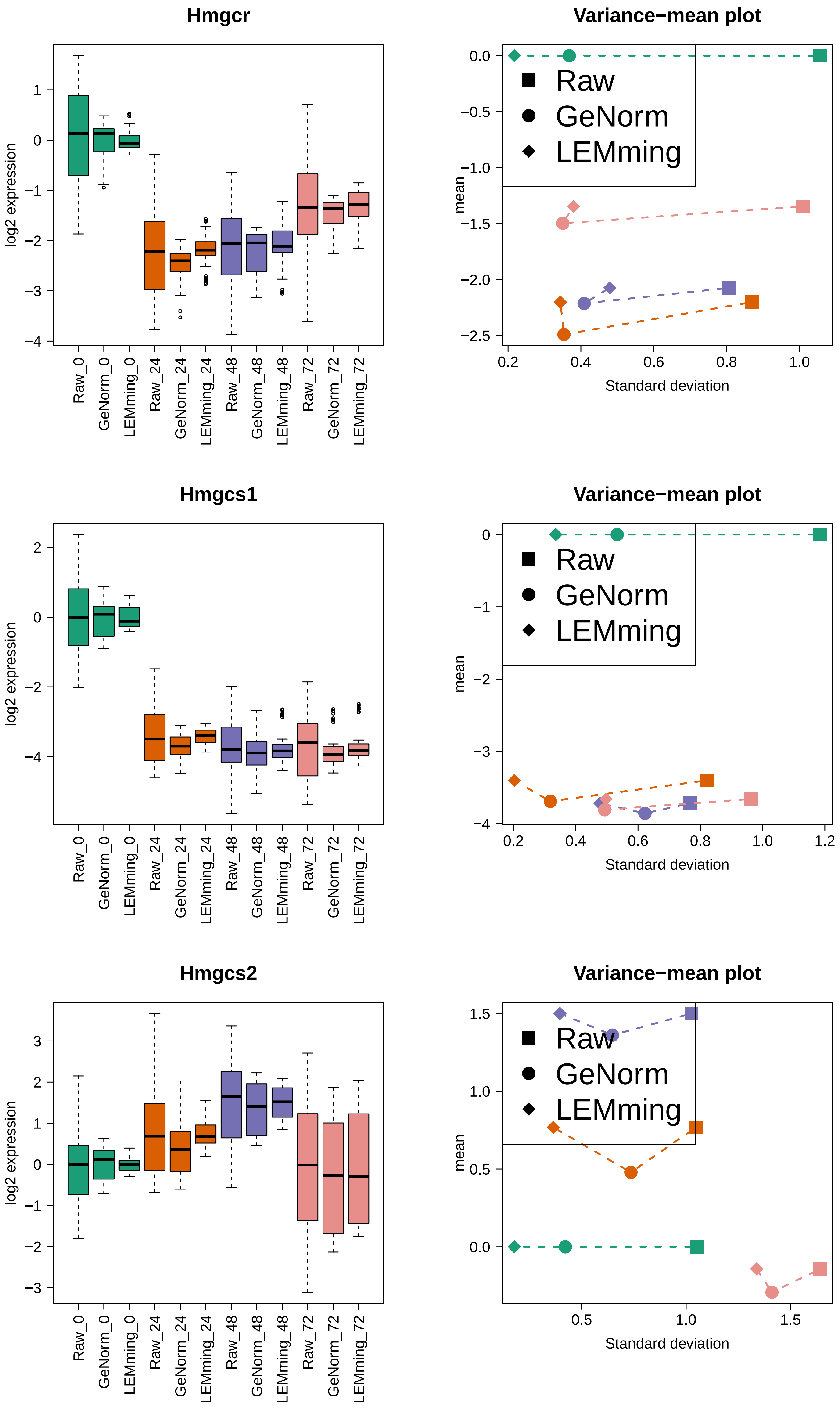
Hmgcr
log2 expression
Raw_0
GeNorm_0
LEMming_0
Raw_24
GeNorm_24
LEMming_24
Raw_48
GeNorm_48
LEMming_48
Raw_72
GeNorm_72
LEMming_72
Variance−mean plot
Raw
GeNorm
LEMming
mean
Standard deviation
Hmgcs1
Hmgcs2

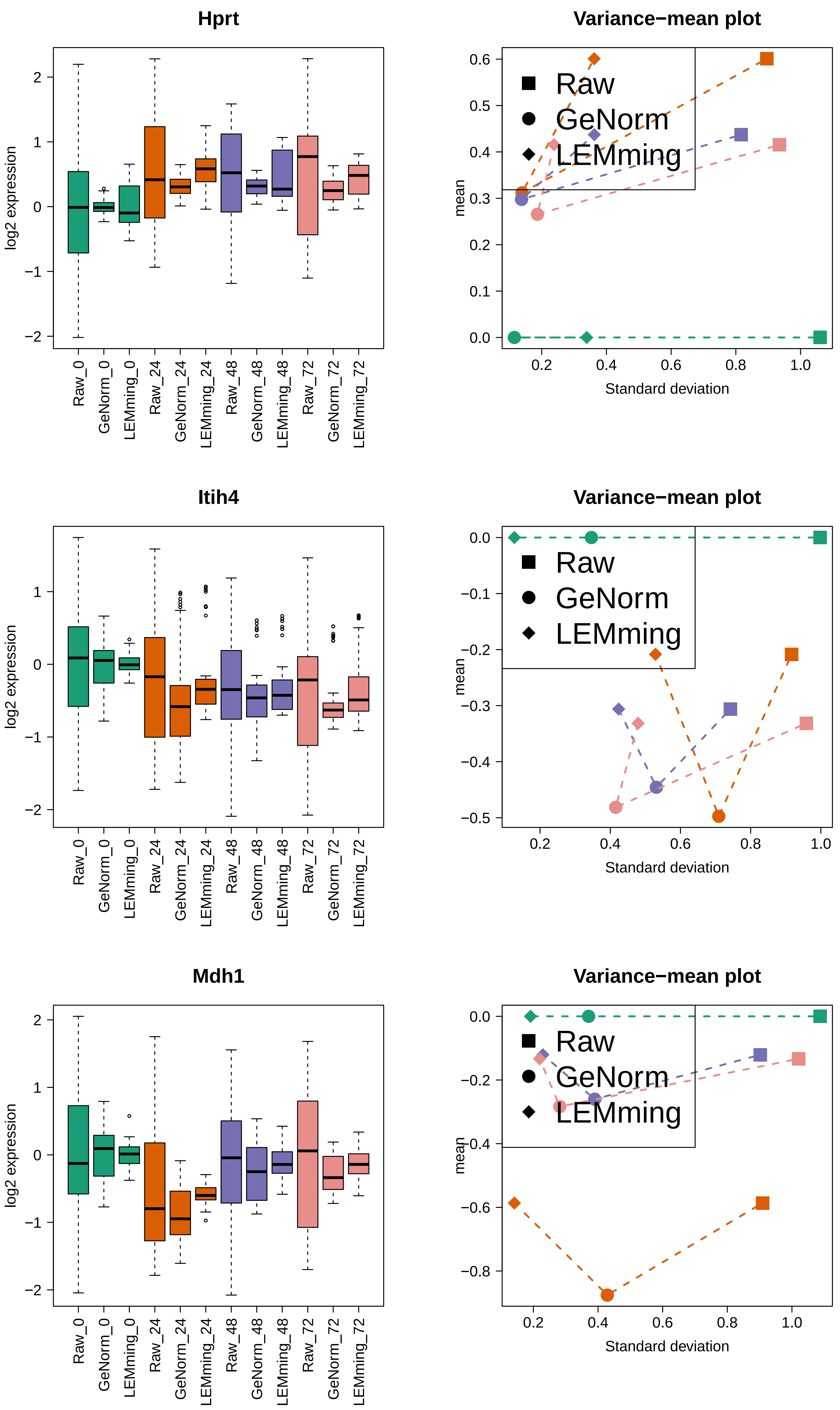
Hprt
log2 expression
Raw_0
GeNorm_0
LEMming_0
Raw_24
GeNorm_24
LEMming_24
Raw_48
GeNorm_48
LEMming_48
Raw_72
GeNorm_72
LEMming_72
Variance−mean plot
Raw
GeNorm
LEMming
mean
Standard deviation
Itih4
log2 expression
Variance−mean plot
Raw
GeNorm
LEMming
mean
Standard deviation
Mdh1
log2 expression
Variance−mean plot
Raw
GeNorm
LEMming
mean
Standard deviation

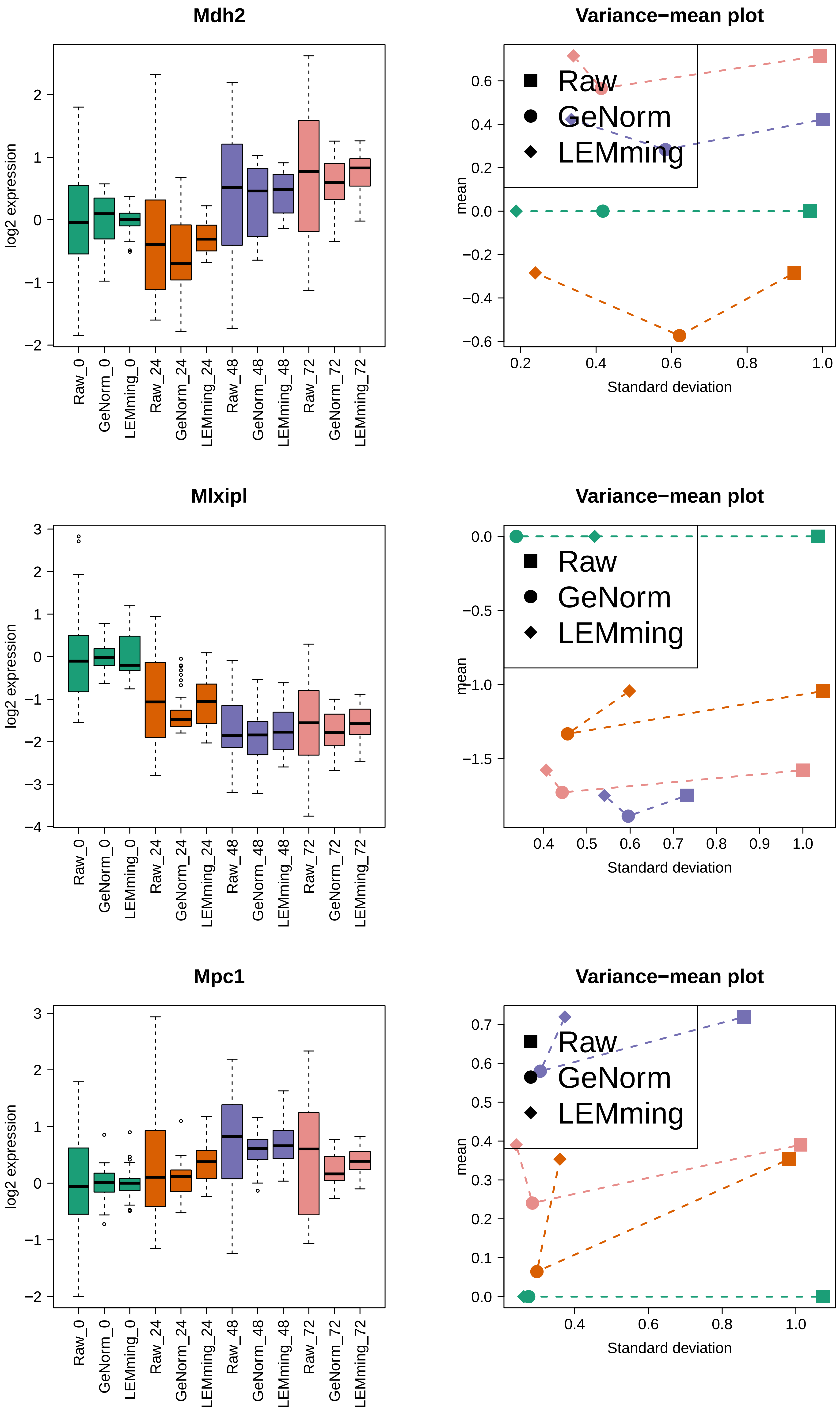
Mdh2
log2 expression
2
1
0
−1
−2
Raw_0
GeNorm_0
LEMming_0
Raw_24
GeNorm_24
LEMming_24
Raw_48
GeNorm_48
LEMming_48
Raw_72
GeNorm_72
LEMming_72
Variance−mean plot
Raw
GeNorm
LEMming
mean
0.6
0.4
0.2
0.0
−0.2
−0.4
−0.6
0.2
0.4
0.6
0.8
1.0
Standard deviation
Mlxipl
log2 expression
3
2
1
0
−1
−2
−3
−4
Raw_0
GeNorm_0
LEMming_0
Raw_24
GeNorm_24
LEMming_24
Raw_48
GeNorm_48
LEMming_48
Raw_72
GeNorm_72
LEMming_72
Variance−mean plot
Raw
GeNorm
LEMming
mean
0.0
−0.5
−1.0
−1.5
0.4
0.5
0.6
0.7
0.8
0.9
1.0
Standard deviation
Mpc1
log2 expression
3
2
1
0
−1
−2
Raw_0
GeNorm_0
LEMming_0
Raw_24
GeNorm_24
LEMming_24
Raw_48
GeNorm_48
LEMming_48
Raw_72
GeNorm_72
LEMming_72
Variance−mean plot
Raw
GeNorm
LEMming
mean
0.7
0.6
0.5
0.4
0.3
0.2
0.1
0.0
0.4
0.6
0.8
1.0
Standard deviation

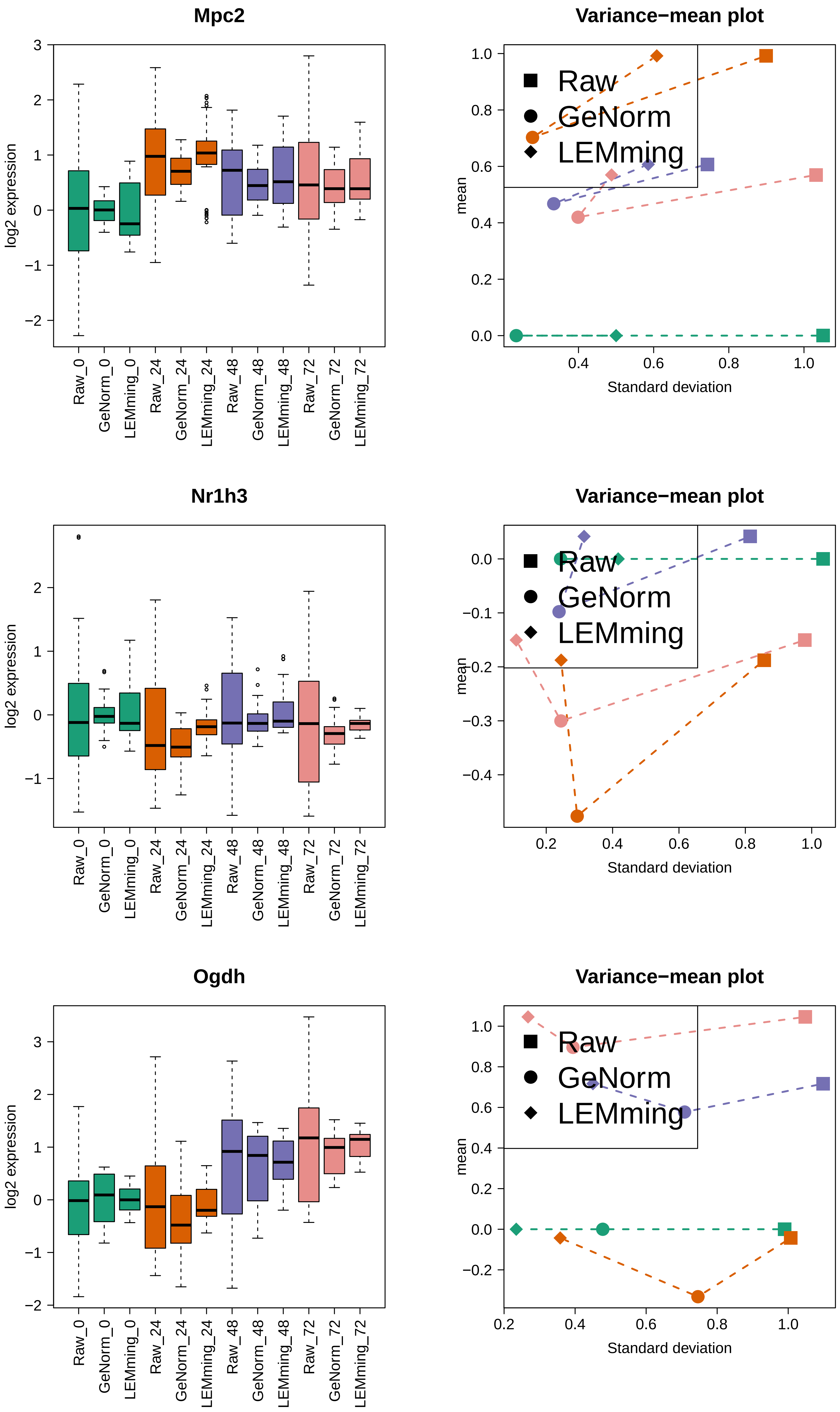

Mpc2
log2 expression
Raw_0
GeNorm_0
LEMming_0
Raw_24
GeNorm_24
LEMming_24
Raw_48
GeNorm_48
LEMming_48
Raw_72
GeNorm_72
LEMming_72
Variance−mean plot
mean
Standard deviation
Raw
GeNorm
LEMming
Nr1h3
log2 expression
Variance−mean plot
mean
Standard deviation
Raw
GeNorm
LEMming
Ogdh
log2 expression
Variance−mean plot
mean
Standard deviation
Raw
GeNorm
LEMming

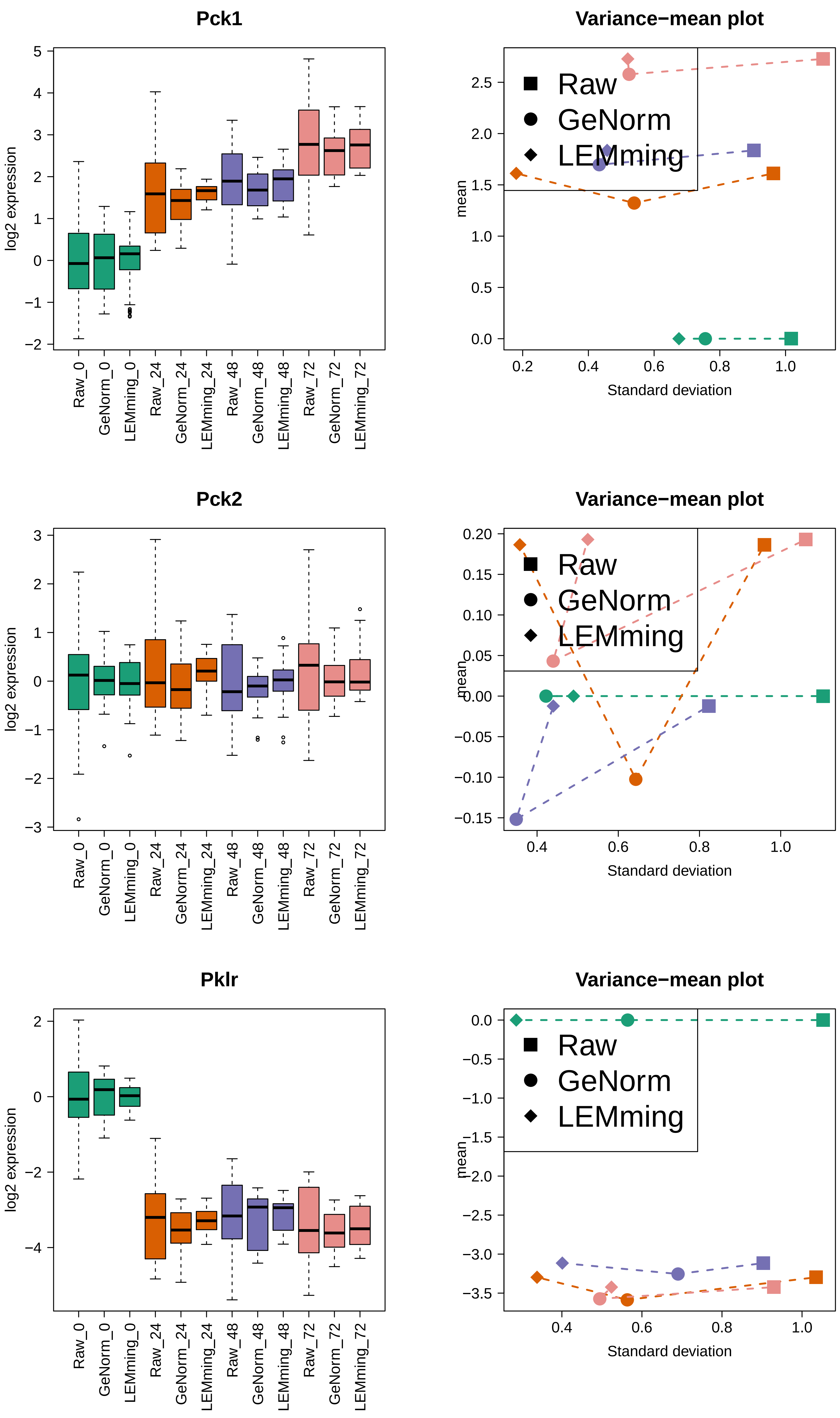
Pck1
log2 expression
Raw_0
GeNorm_0
LEMming_0
Raw_24
GeNorm_24
LEMming_24
Raw_48
GeNorm_48
LEMming_48
Raw_72
GeNorm_72
LEMming_72
Variance−mean plot
Raw
GeNorm
LEMming
mean
Standard deviation
Pck2
Pklr

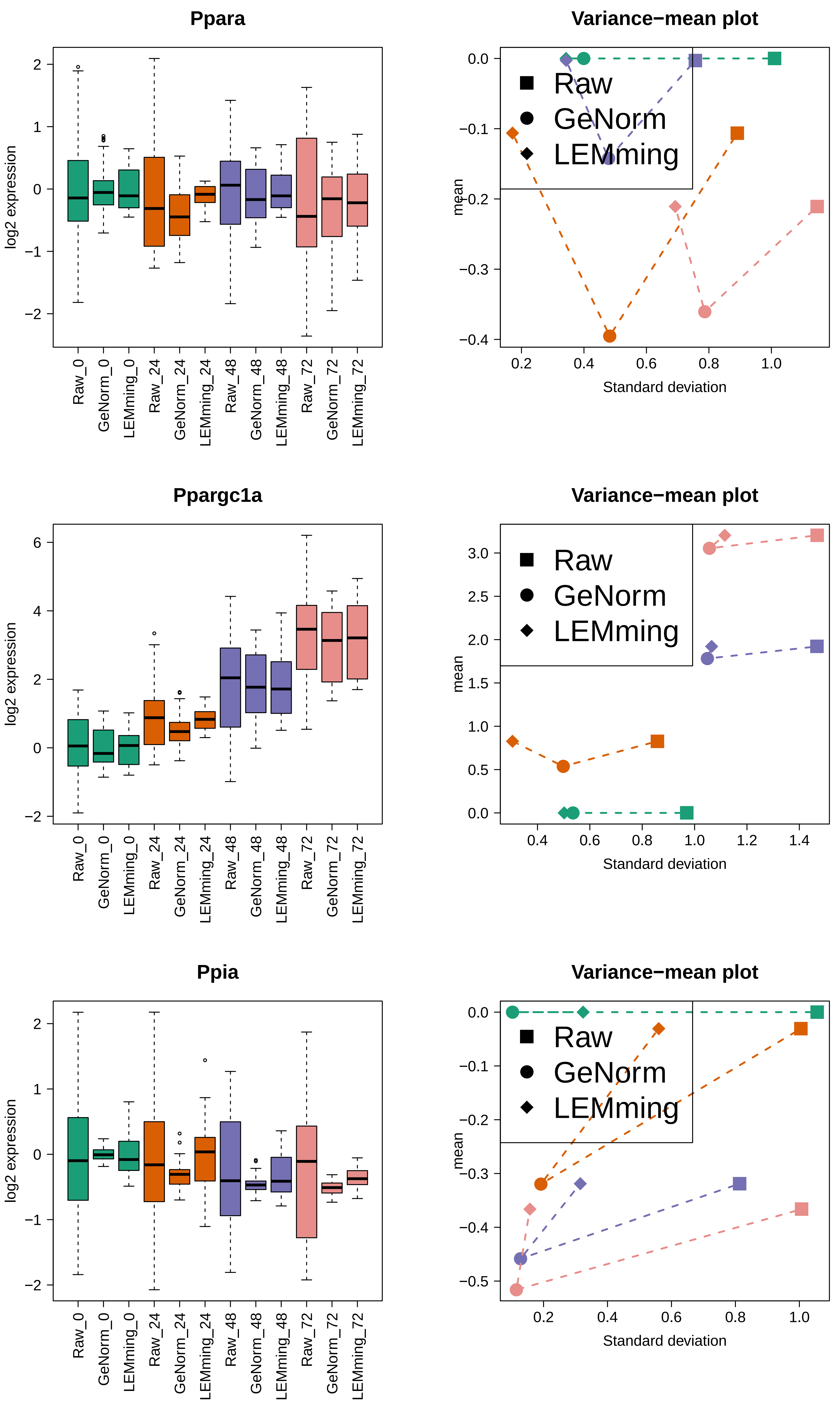
Ppara
Variance−mean plot
Ppargc1a
Variance−mean plot
Ppia
Variance−mean plot
log2 expression
mean
Standard deviation
Raw
GeNorm
LEMming
Raw_0
GeNorm_0
LEMming_0
Raw_24
GeNorm_24
LEMming_24
Raw_48
GeNorm_48
LEMming_48
Raw_72
GeNorm_72
LEMming_72

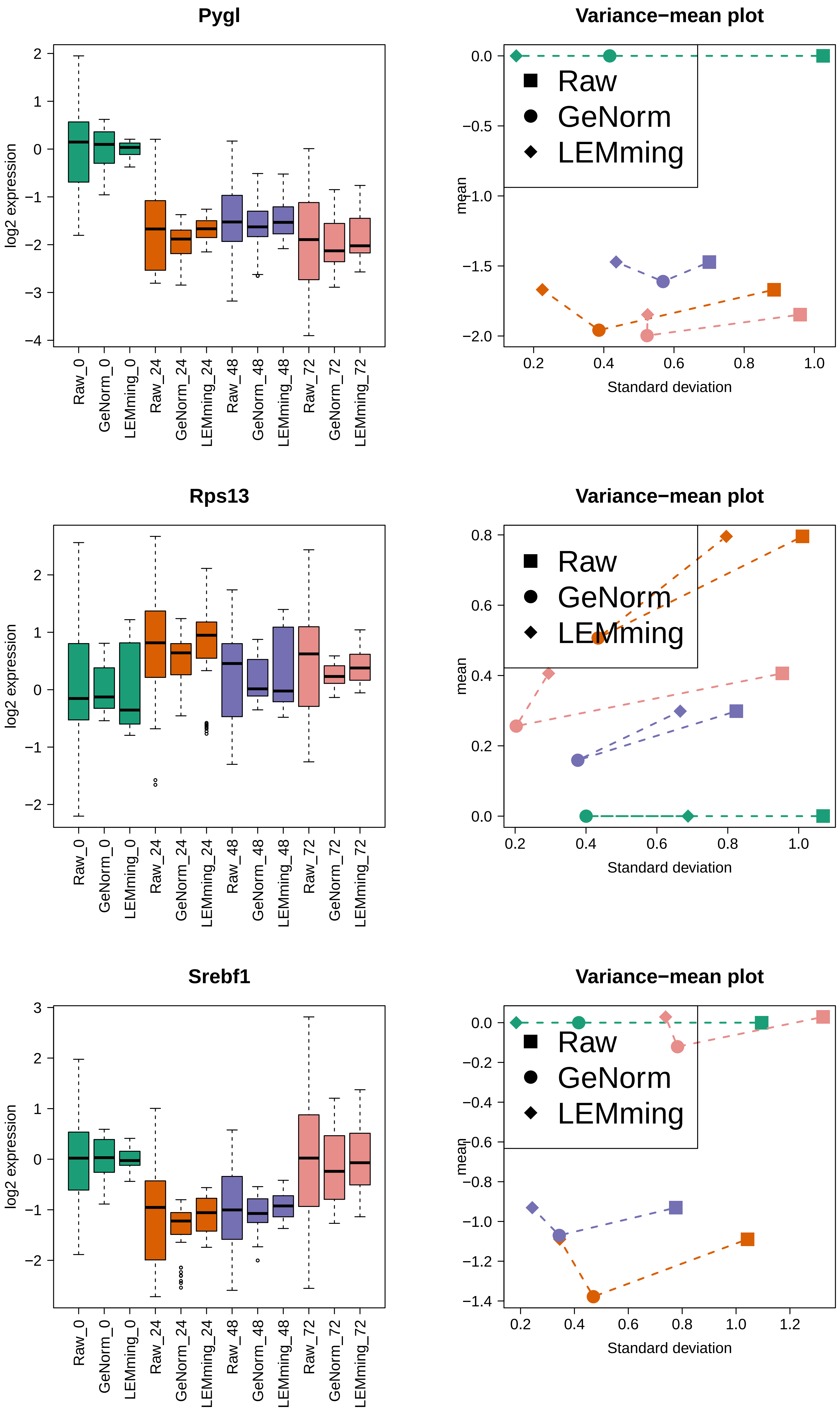
Pygl
log2 expression
Raw_0
GeNorm_0
LEMming_0
Raw_24
GeNorm_24
LEMming_24
Raw_48
GeNorm_48
LEMming_48
Raw_72
GeNorm_72
LEMming_72
Variance−mean plot
Raw
GeNorm
LEMming
mean
Standard deviation
Rps13
log2 expression
Variance−mean plot
Srebf1
log2 expression
Variance−mean plot

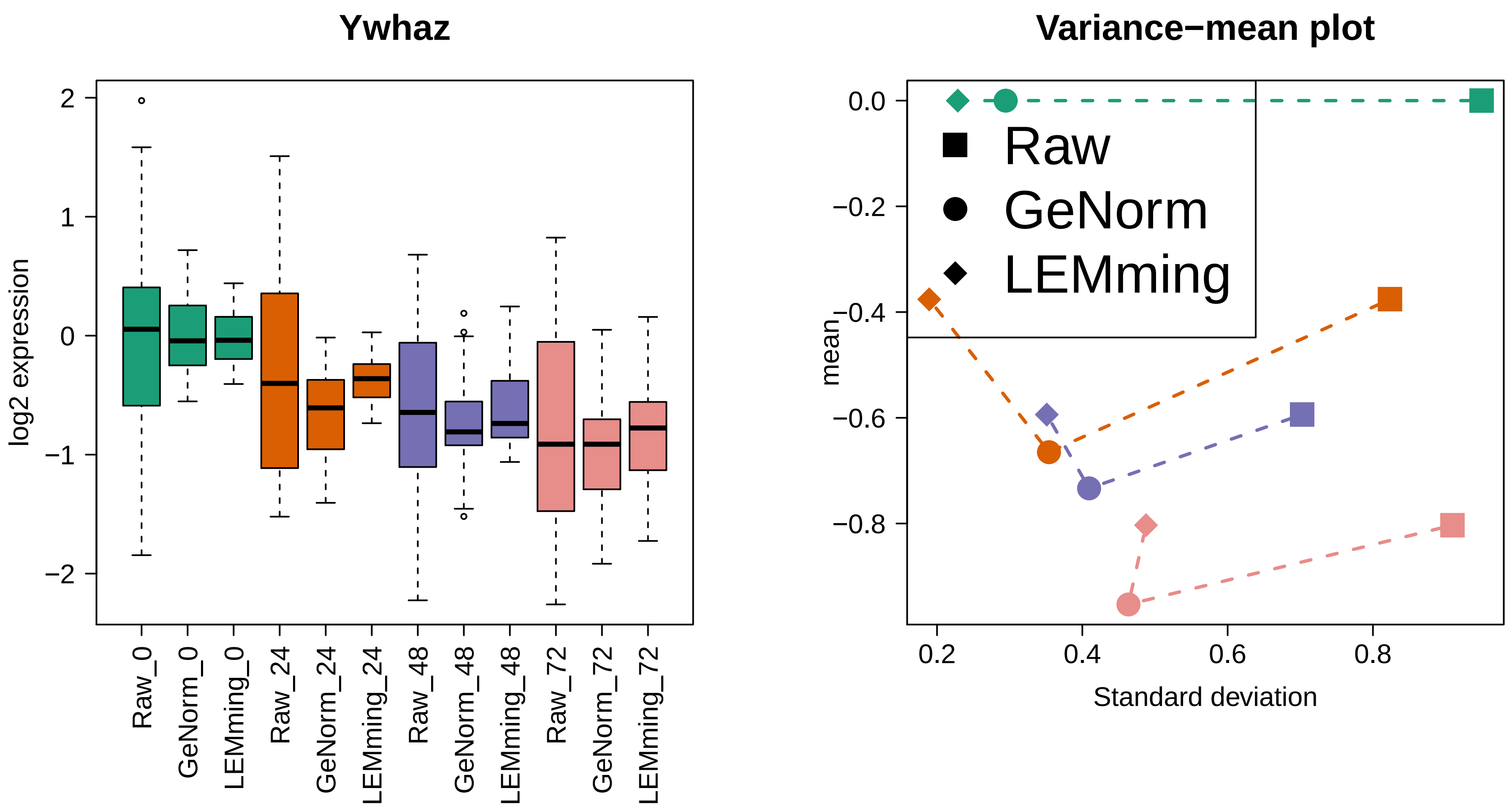
Ywhaz
log2 expression
2
1
0
−1
−2
Raw_0
GeNorm_0
LEMming_0
Raw_24
GeNorm_24
LEMming_24
Raw_48
GeNorm_48
LEMming_48
Raw_72
GeNorm_72
LEMming_72
Variance−mean plot
Raw
GeNorm
LEMming
mean
0.0
−0.2
−0.4
−0.6
−0.8
0.2
0.4
0.6
0.8
Standard deviation
