# Supplementary material for: LEMming: A Linear Error Model to Normalize Parallel Quantitative Real-Time PCR (qPCR) Data as an Alternative to Reference Gene Based Methods
Source: PLoS One. 2015 Sep 1;10(9):e0135852. doi: 10.1371/journal.pone.0135852 (PMC4556681; doi:10.1371/journal.pone.0135852)
Supplement: S4 File — (PDF) [file pone.0135852.s004.pdf]

1 Supplement 6 - Results of data set 3

The average expression stabilities (M-values) of common reference genes (RGs) and pairwise variation values of normalization factors (NFs) in data set 3 are shown in Fig. A and Fig. B. Data set 3 contains the following treatment conditions:

- untreated
- Ligation: observations times of 0h, 24h, 48h and 7d after ligation of the right median hepatic vein
- Ligation PH: observations times of 0h, 24h, 48h and 7d after ligation of the right median hepatic vein and 70% partial hepatectomy
- Ligation PH L: observations times of 0h, 24h, 48h and 7d after ligation of the right median hepatic vein, 70% partial hepatectomy and vaso-active drug L-NAME
- Ligation PH M: observations times of 0h, 24h, 48h and 7d after ligation of the right median hepatic vein, 70% partial hepatectomy and vaso-active drug Molsidomine

Fig. C shows the LEMming processed data of these RGs. Each figure is separated into three parts which correspond to three different zones of obtained liver samples (left: normal zone, middle: border zone, right: obstructed zone). The treatments are arranged in the following order:

- untreated (gray box plot)
- Ligation 0h, Ligation PH 0h, Ligation PH L 0h, Ligation PH M 0h,
- Ligation 24h, Ligation PH 24h, Ligation PH L 24h, Ligation PH M 24h,
- Ligation 48h, Ligation PH 48h, Ligation PH L 48h, Ligation PH M 48h,
- Ligation 7d, Ligation PH 7d, Ligation PH L 7d, Ligation PH M 7d.

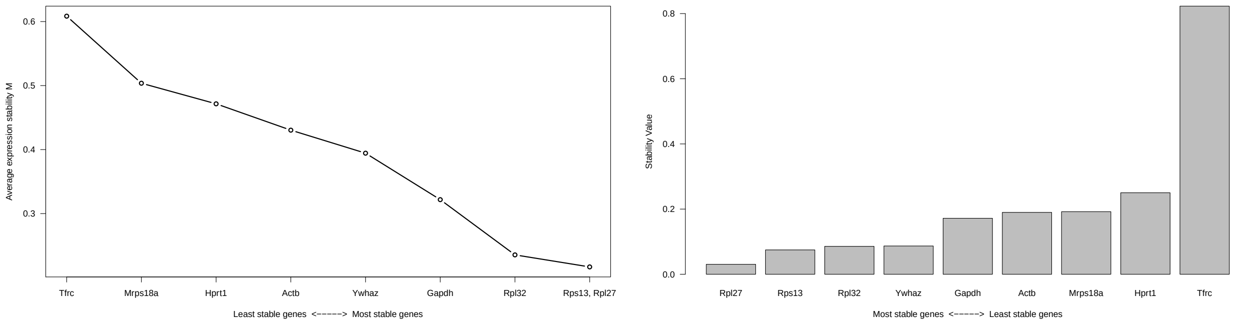

Figure A: Left: *geNorm* Average expression stability values (M) of remaining control genes during stepwise exclusion of the least stable control gene for data set 3 according to Vandesompele et al. 2002. Right: NormFinder stability values according to Anderson et al. 2004.

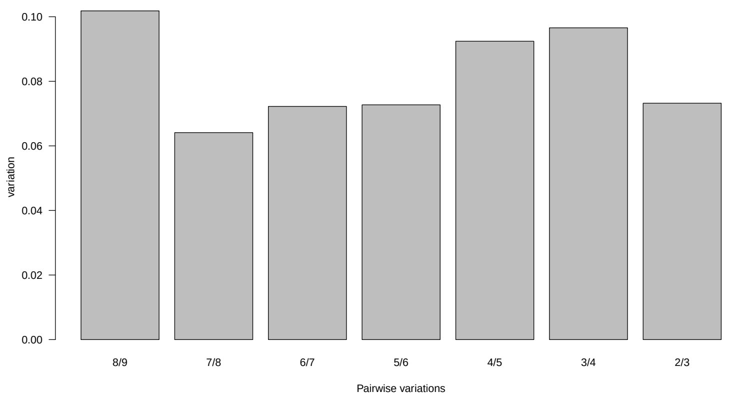

Figure B: *geNorm* pairwise variation values of control genes for data set 3 according to Vandesompele et al. 2002.

1.1 Are the reference genes *Rpl27, Rps13* and *Rpl32* stable expressed?

We used *geNorm* (see Fig. A and selected the genes *Rpl27, Rps13* and *Rpl32* as reference genes (RGs). A t-test with the raw data to test the difference between control condition and treatment conditions results in highly significant differences between the conditions. Table 1 collects the fold changes (FCs) and the p-Values of the t-test for *Rpl27*. The condition partial hepatectomy especially after 24h and 48h result in high FCs up to 6. The expression values of *Rps13* and *Rpl32* show a similar course over the treatment conditions (see Fig. D). This coexpression could explain the low M-values (see Fig. A). However, according to the raw data, if these genes are stable, then there is a huge technical variation. While it is not possible to discriminate here exactly between technical and biological variation, we used two approaches to assess stability of the proposed RGs:

1. We used an external measurement (ssDNA quantification) to estimate the introduced error during cDNA conversion. This is known to introduce about 40% of variation.

2. We made use of the experimental setup, estimated potential systematic errors and removed them. This provides a conservative estimate of the stability of the observed RGs.

Validation by an external measurement: Concluding from the expression pattern of RGs (see Fig. D), under the conditions of partial hepatectomy (PH) after 24h and 48h the overall cDNA content of the samples is expected to be increased compared to the control condition. We examined this hypothesis by measuring the single stranded DNA (ssDNA) in available samples of the normal zone (NZ) using the Quant-iT OliGreen ssDNA Assay Kit (O11492, Life Technologies) (details see Supplement 3). The results are shown in Fig. E. Fig. F summarizes the ssDNA measurements grouped after the variable time. The ssDNA content at 24h and 48h is significantly decreased compared to the control condition and to 0h. Table 1 shows that according to the hypothesis we would expect for NZ samples a huge increase in the ssDNA measurement at 24h and 48h. Consequently, the hypothesis above needs to be rejected. The huge increase in the expression of *Rpl27, Rps13* and *Rpl32* cannot be explained by a change in the overall cDNA content. Thus, the proportion of cDNA of these genes in the total cDNA changes with the experimental condition. We conclude that these genes are not stable expressed under the evaluated experimental conditions.

		NZ		BZ		OZ	
Treatment	Time	FC	p	FC	p	FC	p
untreated		1	1	1	1	1	1
Ligation	0h	1.018	0.907	1.169	0.244	1.168	0.292
Ligation	24h	1.463	0.03	1.308	0.076	2.592	1.18×10^{-7}
Ligation	48h	1.581	0.004	0.953	0.697	2.435	3.96×10^{-8}
Ligation	7d	1.081	0.580	0.899	0.383	1.677	2.18×10^{-4}
Ligation PH	0h	1.456	0.009	0.932	0.562	1.753	6.76×10^{-5}
Ligation PH	24h	4.46	9.09×10^{-17}	3.303	$3. \times 10^{-15}$	6.044	5.22×10^{-22}
Ligation PH	48h	5.63	7.3×10^{-18}	3.709	4.55×10^{-17}	5.539	7.54×10^{-21}
Ligation PH	7d	1.934	1.1×10^{-5}	0.936	0.591	1.847	1.57×10^{-5}
Ligation PH L	0h	1.381	0.024	1.191	0.156	1.947	3.45×10^{-6}
Ligation PH L	24h	2.619	2×10^{-8}	2.615	3.27×10^{-10}	4.47	4.55×10^{-16}
Ligation PH L	48h	4.773	7.5×10^{-16}	3.435	3.65×10^{-14}	5.126	7.78×10^{-18}
Ligation PH L	7d	1.999	2.33×10^{-5}	1.323	0.039	4.263	1.89×10^{-15}
Ligation PH M	0h	1.251	0.114	0.986	0.905	1.405	0.013
Ligation PH M	24h	3.777	1.6×10^{-14}	2.754	2.33×10^{-12}	3.725	2.52×10^{-15}
Ligation PH M	48h	3.691	3.27×10^{-14}	3.375	1.36×10^{-15}	5.122	8.62×10^{-20}
Ligation PH M	7d	1.926	1.24×10^{-5}	1.469	0.002	2.56	6.43×10^{-10}

Table 1: Results of t-tests (FC: fold change, p: p-Value) with raw data for *Rpl27* comparing treatment conditions with the according untreated in three different zones NZ: normal zone, BZ: border zone, OZ: obstructed zone.

Multivariable regression approach: According to the ssDNA measurement a batch effect related to the variable *observation time after ligation* is observed. This effect is part of the treatment effect (Δ_T) and can be excluded with the LEMming error model as batch effect ($\tilde{\epsilon}$). The removal of batch effects and systematic errors shifts results in a shifted mean compared to the raw data. The result of the normalization with removed batch effects is shown in Fig. G. The treatment effect (see Supplement 7 worksheet *LEM_ Treatmenteffect*) is reduced by the removal and so does the fold changes of the common RGs. But still there are significant alterations with the treatment conditions. Thus, even with the removed batch effect the common reference genes are not stable expressed.
Furthermore, the removal of such batch effects is inaccurate as long as the exact quantity of the batch effect is unknown. In this case there might be a real effect of the *observation time after ligation* on the expression values. The average ΔC_t value between 0h and 24h is -1.24 which corresponds to a fold change of 2.37. In contrast the ssDNA measurement shows only a difference of around 12% between 0h and 24h (see Fig. F). Thus, we would expect that the batch effect of the time variable is overestimated. Here we conclude, that a removal of batch effects needs a careful validation by an external measurement such as a ssDNA quantification.

References

[1] Assy N, Gong Y, Zhang M, Pettigrew N, Pashniak D, et al. (1998) Use of proliferating cell nuclear antigen as a marker of liver regeneration after partial hepatectomy in rats. Journal of Laboratory and Clinical Medicine 131: 251–256.

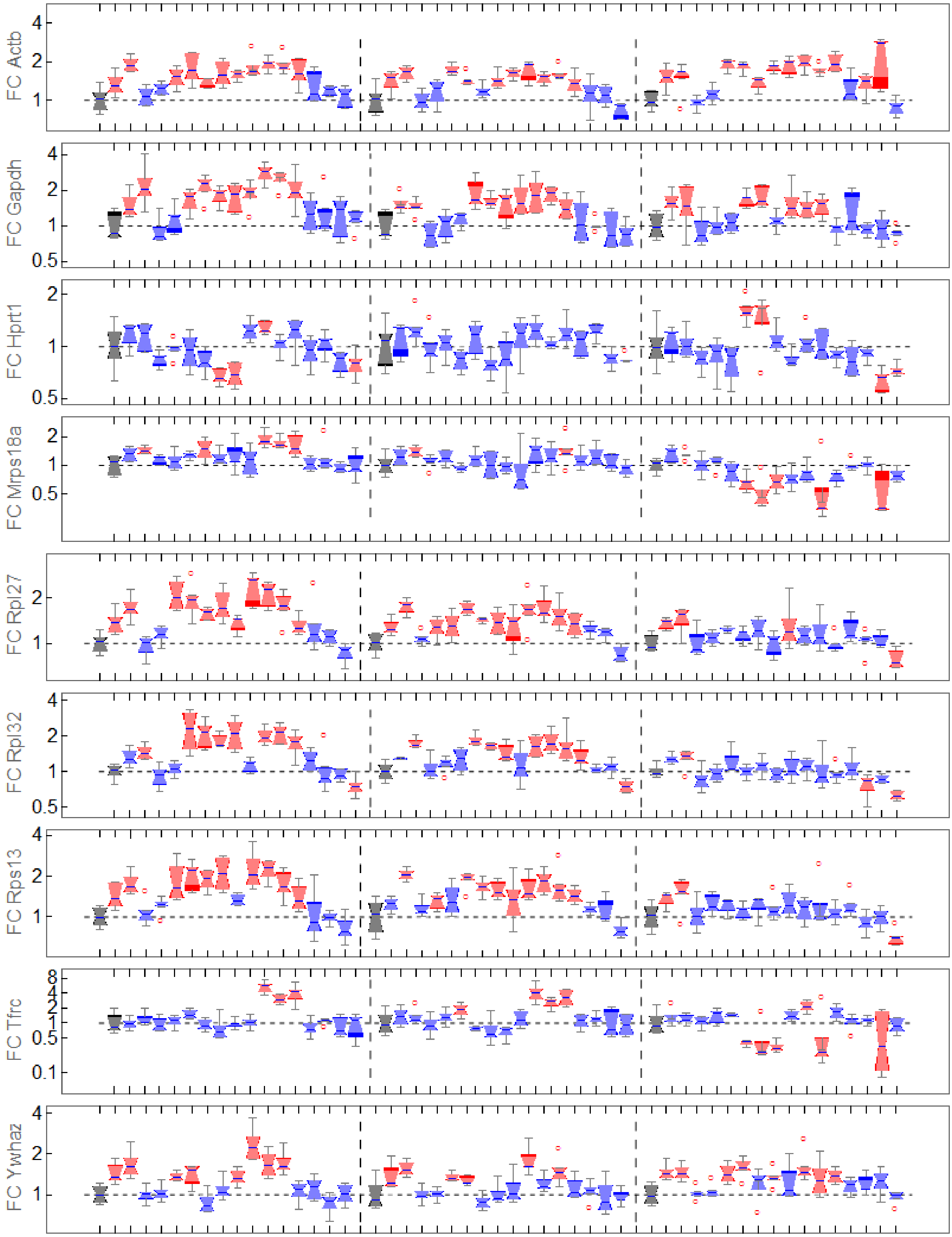

Figure C: Common reference genes in data set 3 evaluated with LEMming. Boxplots of the untreated conditions are black, boxplots of treatment conditions that are not statistically differential expressed compared to untreated are blue and boxplots of treatments with statistically differential expressed measurements are red. Measurements that are outliers are marked by red circles.

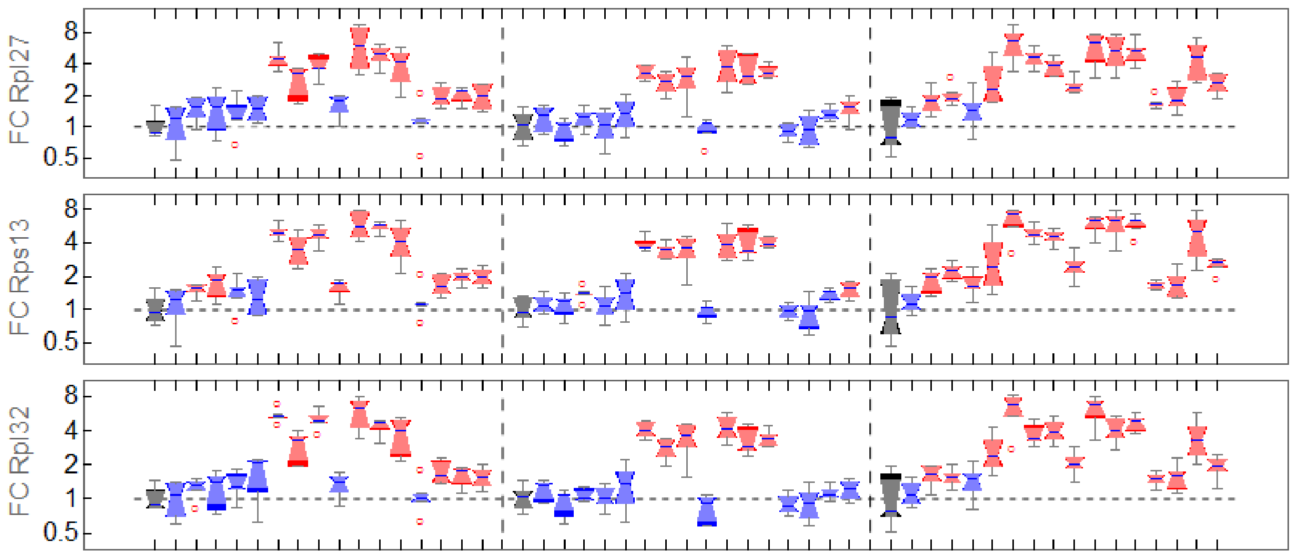

Figure D: Raw data of the genes *Rpl27*, *Rps13* and *Rpl32* in data set 3. Boxplots of the untreated conditions are black, boxplots of treatment conditions that are not statistically differential expressed compared to untreated are blue and boxplots of treatments with statistically differential expressed measurements are red. Measurements that are outliers are marked by red circles. (left: normal zone, middle: border zone, right: obstructed zone)

	Sample	Time	Replicate	Treatment	cDNA	Experiment	ssDNA
9	RHV106-NZ	0	1	L+PH70	0	RHV	453.8653
10	RHV094-NZ	48	1	L+PH70	0	RHV	445.7278
11	RHV102-NZ	48	1	L+PH70	0	RHV	412.2588
21	RHV111-NZ	24	1	L+PH70	0	RHV	382.5961
22	RHV053-NZ	-1	1	Control	0	RHV	458.5904
23	RHV110-NZ	168	1	L+PH70	0	RHV	425.3839
32	RHV105-NZ	0	1	L+PH70	0	RHV	480.7718
33	RHV056-NZ	-1	1	Control	0	RHV	438.7715
35	RHV055-NZ	-1	1	Control	0	RHV	400.7088
44	RHV113-NZ	168	1	L+PH70	0	RHV	431.4214
45	RHV107-NZ	168	1	L+PH70	0	RHV	381.8086
46	RHV102-NZ	48	2	L+PH70	0	RHV	412.2588
55	RHV058-NZ	-1	1	Control	0	RHV	418.8214
56	RHV091-NZ	168	1	L+PH70	0	RHV	442.8403
57	RHV104-NZ	24	1	L+PH70	0	RHV	392.7025
58	RHV108-NZ	168	1	L+PH70	0	RHV	463.9716
67	RHV099-NZ	48	1	L+PH70	0	RHV	392.9650
68	RHV109-NZ	24	1	L+PH70	0	RHV	414.3588
69	RHV057-NZ	-1	1	Control	0	RHV	453.0778
70	RHV101-NZ	0	1	L+PH70	0	RHV	442.4465
79	RHV103-NZ	24	1	L+PH70	0	RHV	390.9962
80	RHV095-NZ	0	1	L+PH70	0	RHV	422.3651
81	RHV097-NZ	48	1	L+PH70	0	RHV	411.6026
82	RHV114-NZ	24	1	L+PH70	0	RHV	418.0339
91	RHV115-NZ	24	1	L+PH70	0	RHV	392.7025
92	RHV092-NZ	168	1	L+PH70	0	RHV	394.2775
93	RHV093-NZ	48	1	L+PH70	0	RHV	404.1213
94	RHV096-NZ	0	1	L+PH70	0	RHV	435.4902

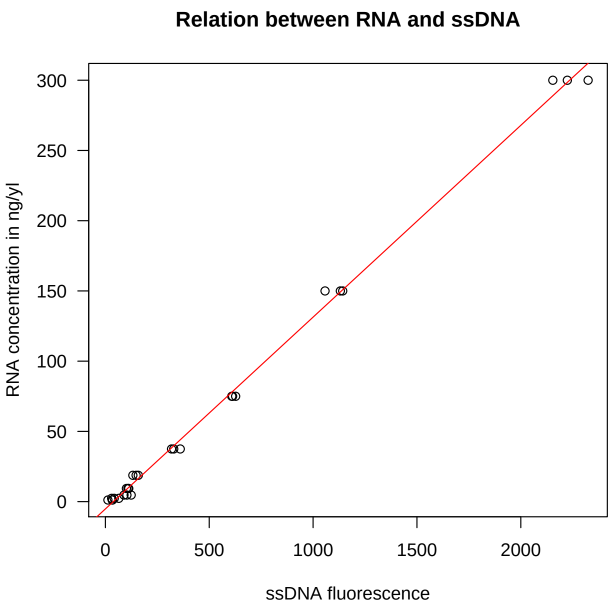

Figure E: Left: Table of single stranded DNA (ssDNA) measurement in available samples for DS3 using the Quant-iT OliGreen ssDNA Assay Kit (O11492, Life Technologies). Right: Calibration curve for ssDNA obtained by a dilution series with a sample of known RNA quantity.

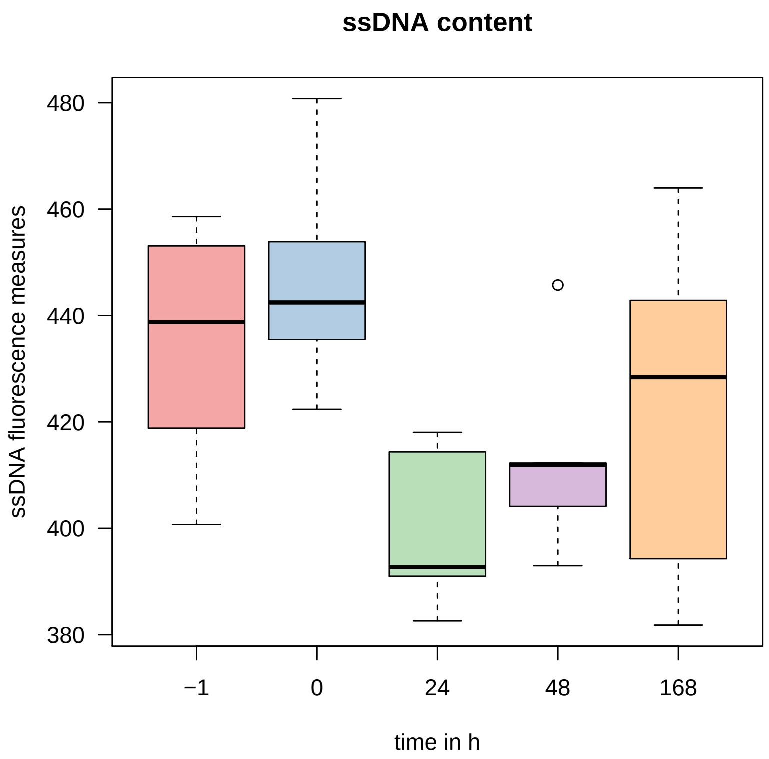

Figure F: Results of the ssDNA measurement (see Fig. E left) grouped after the variable time. The control condition is marked by -1.

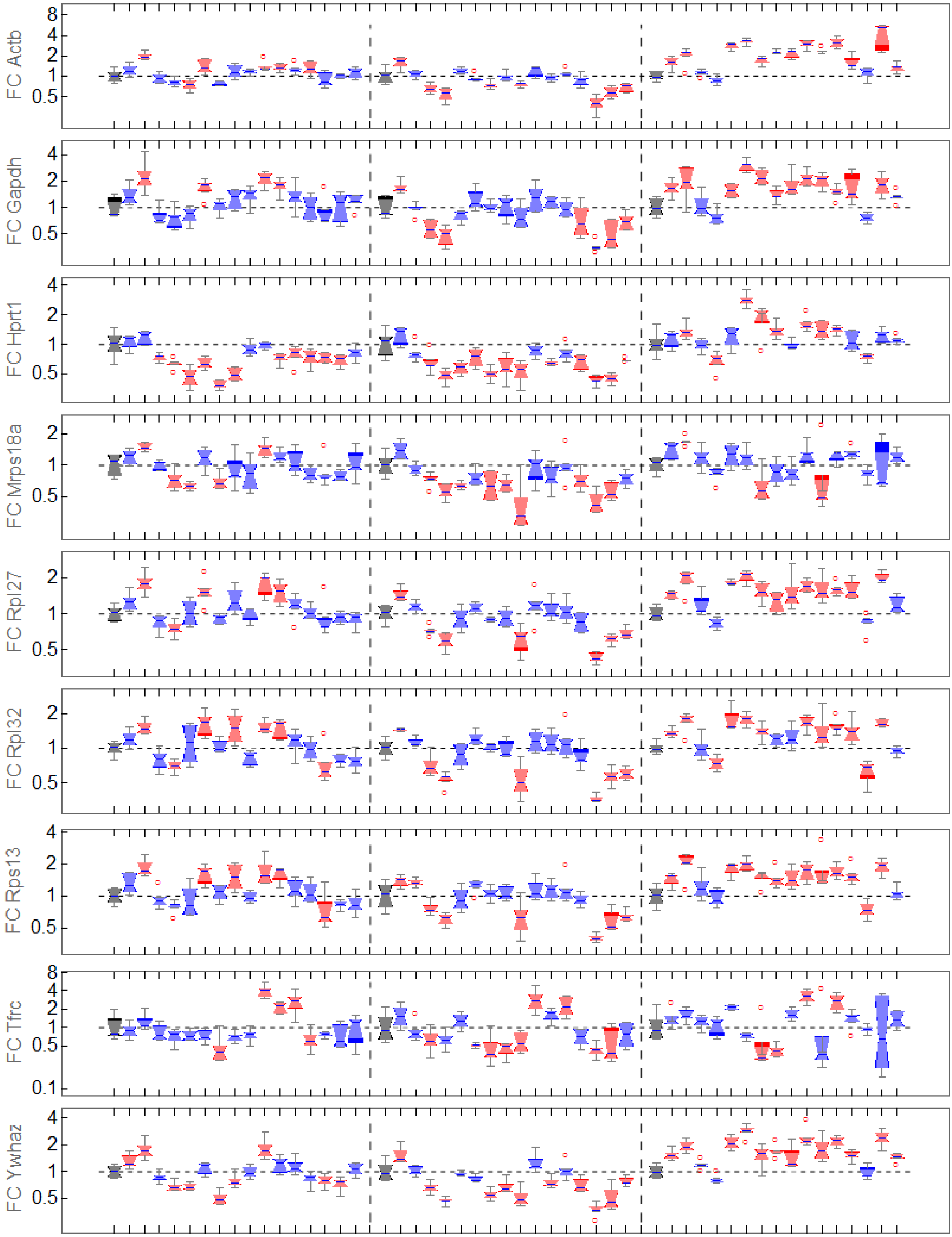

Figure G: LEMming normalized data with excluded batch effects of the variable time after ligation for common reference genes in DS3. Boxplots of the untreated conditions are black, boxplots of treatment conditions that are not statistically differential expressed compared to untreated are blue and boxplots of treatments with statistically differential expressed measurements are red. Measurements that are outliers are marked by red circles. (left: normal zone, middle: border zone, right: obstructed zone)

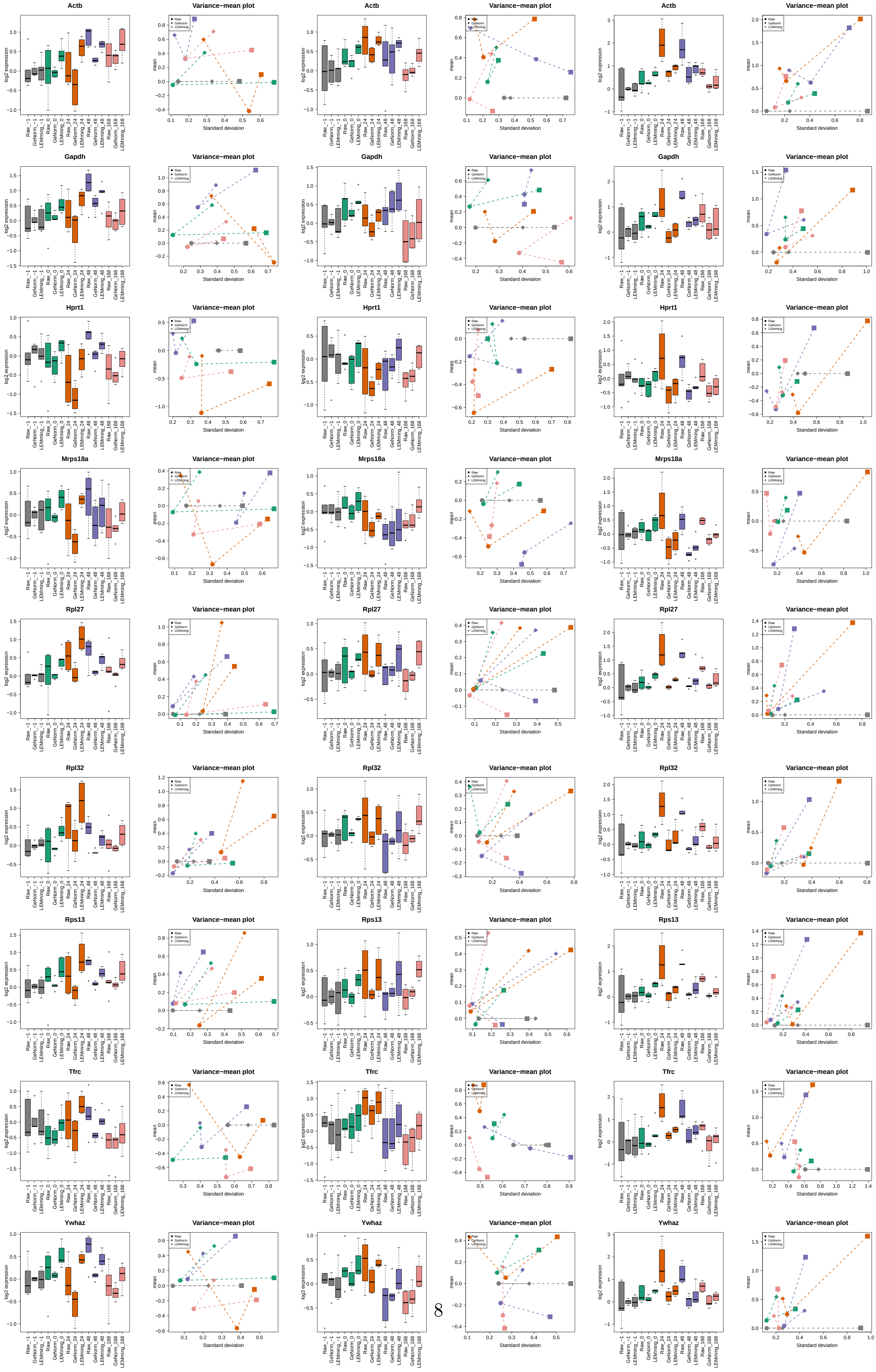

Figure H: Comparison of common RGs between raw data, *geNorm* and LEMming normalized data under the condition ligation. Left: NZ, middle: BZ, right OZ.

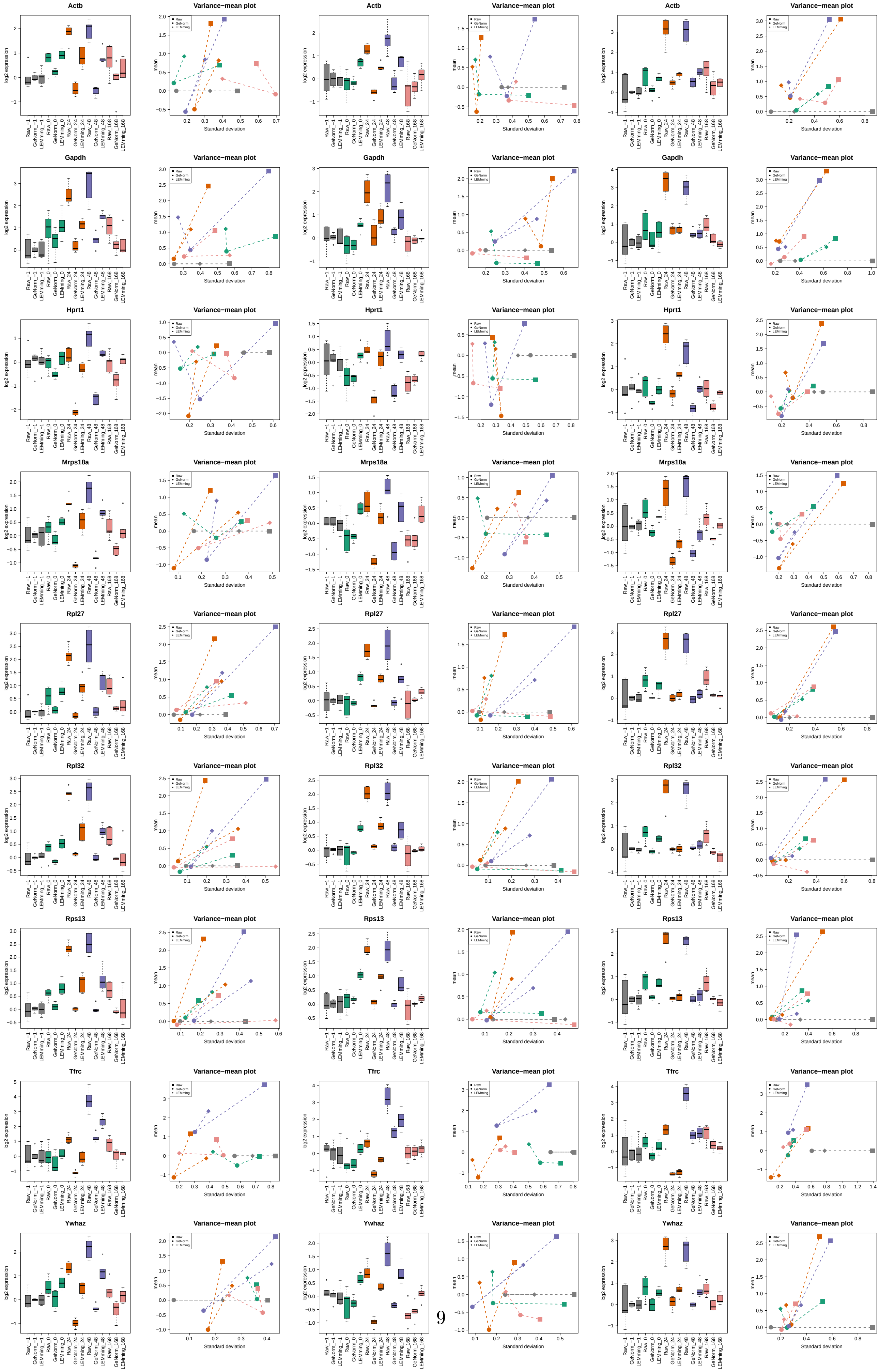

Figure I: Comparison of common RGs between raw data, *geNorm* and LEMming normalized data under the condition ligation and partial hepatectomy (PH). Left: NZ, middle: BZ, right OZ.

Ligation

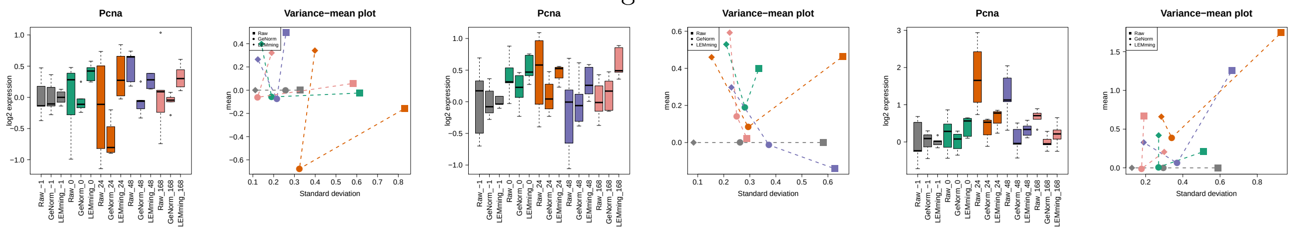

Ligation PH

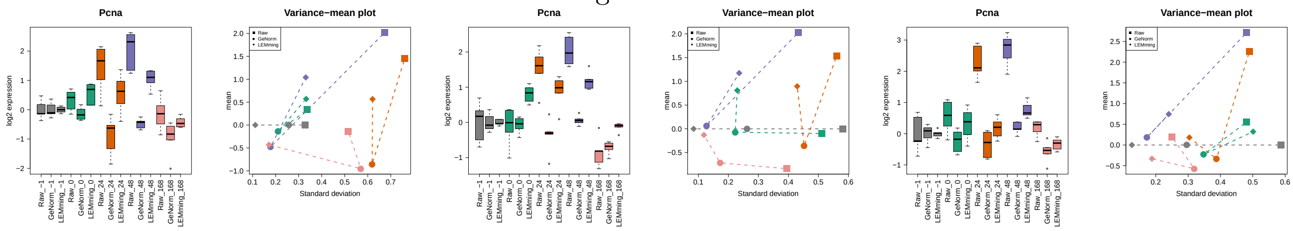

Ligation PH-L-NAME

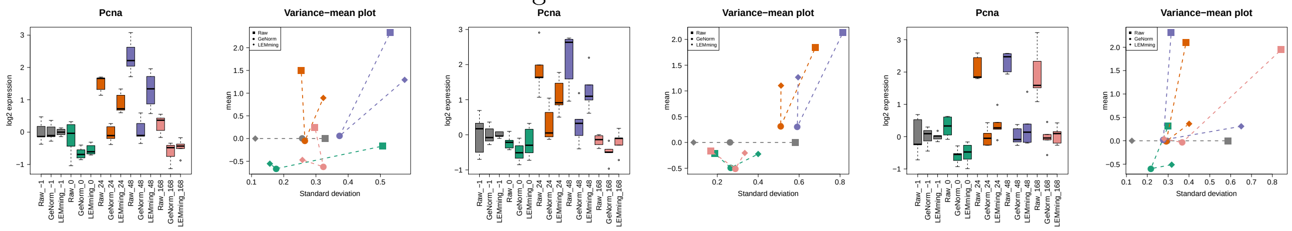

Ligation PH-Molsidomine

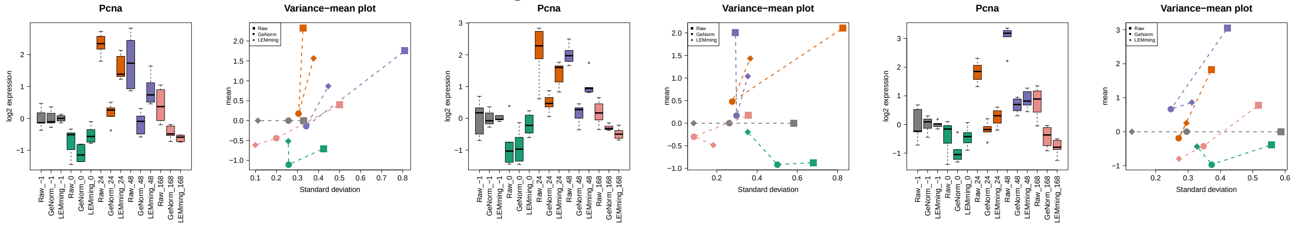

Figure J: Comparison of marker gene *Pcna* for partial hepatectomy [1] between raw data, *geNorm* and LEMming normalized data. Left: NZ, middle: BZ, right OZ.
